# Different doses, durations and modes of delivery of nicotine replacement therapy for smoking cessation

**DOI:** 10.1002/14651858.CD013308.pub2

**Published:** 2023-06-19

**Authors:** Annika Theodoulou, Samantha C Chepkin, Weiyu Ye, Thomas R Fanshawe, Chris Bullen, Jamie Hartmann-Boyce, Jonathan Livingstone-Banks, Anisa Hajizadeh, Nicola Lindson

**Affiliations:** Nuffield Department of Primary Care Health SciencesUniversity of OxfordOxfordUK; NHS Hertfordshire and West Essex Integrated Care BoardWelwyn Garden CityUK; Oxford University Clinical Academic Graduate SchoolUniversity of OxfordOxfordUK; National Institute for Health InnovationUniversity of AucklandAucklandNew Zealand

**Keywords:** Humans, Delivery of Health Care, Nicotine, Nicotinic Agonists, Nicotinic Agonists/adverse effects, Smoking Cessation, Smoking Cessation/methods, Tobacco Use Cessation Devices

## Abstract

**Background:**

Nicotine replacement therapy (NRT) aims to replace nicotine from cigarettes. This helps to reduce cravings and withdrawal symptoms, and ease the transition from cigarette smoking to complete abstinence. Although there is high‐certainty evidence that NRT is effective for achieving long‐term smoking abstinence, it is unclear whether different forms, doses, durations of treatment or timing of use impacts its effects.

**Objectives:**

To determine the effectiveness and safety of different forms, deliveries, doses, durations and schedules of NRT, for achieving long‐term smoking cessation.

**Search methods:**

We searched the Cochrane Tobacco Addiction Group trials register for papers mentioning NRT in the title, abstract or keywords, most recently in April 2022.

**Selection criteria:**

We included randomised trials in people motivated to quit, comparing one type of NRT use with another. We excluded studies that did not assess cessation as an outcome, with follow‐up of fewer than six months, and with additional intervention components not matched between arms. Separate reviews cover studies comparing NRT to control, or to other pharmacotherapies.

**Data collection and analysis:**

We followed standard Cochrane methods. We measured smoking abstinence after at least six months, using the most rigorous definition available. We extracted data on cardiac adverse events (AEs), serious adverse events (SAEs) and study withdrawals due to treatment.

**Main results:**

We identified 68 completed studies with 43,327 participants, five of which are new to this update. Most completed studies recruited adults either from the community or from healthcare clinics. We judged 28 of the 68 studies to be at high risk of bias. Restricting the analysis only to those studies at low or unclear risk of bias did not significantly alter results for any comparisons apart from the preloading comparison, which tested the effect of using NRT prior to quit day whilst still smoking.

There is high‐certainty evidence that combination NRT (fast‐acting form plus patch) results in higher long‐term quit rates than single form (risk ratio (RR) 1.27, 95% confidence interval (CI) 1.17 to 1.37; I^2^ = 12%; 16 studies, 12,169 participants). Moderate‐certainty evidence, limited by imprecision, indicates that 42/44 mg patches are as effective as 21/22 mg (24‐hour) patches (RR 1.09, 95% CI 0.93 to 1.29; I^2^ = 38%; 5 studies, 1655 participants), and that 21 mg patches are more effective than 14 mg (24‐hour) patches (RR 1.48, 95% CI 1.06 to 2.08; 1 study, 537 participants). Moderate‐certainty evidence, again limited by imprecision, also suggests a benefit of 25 mg over 15 mg (16‐hour) patches, but the lower limit of the CI encompassed no difference (RR 1.19, 95% CI 1.00 to 1.41; I^2^ = 0%; 3 studies, 3446 participants).

Nine studies tested the effect of using NRT prior to quit day (preloading) in comparison to using it from quit day onward. There was moderate‐certainty evidence, limited by risk of bias, of a favourable effect of preloading on abstinence (RR 1.25, 95% CI 1.08 to 1.44; I^2^ = 0%; 9 studies, 4395 participants).

High‐certainty evidence from eight studies suggests that using either a form of fast‐acting NRT or a nicotine patch results in similar long‐term quit rates (RR 0.90, 95% CI 0.77 to 1.05; I^2^ = 0%; 8 studies, 3319 participants).

We found no clear evidence of an effect of duration of nicotine patch use (low‐certainty evidence); duration of combination NRT use (low‐ and very low‐certainty evidence); or fast‐acting NRT type (very low‐certainty evidence).

Cardiac AEs, SAEs and withdrawals due to treatment were all measured variably and infrequently across studies, resulting in low‐ or very low‐certainty evidence for all comparisons. Most comparisons found no clear evidence of an effect on these outcomes, and rates were low overall. More withdrawals due to treatment were reported in people using nasal spray compared to patches in one study (RR 3.47, 95% CI 1.15 to 10.46; 1 study, 922 participants; very low‐certainty evidence) and in people using 42/44 mg patches in comparison to 21/22 mg patches across two studies (RR 4.99, 95% CI 1.60 to 15.50; I^2^ = 0%; 2 studies, 544 participants; low‐certainty evidence).

**Authors' conclusions:**

There is high‐certainty evidence that using combination NRT versus single‐form NRT and 4 mg versus 2 mg nicotine gum can result in an increase in the chances of successfully stopping smoking. Due to imprecision, evidence was of moderate certainty for patch dose comparisons. There is some indication that the lower‐dose nicotine patches and gum may be less effective than higher‐dose products. Using a fast‐acting form of NRT, such as gum or lozenge, resulted in similar quit rates to nicotine patches. There is moderate‐certainty evidence that using NRT before quitting may improve quit rates versus using it from quit date only; however, further research is needed to ensure the robustness of this finding. Evidence for the comparative safety and tolerability of different types of NRT use is limited. New studies should ensure that AEs, SAEs and withdrawals due to treatment are reported.

## Plain language summary

**What is the best way to use nicotine replacement therapy to quit smoking?**

**Key messages**

Using a combination of nicotine patches together with another type of nicotine replacement therapy (NRT) (such as gum or lozenge) is more likely to help people quit smoking than if they used one type of NRT alone. We also found that people who smoke have the same chance of quitting successfully whether they use a nicotine patch or another type of NRT, such as gum, lozenge or nasal spray.

More high‐quality studies on different NRT patch doses, durations of NRT use, types of fast‐acting NRT, and NRT use prior to quit day are needed to know which treatments work best to help people quit smoking. These studies should report safety outcomes and withdrawals due to treatment.

**What is nicotine replacement therapy?**

Nicotine replacement therapy (NRT) is a medicine that delivers nicotine to the brain. It is available as skin patches, chewing gum, nasal and oral sprays, inhalers, lozenges and tablets. The aim of NRT is to replace the nicotine that people who smoke usually get from cigarettes, so the urge to smoke is reduced and they can stop smoking completely. We know that NRT improves a person's chances of stopping smoking, and that people use it to quit.

**What did we want to find out?**

NRT can be taken in many different forms, in different doses and for varying amounts of time. Some people start using NRT before they quit, while other people wait until quit day. This review looks at the different forms, doses, durations and schedules of NRT used to help people quit smoking, so we can better understand which of these work best to help people quit smoking for six months or longer. We also wanted to find out if any of these treatments were associated with cardiac (heart‐related) or serious unwanted effects, and if anyone stopped participating in a study due to the NRT treatment they were advised to use.

**What did we do?**

We searched for studies that looked at the use of NRT to help people quit smoking and that followed people up for at least six months.

**What we found**

We found 68 completed studies conducted in 43,327 participants. Most participants were adults who wanted to quit smoking.

**Main results**

People who smoke have the same chances of quitting successfully whether they use a nicotine patch to quit or another type of NRT, such as gum, lozenge or nasal spray. Using nicotine patches together with another type of NRT (such as gum or lozenge) made it 17% to 37% more likely that a person would successfully stop smoking than if they used one type of NRT alone.

People who used higher‐dose nicotine patches (25 mg patches worn for 16 hours, or 21 mg patches worn for 24 hours) were more likely to quit smoking compared to those using lower‐dose patches (15 mg patches worn for 16 hours or 14 mg patches worn for 24 hours). However, there was not any clear evidence to suggest that people using 42 mg or 44 mg patches were more likely to quit than people using 21 mg or 22 mg (24‐hour) patches.

Starting to use NRT before a quit day may help more people to quit than only using it after a quit day, but more evidence is needed to strengthen this conclusion.

We also looked at how long NRT should be used for, whether NRT should be used on a schedule or on demand as craved, and whether more people stop smoking when NRT is provided for free versus if they have to pay for it. More research is needed to answer these questions.

Most studies did not look at the safety of NRT. Where studies did look at safety, they found that very few people experienced negative effects.

**How reliable are these results?**

There is high‐certainty evidence that:

‐ combination NRT works better than a single form of NRT; and  ‐ there is no difference in effect between different types of NRT (such as gum or patch).

This means that future research is very unlikely to change our conclusions. This is because the evidence is based on many participants and on well‐conducted studies.

However, the certainty of the evidence was moderate, low or very low for all the other questions we considered. This means that our findings may change as new research is carried out. In most cases, this is because there were not enough studies, there were problems with the design of studies that do exist, and/or these studies were too small.

In terms of the safety of different ways of using NRT, we rated the evidence for this outcome to be of low or very low certainty because many studies did not report on safety. Large studies covered in a separate review show high‐certainty evidence that NRT is safe to use for quitting smoking.

**How up to date is this evidence?**

This review updates our previous review. The evidence is up to date to April 2022.

## Summary of findings

**Summary of findings 1:** Combination compared to single‐form nicotine replacement therapy for smoking cessation

**Combination compared to single‐form nicotine replacement therapy (NRT) for smoking cessation**						
**Patient or population:** people who smoke **Setting:** any; studies conducted in: Australasia, China, Europe, USA **Intervention:** combination NRT (nicotine patch plus a fast‐acting form of NRT) **Comparison:** single‐form NRT						
**Outcomes**	Anticipated absolute effects^*^ (95% CI)		**Relative effect (95% CI)**	**№ of participants (studies)**	**Certainty of the evidence (GRADE)**	**Comments**
	**Risk with single‐form NRT**	**Risk with combination NRT**				
Smoking cessation	Study population		RR 1.27 (1.17 to 1.37)	12,169 (16 RCTs)	⊕⊕⊕⊕ High^a^	‐
	137 per 1000	174 per 1000 (160 to 187)				
Overall serious adverse events	Study population		RR 4.44 (0.76 to 25.85)	2888 (5 RCTs)	⊕⊕⊝⊝ Low^b^	‐
	1 per 1000	3 per 1000 (1 to 18)				
Treatment withdrawals	Study population		RR 1.12 (0.57 to 2.20)	3070 (5 RCTs)	⊕⊝⊝⊝ Very low^b,c^	‐
	12 per 1000	14 per 1000 (7 to 27)				
*The risk in the intervention group (and its 95% confidence interval) is based on the assumed risk in the comparison group and the **relative effect** of the intervention (and its 95% CI). **CI:** confidence interval; **NRT:** nicotine replacement therapy; **RCT:** randomised controlled trial; **RR:** risk ratio						
**GRADE Working Group grades of evidence** **High certainty:** we are very confident that the true effect lies close to that of the estimate of the effect. **Moderate certainty:** we are moderately confident in the effect estimate: the true effect is likely to be close to the estimate of the effect, but there is a possibility that it is substantially different. **Low certainty:** our confidence in the effect estimate is limited: the true effect may be substantially different from the estimate of the effect. **Very low certainty:** we have very little confidence in the effect estimate: the true effect is likely to be substantially different from the estimate of effect.						

^a^We rated most studies at low or unclear risk of bias. We did not downgrade the certainty of the evidence, as limiting the analysis only to studies we judged to be at low risk of bias resulted in a consistent effect estimate and 95% confidence interval. ^b^Downgraded by two levels due to imprecision: fewer than 100 events overall and confidence intervals encompass clinically significant harms as well as clinically significant benefits. ^c^Downgraded one level due to inconsistency: moderate unexplained statistical heterogeneity (I^2^ = 73%).

**Summary of findings 2:** Longer compared to shorter duration of combination nicotine replacement therapy for smoking cessation

**Longer compared to shorter duration of combination nicotine replacement therapy for smoking cessation**						
**Patient or population:** people who smoke **Setting:** any; studies conducted in: USA **Intervention:** longer duration combination NRT (nicotine patch plus a fast‐acting form of NRT) **Comparison:** shorter duration combination NRT (nicotine patch plus a fast‐acting form of NRT)						
**Outcomes**	Anticipated absolute effects^*^ (95% CI)		**Relative effect (95% CI)**	**№ of participants (studies)**	**Certainty of the evidence (GRADE)**	**Comments**
	**Risk with shorter duration NRT**	**Risk with longer duration NRT**				
Smoking cessation ‐ 16 weeks versus 8 weeks	Study population		RR 0.96 (0.75 to 1.23)	637 (1 RCT)	⊕⊝⊝⊝ Very low^a,b^	‐
	285 per 1000	274 per 1000 (214 to 351)				
Smoking cessation ‐ 6 weeks versus 2 weeks	Study population		RR 1.11 (0.94 to 1.31)	987 (1 RCT)	⊕⊕⊝⊝ Low^a,c^	‐
	351 per 1000	390 per 1000 (330 to 460)				
Overall SAEs ‐ 26 weeks versus 8 weeks	Study population		RR 1.63 (0.60 to 4.42)	544 (1 RCT)	⊕⊝⊝⊝ Very low^a,d^	‐
	22 per 1000	36 per 1000 (13 to 99)				
Overall SAEs ‐ 16 weeks versus 8 weeks	Study population		Not estimable	637 (1 RCT)	⊕⊝⊝⊝ Very low^a,d^	No events in either arm
	Not estimable	Not estimable				
Overall SAEs ‐ 6 weeks versus 2 weeks	Study population		Not estimable	987 (1 RCT)	⊕⊝⊝⊝ Very low^a,d^	No events in either arm
	Not estimable	Not estimable				
Treatment withdrawals	Study population		n/a	0 (0 RCTs)	n/a	None of our included studies reported usable data on this outcome.
	n/a	n/a				
*The risk in the intervention group (and its 95% confidence interval) is based on the assumed risk in the comparison group and the **relative effect** of the intervention (and its 95% CI). **CI:** confidence interval; **n/a**: not applicable; **NRT:** nicotine replacement therapy; **RCT:** randomised controlled trial; **RR:** risk ratio; **SAEs:** serious adverse events						
**GRADE Working Group grades of evidence** **High certainty:** we are very confident that the true effect lies close to that of the estimate of the effect. **Moderate certainty:** we are moderately confident in the effect estimate: the true effect is likely to be close to the estimate of the effect, but there is a possibility that it is substantially different. **Low certainty:** our confidence in the effect estimate is limited: the true effect may be substantially different from the estimate of the effect. **Very low certainty:** we have very little confidence in the effect estimate: the true effect is likely to be substantially different from the estimate of effect.						

^a^Downgraded by one level due to risk of bias: we judged the one included study to be at high risk of bias. ^b^Downgraded by two levels for imprecision: fewer than 300 events and confidence intervals encompass clinically significant benefit as well as clinically significant harm. ^c^Downgraded by one level due to imprecision: confidence intervals encompass no clinically significant difference between groups as well as clinically significant benefit. ^d^Downgraded by two levels due to imprecision: fewer than 100 events overall.

**Summary of findings 3:** Higher‐dose compared to lower‐dose nicotine patch for smoking cessation

**Higher‐dose compared to lower‐dose nicotine patch for smoking cessation**						
**Patient or population:** people who smoke **Setting:** any; studies conducted in: Australasia, Europe, USA **Intervention:** higher‐dose nicotine patch **Comparison:** lower‐dose nicotine patch						
**Outcomes**	Anticipated absolute effects^*^ (95% CI)		**Relative effect (95% CI)**	**№ of participants (studies)**	**Certainty of the evidence (GRADE)**	**Comments**
	**Risk with lower‐dose nicotine patch**	**Risk with higher‐dose nicotine patch**				
Smoking cessation ‐ 42/44 mg versus 21/22 mg (24‐hour patches)	Study population		RR 1.09 (0.93 to 1.29)	1655 (5 RCTs)	⊕⊕⊕⊝ Moderate^a^	‐
	238 per 1000	260 per 1000 (222 to 307)				
Smoking cessation ‐ 25 mg versus 15 mg (16‐hour patches)	Study population		RR 1.19 (1.00 to 1.41)	3446 (3 RCTs)	⊕⊕⊕⊝ Moderate^a,b^	‐
	123 per 1000	146 per 1000 (123 to 173)				
Smoking cessation ‐ 21 mg versus 14 mg (24‐hour patches)	Study population		RR 1.48 (1.06 to 2.08)	537 (1 RCT)	⊕⊕⊕⊝ Moderate^c^	‐
	167 per 1000	248 per 1000 (177 to 348)				
Overall SAEs ‐ 42/44 mg versus 21/22 mg (24 hr patches)	Study population		RR 5.01 (0.87 to 28.82)	1023 (2 RCTs)	⊕⊕⊝⊝ Low^d,e^	‐
	2 per 1000	10 per 1000 (2 to 56)				
Overall SAEs ‐ 21 mg versus 14 mg (24‐hour patches)	Study population		Not estimable	537 (1 RCT)	⊕⊕⊝⊝ Low^f^	No events in either arm
	Not estimable	Not estimable				
Treatment withdrawals ‐ 42/44 mg versus 21/22 mg (24‐hour patches)	Study population		RR 4.99 (1.60 to 15.50)	554 (2 RCTs)	⊕⊕⊝⊝ Low^e,f^	‐
	11 per 1000	54 per 1000 (17 to 168)				
Treatment withdrawals ‐ 21 mg versus 14 mg (24‐hour patches)	Study population		RR 0.77 (0.36 to 1.64)	537 (1 RCT)	⊕⊕⊝⊝ Low^d^	‐
	55 per 1000	42 per 1000 (20 to 89)				
*The risk in the intervention group (and its 95% confidence interval) is based on the assumed risk in the comparison group and the **relative effect** of the intervention (and its 95% CI). **CI:** confidence interval; **NRT:** nicotine replacement therapy; **RCT:** randomised controlled trial; **RR:** risk ratio; **SAEs:** serious adverse events						
**GRADE Working Group grades of evidence** **High certainty:** we are very confident that the true effect lies close to that of the estimate of the effect. **Moderate certainty:** we are moderately confident in the effect estimate: the true effect is likely to be close to the estimate of the effect, but there is a possibility that it is substantially different. **Low certainty:** our confidence in the effect estimate is limited: the true effect may be substantially different from the estimate of the effect. **Very low certainty:** we have very little confidence in the effect estimate: the true effect is likely to be substantially different from the estimate of effect.						

^a^Downgraded by one level due to imprecision: confidence intervals encompass no difference as well as a clinically significant difference. ^b^We rated most studies at low or unclear risk of bias. We did not downgrade the certainty of the evidence, as limiting the analysis only to studies we judged to be at low risk of bias resulted in a consistent effect estimate and 95% confidence interval. ^c^Downgraded by one level due to imprecision: fewer than 300 events overall. ^d^Downgraded by two levels due to imprecision: fewer than 100 events in total and confidence intervals encompass no difference as well as a clinically significant difference. ^e^One of the two studies was at high risk of bias, but judged unlikely to affect this outcome. ^f^Downgraded by two levels due to imprecision: fewer than 100 events in total.

**Summary of findings 4:** Longer compared to shorter duration of nicotine patch therapy for smoking cessation

**Longer compared to shorter duration of nicotine patch therapy for smoking cessation**						
**Patient or population:** people who smoke **Setting:** any; studies conducted in: Europe, USA **Intervention:** longer duration of nicotine patch therapy (weeks) **Comparison:** shorter duration of nicotine patch therapy (weeks)						
**Outcomes**	Anticipated absolute effects^*^ (95% CI)		**Relative effect (95% CI)**	**№ of participants (studies)**	**Certainty of the evidence (GRADE)**	**Comments**
	**Risk with shorter‐duration patch**	**Risk with longer‐duration patch**				
Smoking cessation	Study population		n/a	7078 (7 RCTs)	⊕⊕⊝⊝ Low^a,b,c^	We did not pool studies, due to substantial clinical heterogeneity in length of intervention and control patch duration, and two studies appeared in multiple comparisons. None of the individual comparisons detected a statistically or clinically significant difference between longer and shorter durations of patch therapy.
	n/a	n/a				
Overall serious adverse events	Study population		n/a	1173 (3 RCTs)	⊕⊝⊝⊝ Very low^b,d^	We did not pool studies, due to substantial clinical heterogeneity in length of intervention and control patch duration, and one study appeared in multiple comparisons. We found no significant differences in any study.
	n/a	n/a				
Treatment withdrawals	n/a		n/a	648 (2 RCTs)	⊕⊝⊝⊝ Very low^b,d^	We did not pool studies, due to substantial clinical heterogeneity in length of intervention and control patch duration. We found no significant differences in any study.
	n/a	n/a				
*The risk in the intervention group (and its 95% confidence interval) is based on the assumed risk in the comparison group and the **relative effect** of the intervention (and its 95% CI). **n/a:** not applicable; **RCT:** randomised controlled trial						
**GRADE Working Group grades of evidence** **High certainty:** we are very confident that the true effect lies close to that of the estimate of the effect. **Moderate certainty:** we are moderately confident in the effect estimate: the true effect is likely to be close to the estimate of the effect, but there is a possibility that it is substantially different. **Low certainty:** our confidence in the effect estimate is limited: the true effect may be substantially different from the estimate of the effect. **Very low certainty:** we have very little confidence in the effect estimate: the true effect is likely to be substantially different from the estimate of effect.						

^a^Downgrade by one level due to imprecision: all individual comparisons had fewer than 300 events overall. ^b^Downgrade by one level due to inconsistency: clinical heterogeneity between treatment durations in individual studies prevented pooling. ^c^Most studies were at a high risk of bias for blinding, but as studies did not detect significant effects, we think blinding was unlikely to have contributed to the outcome. ^d^Downgraded by two levels due to imprecision: fewer than 100 events overall.

**Summary of findings 5:** Fast‐acting nicotine replacement therapy compared to nicotine patch for smoking cessation

**Fast‐acting nicotine replacement therapy compared to nicotine patch for smoking cessation**						
**Patient or population:** people who smoke **Setting:** any; studies conducted in: Europe, USA **Intervention:** fast‐acting nicotine replacement therapy (NRT) **Comparison:** nicotine patch						
**Outcomes**	Anticipated absolute effects^*^ (95% CI)		**Relative effect (95% CI)**	**№ of participants (studies)**	**Certainty of the evidence (GRADE)**	**Comments**
	**Risk with nicotine patch**	**Risk with fast‐acting NRT**				
Smoking cessation	Study population		RR 0.90 (0.77 to 1.05)	3319 (8 RCTs)	⊕⊕⊕⊕ High^a^	‐
	164 per 1000	148 per 1000 (126 to 172)				
Overall serious adverse events	Study population		‐	1252 (4 RCTs)	⊕⊝⊝⊝ Very low^b,c^	Three of the four studies had no events in either arm. In the one study in which serious adverse events were reported (n = 642), the confidence interval was wide (RR 1.75, 95% CI 0.52 to 5.92).
	See comment	See comment				
Treatment withdrawals	Study population		RR 4.23 (1.54 to 11.63)	1482 (3 RCTs)	⊕⊝⊝⊝ Very low^b,d^	‐
	5 per 1000	23 per 1000 (8 to 63)				
*The risk in the intervention group (and its 95% confidence interval) is based on the assumed risk in the comparison group and the **relative effect** of the intervention (and its 95% CI). **CI:** confidence interval; **NRT:** nicotine replacement therapy; **RCT:** randomised controlled trial; **RR:** risk ratio						
**GRADE Working Group grades of evidence** **High certainty:** we are very confident that the true effect lies close to that of the estimate of the effect. **Moderate certainty:** we are moderately confident in the effect estimate: the true effect is likely to be close to the estimate of the effect, but there is a possibility that it is substantially different. **Low certainty:** our confidence in the effect estimate is limited: the true effect may be substantially different from the estimate of the effect. **Very low certainty:** we have very little confidence in the effect estimate: the true effect is likely to be substantially different from the estimate of effect.						

^a^We rated most studies at low or unclear risk of bias. However, we did not downgrade the certainty of the evidence, as limiting the analysis only to studies we judged to be at low risk of bias resulted in a consistent effect estimate and 95% confidence interval. ^b^Downgraded by two levels due to imprecision: fewer than 100 events overall. ^c^Downgraded by one level due to risk of bias: two of the four studies were at high risk of bias. ^d^Downgraded by one level due to risk of bias: two of the three studies were at high risk of bias.

**Summary of findings 6:** Comparing types of fast‐acting nicotine replacement therapy for smoking cessation

Comparing types of fast‐acting nicotine replacement therapy (NRT) for smoking cessation						
**Patient or population:** people who smoke **Setting:** any; study conducted in: South Africa **Intervention:** fast‐acting NRT (e.g. gum, lozenge, nasal spray) **Comparison:** fast‐acting NRT (e.g. gum, lozenge, nasal spray)						
**Outcomes**	Anticipated absolute effects^*^ (95% CI)		**Relative effect (95% CI)**	**№ of participants (studies)**	**Certainty of the evidence (GRADE)**	**Comments**
	**Risk with fast‐acting NRT 1**	**Risk with fast‐acting NRT 2**				
Smoking cessation ‐ oral spray versus gum	Study population		RR 0.80 (0.29 to 2.19)	75 (1 RCT)	⊕⊝⊝⊝ Very low^a,b^	‐
	200 per 1000	160 per 1000 (58 to 438)				
Smoking cessation ‐ oral spray versus inhaler	Study population		RR 2.00 (0.46 to 8.73)	75 (1 RCT)	⊕⊝⊝⊝ Very low^a,b^	‐
	80 per 1000	160 per 1000 (37 to 698)				
Smoking cessation ‐ gum versus inhaler	Study population		RR 2.50 (0.53 to 11.70)	50 (1 RCT)	⊕⊝⊝⊝ Very low^a,b^	‐
	80 per 1000	200 per 1000 (42 to 936)				
Overall serious adverse events	Study population		n/a	0 (0 RCTs)	n/a	None of our included studies reported usable data on this outcome.
	n/a	n/a				
Treatment withdrawals	Study population		n/a	0 (0 RCTs)	n/a	None of our included studies reported usable data on this outcome.
	n/a	n/a				
*The risk in the intervention group (and its 95% confidence interval) is based on the assumed risk in the comparison group and the **relative effect** of the intervention (and its 95% CI). **CI:** confidence interval; **n/a:** not applicable; **NRT:** nicotine replacement therapy; **RCT:** randomised controlled trial; **RR:** risk ratio						
**GRADE Working Group grades of evidence** **High certainty:** we are very confident that the true effect lies close to that of the estimate of the effect. **Moderate certainty:** we are moderately confident in the effect estimate: the true effect is likely to be close to the estimate of the effect, but there is a possibility that it is substantially different. **Low certainty:** our confidence in the effect estimate is limited: the true effect may be substantially different from the estimate of the effect. **Very low certainty:** we have very little confidence in the effect estimate: the true effect is likely to be substantially different from the estimate of effect.						

^a^Downgraded by one level due to risk of bias: we judged the one included study to be at high risk of bias. ^b^Downgraded by two levels due to imprecision: fewer than 100 events overall.

**Summary of findings 7:** Preloading nicotine replacement therapy (NRT) compared to standard‐use NRT for smoking cessation

**Preloading nicotine replacement therapy (NRT) compared to standard‐use NRT for smoking cessation**						
**Patient or population:** people who smoke **Setting:** any; studies conducted in: Australasia, Europe, South Africa, USA **Intervention:** preloading NRT **Comparison:** standard‐use NRT						
**Outcomes**	Anticipated absolute effects^*^ (95% CI)		**Relative effect (95% CI)**	**№ of participants (studies)**	**Certainty of the evidence (GRADE)**	**Comments**
	**Risk with standard‐use NRT**	**Risk with preloading NRT**				
Smoking cessation	Study population		RR 1.25 (1.08 to 1.44)	4395 (9 RCTs)	⊕⊕⊕⊝ Moderate^a^	‐
	136 per 1000	170 per 1000 (147 to 196)				
Overall serious adverse events	Study population		RR 1.11 (0.59 to 2.09)	3908 (4 RCTs)	⊕⊕⊝⊝ Low^b,c^	‐
	10 per 1000	11 per 1000 (6 to 21)				
Treatment withdrawals	Study population		RR 0.33 (0.01 to 7.95)	80 (1 RCT)	⊕⊝⊝⊝ Very low^d,e^	‐
	25 per 1000	8 per 1000 (0 to 199)				
*The risk in the intervention group (and its 95% confidence interval) is based on the assumed risk in the comparison group and the **relative effect** of the intervention (and its 95% CI). **CI:** confidence interval; **NRT:** nicotine replacement therapy; **RCT:** randomised controlled trial; **RR:** risk ratio						
**GRADE Working Group grades of evidence** **High certainty:** we are very confident that the true effect lies close to that of the estimate of the effect. **Moderate certainty:** we are moderately confident in the effect estimate: the true effect is likely to be close to the estimate of the effect, but there is a possibility that it is substantially different. **Low certainty:** our confidence in the effect estimate is limited: the true effect may be substantially different from the estimate of the effect. **Very low certainty:** we have very little confidence in the effect estimate: the true effect is likely to be substantially different from the estimate of effect.						

^a^Downgraded by one level due to a combination of risk of bias and imprecision: we judged five of nine studies to be at high risk of bias; removing these studies from the analysis resulted in a wider confidence interval, rendering the result no longer statistically significant (the point estimate was lower but still favoured the intervention (RR 1.16)). We rated the one included study which detected a statistically significant benefit in favour of the intervention to be at high risk of bias. ^b^Downgraded by one level due to risk of bias: we judged three of four studies to be at high risk of bias. ^c^Downgraded by one level due to imprecision: fewer than 300 events overall. ^d^Downgraded by one level due to risk of bias: we judged the one study to be at high risk of bias. ^e^Downgraded by two levels due to imprecision: fewer than 100 events overall.

## Background

### Description of the condition

Tobacco use is one of the leading causes of preventable illness and death worldwide, killing over eight million people every year (WHO 2022). Most people who smoke want to stop (CDC 2017); however, quitting tobacco use is difficult. This is due to an interplay of psychological, physiological, environmental and other factors that lead to dependence on smoking. The physiological dependence is caused by a chemical found in tobacco called nicotine (Benowitz 2010; McNeill 2017).

### Description of the intervention

Nicotine replacement therapy (NRT) is a medication formulated in a variety of ways for absorption through the oral mucosa (chewing gum, lozenges, sublingual tablets, inhaler/inhalator), nasal mucosa (spray) or skin (transdermal patches). Nicotine patches are worn on the body and deliver a nicotine dose slowly and passively through the skin. They do not replace any of the behavioural aspects of smoking. In contrast, the other types of NRT mimic some of the hand‐to‐mouth actions of smoking, provide an oral substitute, or do both, and are faster‐acting but require more effort on the user's part. Transdermal patches are available in several different doses. They deliver between 5 mg to 52.5 mg of nicotine over 24 hours, resulting in plasma levels similar to the trough levels seen between cigarettes in heavy smokers (Fiore 1992). Some brands of patch are designed to be worn for 24 hours, whilst others are to be worn for 16 hours each day. Nicotine gum is available in both 2 mg and 4 mg strengths, and nicotine lozenges are available in 1 mg, 1.5 mg, 2 mg and 4 mg strengths. However, the amount of nicotine absorbed by the user is less than the original dose. The availability of NRT products on prescription or for over‐the‐counter purchase varies from country to country. Table 8 summarises the products currently licensed in the United Kingdom.

**1:** Nicotine replacement therapies available in the UK

**Type**	**Available doses**
Nicotine transdermal patches	Worn over 16 hours: 5 mg, 10 mg, 15 mg, 25 mg doses Worn over 24 hours: 7 mg, 14 mg, 20 mg, 21 mg, 30 mg doses^a^
Nicotine chewing gum	2 mg and 4 mg doses
Nicotine sublingual tablet	2 mg dose
Nicotine lozenge	1 mg, 1.5 mg, 2 mg and 4 mg doses
Nicotine inhalation cartridge plus mouthpiece	Cartridge containing 10 mg
Nicotine metered nasal spray	0.5 mg dose/spray
Nicotine oral spray	1 mg dose/spray

Information extracted from British National Formulary ^a^35 mg/24‐hour and 53.5 mg/24‐hour patches available in other regions

### How the intervention might work

The aim of NRT is to replace the nicotine that the person who smoked tobacco would have been receiving from inhaling the tobacco smoke, without the harmful elements of tobacco smoke (McNeill 2017). This should reduce the motivation to smoke and the physiological and psychomotor withdrawal symptoms often experienced when smoking is ceased, thereby increasing the likelihood of remaining abstinent (West 2001). Nicotine undergoes first‐pass metabolism in the liver, reducing the overall bioavailability of ingested nicotine. A pill that could reliably produce high enough nicotine levels in the central nervous system would risk causing adverse gastrointestinal effects. This is why NRT was formulated for absorption through the skin or oral/nasal mucosa.

Cigarette smoking delivers nicotine rapidly, allowing nicotine to act on the brain within seconds (Benowitz 2010). None of the available NRT products deliver such high doses of nicotine as efficiently as cigarettes. The average cigarette delivers between 1 mg and 3 mg of nicotine. A person who smokes one pack a day absorbs 20 mg to 40 mg of nicotine daily (Henningfield 2005). However, despite NRT's relatively slower and lower nicotine delivery, there is high‐certainty, well‐accepted evidence that NRT helps some people to stop smoking. A Cochrane Review comparing any NRT product to control for smoking cessation found a risk ratio (RR) of 1.55 (95% confidence interval (CI) 1.49 to 1.61; 133 studies, 64,640 participants; high‐certainty evidence), suggesting that the chances of quitting were increased by 49% to 61% compared to using no NRT or placebo (Hartmann‐Boyce 2018). In addition, many clinical guidelines recommend NRT as a first‐line treatment for people seeking pharmacological help to stop smoking (Fiore 2008; Italy ISS 2004; Le Foll 2005; NICE 2022; NZ MoH 2021; Patnode 2021; US Preventive Services Task Force 2021; West 2000; Woolacott 2002; Zwar 2011).

### Why it is important to do this review

The aforementioned Cochrane Review comparing NRT to control was first published in 1996 and has been regularly updated since (Hartmann‐Boyce 2018). Despite the number of included studies more than doubling since its initial publication, the main effect estimate remained stable. The 2018 review update was therefore intended to be the final update of the evidence comparing NRT to placebo or to no pharmacotherapy, as confidence in this effect estimate is high and unlikely to be changed by further research.

However, many questions about NRT have not been answered. Evidence comparing different forms, deliveries, doses, durations and schedules of NRT is still needed, to see whether the effectiveness of NRT differs when used in different ways, and, therefore, whether approaches to NRT use can be tailored to maximise success in achieving long‐term abstinence. These factors are now evaluated separately in this Cochrane Review update. This is the first update of this Cochrane Review, first published in 2019 (Lindson 2019). We carried out this update as part of a wider project to update and synthesise all evidence on licenced pharmacotherapies and electronic cigarettes for smoking cessation (Lindson 2022).

Separate Cochrane Reviews compare NRT to other pharmacotherapies (Livingstone‐Banks 2023; Hajizadeh 2023; Lindson 2022); test the efficacy of NRT in special populations – including pregnant women (Clair 2020) and adolescents (Fanshawe 2017) – where we may reasonably hypothesise that its effectiveness differs from that in the general population; and test the effectiveness and safety of electronic cigarettes containing nicotine, which we do not include in this review, but could be considered a form of NRT (Hartmann‐Boyce 2022).

## Objectives

To determine the effectiveness and safety of different forms, deliveries, doses, durations and schedules of nicotine replacement therapy (NRT), for achieving long‐term smoking cessation.

## Methods

### Criteria for considering studies for this review

#### Types of studies

We included randomised controlled trials (RCTs), including cluster‐randomised trials and quasi‐randomised trials (i.e. trials where treatment allocation was not truly random). Cross‐over RCTs were not eligible for inclusion as this design does not allow for assessment of longer‐term intervention effects on smoking cessation.

#### Types of participants

We included people of any age who smoked and were motivated to quit, irrespective of the setting from which they were recruited or their initial level of nicotine dependence. We included studies that randomised therapists, rather than people who smoked, provided that the specific aim of the study was to examine the effect of different types of NRT use on smoking cessation. We have not included trials that randomised physicians or other therapists to receive an educational intervention, which included encouraging their patients to use NRT, but have reviewed them separately (Carson 2012).

#### Types of interventions

We included any form, dose, duration and schedule of NRT use (this could include any type of NRT, i.e. gum, transdermal patches, nasal and oral spray, inhalers and tablets or lozenges). Eligible comparisons were any other form(s), dose(s), duration(s) or schedule(s) of NRT use (this could also include any type of NRT).

The terms 'inhaler' and 'inhalator' (an oral device that delivers nicotine through the mouth by inhalation, for absorption through the buccal mucosa) are used interchangeably in the literature. We have used the term 'inhaler' throughout the rest of this review.

Studies were not eligible for inclusion if one of the study arms received an additional intervention component that could not be separated from the NRT intervention, making it impossible to establish whether any effect found was a result of the difference in NRT use or the additional component. We did not include studies that evaluated the effect of NRT for individuals who were attempting to reduce the number of cigarettes smoked rather than quit. A separate review of harm reduction approaches covers this type of study (Lindson‐Hawley 2016).

#### Types of outcome measures

##### Primary outcomes

1) Smoking cessation. This review evaluates the effects of different NRT regimens on smoking cessation. We therefore excluded trials that did not assess smoking cessation as an outcome, and also those that followed participants for fewer than six months, in line with the standard methods of the Cochrane Tobacco Addiction Group. For each study, we chose the strictest available criteria to define abstinence. For example, in studies where biochemical validation of cessation was available, only those participants who met the criteria for biochemically‐confirmed abstinence were regarded as being abstinent. Wherever possible, we chose a measure of sustained cessation rather than point prevalence. We regard people who were lost to follow‐up as being continuing smokers (West 2005).  2) Adverse events (AEs) and serious adverse events (SAEs). Number of participants reporting cardiac AEs (as defined by study authors, but including: fast or irregular heartbeat, chest pain, myocardial infarction or stroke), any SAEs, and withdrawing due to effects of the treatment where they were reported. We report cardiac AEs rather than AEs in general, as NRT is generally deemed to be safe, but cardiac AEs have been identified as a particular area of concern (Hartmann‐Boyce 2018). We did not exclude studies if they did not report AEs.

### Search methods for identification of studies

#### Electronic searches

We searched the specialised register of the Cochrane Tobacco Addiction Group (via Cochrane Register of Studies (CRS)‐Web) on 29 April 2022 for any reports of trials referring to the use of NRT of any type by searching for 'NRT', or 'nicotine' near terms for nicotine replacement products in the title, abstract or keywords. The most recent issues of the databases included in the register as searched for the current update of this review were:

- Cochrane Central Register of Controlled trials (CENTRAL; 2022, Issue 3);
- MEDLINE (via Ovid) to update 20220405;
- Embase (via Ovid) to week 202214;
- PsycINFO (via Ovid) to update 20220404.

The search strategy for the Register is given in Appendix 1. Searches for the Register are not restricted by date, language or format of publication. The Cochrane Tobacco Addiction Group's website provides details on the searches used to create the specialised register (see: tobacco.cochrane.org/resources/cochrane-tag-specialised-register). The trials register also includes trials identified by handsearching abstract books from meetings of the Society for Research on Nicotine and Tobacco.

For previous versions of the original review, we searched additional databases: CancerLit, Health Planning and Administration, Social SciSearch, Smoking & Health and Dissertation Abstracts. Since the searches did not produce additional trials, we did not search these databases after December 1996.

#### Searching other resources

Our searches of the Cochrane Tobacco Addiction Specialised Register also covered records in ClinicalTrials.gov (clinicaltrials.gov) and the World Health Organization International Clinical Trials Registry Platform (ICTRP), as these are indexed in CENTRAL. During preparation of the first version of the original review (Silagy 1996), we also sent letters to manufacturers of NRT preparations. Since this did not result in additional data, we have not repeated the exercise for subsequent updates.

### Data collection and analysis

#### Selection of studies

In previous versions of the original review (Silagy 1996; Silagy 2001; Silagy 2002; Silagy 2004; Stead 2008), one review author screened records retrieved by searches, to exclude papers that were not reports of potentially relevant studies. For the last three updates (Stead 2012, Lindson 2019, and this version), two people independently screened references to establish eligibility. We screened references in two stages. First, two review authors (for this update: AT, NL, SCC, JLB, AH) screened titles and abstracts for eligibility. For those that appeared to be eligible or where eligibility was still unclear, we retrieved full‐text papers. Two review authors (for this update: AT, NL, AH, JLB) then went on to independently screen each report for eligibility. Where there were disagreements on eligibility between the two review authors, a third review author was asked to screen the studies. We did not exclude studies based on the language of publication.

We list reports that linked to potentially relevant studies but did not report the outcomes of interest along with the main study report in the 'References to studies' section. The primary reference to the study is indicated, and for most studies, we use the first author and year as the study identifier, which corresponds to the primary reference.

#### Data extraction and management

Two people (from: AT, SCC, WY, AR) independently extracted data from the published reports and abstracts. We resolved disagreements by discussion or referral to a third review author (NL). We made no attempt to blind these individuals either to the results of the primary studies or to which treatment participants received. We examined non‐English language reports with the assistance of translators.

We extracted the following data from each study where available.

- Study characteristics: references, study registration details, country, funder, author conflicts of interest, design, including unit of randomisation.
- Recruitment methods: setting, eligibility criteria.
- Participant characteristics: number randomised, gender, baseline measures, such as cigarettes per day, any measure of levels of dependence (such as the Fagerström Test for Cigarette Dependence (FTCD; Fagerström 2012)).
- Intervention and comparator details: type of NRT, dosage, schedule of use, other details on methods.
- Common behavioural support/intervention: mode of delivery, number of sessions, length of support sessions, any other available information.
- Smoking abstinence outcome: definition of abstinence used, whether biochemical validation took place and how this was defined, number abstinent in each arm, number randomised to each arm, attrition rates.
- AE/SAE outcome: whether AEs/SAEs were measured, when they were measured, number of participants reporting a cardiac AE in each arm, number of participants reporting a serious AE in each arm, number of withdrawals in each arm due to allocated treatment.
- Risk of bias: information related to any of the risk of bias domains outlined below; information related to any other potential biases identified.

#### Assessment of risk of bias in included studies

We assessed included studies for risks of selection bias (methods of randomised sequence generation and allocation concealment), performance and detection bias (the presence or absence of blinding), attrition bias (levels and reporting of loss to follow‐up), and any other threats to study quality, using the Cochrane risk of bias tool. For each new study in this update, two review authors (from: AT, SCC, WY) independently assessed each study for each domain, in accordance with risk of bias guidance developed by the Cochrane Tobacco Addiction Group to assess smoking cessation studies. Where there was any disagreement on the assessment, a third review author (NL) acted as arbiter.

#### Measures of treatment effect

##### Smoking cessation

We extracted smoking cessation rates in the intervention and control groups from the reports at six or 12 months. Since not all studies reported cessation rates at exactly these intervals, we allowed a window of six weeks at each follow‐up point. For trials without 12‐month follow‐up, we used six‐month data. For trials that also reported follow‐up at more than a year, we used 12‐month outcomes in most cases (we note the length of follow‐up for each study in the Characteristics of included studies table). Where both validated and self‐reported quit rates were reported, we used the validated rates to calculate the study treatment effect. However, where only self‐reported data were available, we used these to calculate the treatment effect.

##### Adverse events and serious adverse events

We extracted information on whether AEs were measured, at what time points they were measured, the number of participants reporting a cardiac AE in each arm, the number of participants reporting an SAE in each arm (using the definitions provided by study authors), and the number of withdrawals in each arm due to allocated treatment.

Following the Cochrane Tobacco Addiction Group's recommended method of data analysis for dichotomous outcomes, we used the risk ratio (RR) to summarise all the individual trial outcomes where this was possible. Whilst there are circumstances in which odds ratios (ORs) may be preferable, there is a danger that they will be interpreted as if they are RRs, making the treatment effect seem larger (Deeks 2017).

#### Unit of analysis issues

We planned to include any studies that randomised participants in clusters (i.e. cluster‐RCTs), as well as those that individually randomised participants. However, none of our included studies were cluster‐randomised. A number of studies appear in multiple subgroup analyses. The reasons for this and how the analyses were subsequently managed are outlined in the forest plot footnotes: (1) not pooling the meta‐analysis; (2) splitting the number of participants in certain study arms to avoid double‐counting when pooling subgroups.

#### Dealing with missing data

We treated participants who dropped out or who were lost to follow‐up after randomisation as being continuing smokers. We note losses to follow‐up in the risk of bias table, and whether there was high or differential loss to follow‐up. The assumption that 'missing = smoking' gives conservative absolute quit rates, and will make little difference to the RR unless dropout rates differ substantially between groups.

#### Assessment of heterogeneity

We assessed clinical and methodological heterogeneity, to establish how studies should be grouped and where it was appropriate to pool studies. To assess heterogeneity statistically, we used the I^2^ statistic, given by the formula [(Q ‐ df)/Q] x 100%, where Q is the Chi^2^ statistic and df is its degrees of freedom (Higgins 2003). This describes the percentage of the variability in effect estimates that is due to heterogeneity rather than to sampling error (chance). A value greater than 50% may be considered to indicate substantial heterogeneity.

#### Assessment of reporting biases

Reporting bias is best assessed using funnel plots, where 10 or more RCTs contribute to an outcome (Higgins 2011). Therefore, where a meta‐analysis included 10 or more studies, we generated and reported on a funnel plot.

#### Data synthesis

Following assessment of clinical heterogeneity, we separated studies into the following groups testing different NRT comparisons (based on types/uses of NRT).

- Patch therapy
  - Patch dose
  - Duration of patch therapy
  - Effect of tapering patch dose
- Combination therapy
  - Combination versus single form
  - Duration of combination therapy
- Fast‐acting NRT versus patch
- Fast‐acting NRT
  - Type of fast‐acting NRT
  - Nicotine gum dose and duration
  - Fixed versus 'ad lib' dosing schedule (ad libitum or 'ad lib' means as much and as often as desired)
- NRT preloading versus standard post‐quit use
- Costs
  - Free versus purchased NRT
  - Duration of free NRT

Studies were eligible to fall within more than one comparison.

##### Smoking cessation

Within these groups, we estimated pooled weighted averages using the Mantel‐Haenszel fixed‐effect method to generate risk ratios (RRs) and 95% confidence intervals (CIs), where appropriate. We chose a priori to use a fixed‐effect method, as we assumed that there would be minimal heterogeneity in the true effect due to the nature of the intervention. Where only one study tested a comparison, we report this narratively.

##### Adverse events

Within the groups above, we conducted three analyses where the relevant data were available. We estimated a pooled weighted average using Mantel‐Haenszel fixed‐effect methods comparing the number of cardiac AEs, SAEs and withdrawals due to effects of the treatment, reported between trial arms. We generated effect estimates as the RR and 95% CI where appropriate.

#### Subgroup analysis and investigation of heterogeneity

We split the following comparisons into subgroups, to investigate whether variations between intervention characteristics resulted in varied effects.

- Patch dose: studies split according to the dosage administered; namely, 42/44 mg versus 21/22 mg and 21/25 mg versus 14/15 mg.
- Duration of patch therapy: studies split according to duration of treatment. This ranged from two weeks to 52 weeks.
- Combination versus single‐form therapy: studies split by type of combination NRT used (e.g. patch plus gum, patch plus nasal spray, etc.) and type of single NRT used (e.g. patch alone, fast‐acting NRT alone, choice of single‐form NRT, etc.).
- Duration of combination therapy: studies split according to duration of treatment. This ranged from two weeks to 16 weeks.
- Fast‐acting NRT versus patch: studies split by type of fast‐acting NRT used.
- Type of fast‐acting NRT: studies split by type of fast‐acting NRT used in either comparison group.
- 4 mg versus 2 mg nicotine gum: participants split into high‐ versus low‐dependency smokers, as defined by study authors.
- Fixed versus ad lib dosing schedule: studies split by the type of NRT used; namely, gum or nasal spray.
- NRT preloading versus standard post‐quit use: studies split by the type of NRT used (e.g. patch, gum, patch and gum).
- Free versus purchased NRT: studies split by the type of NRT used; namely, patch or gum.
- Duration of free NRT: studies split by length of period free NRT provided. This ranged from one week to eight weeks.

#### Sensitivity analysis

We carried out the following sensitivity analyses.

- We tested the impact of removing any study judged at high risk of bias for any domain on the relevant meta‐analyses.
- In Walker 2011, a very low proportion of participants who claimed to have quit completed verification (34%). We extracted actual verified rates and used these in our main analysis. We conducted a sensitivity analysis comparing these figures to data extrapolated from these proportions to the wider trial population and non‐verified rates.
- We tested, post hoc, the impact of removing studies focussed on specific populations that may be considered vulnerable (e.g. adolescents, people with alcohol use disorder, people with psychiatric disorders).

#### Summary of findings and assessment of the certainty of the evidence

Following standard Cochrane methodology, we created summary of findings tables for the following comparisons, which we deemed to be most clinically relevant:

- combination versus single‐form NRT;
- duration of combination therapy;
- patch dose;
- duration of patch therapy;
- fast‐acting NRT versus patch;
- type of fast‐acting NRT;
- NRT preloading versus standard post‐quit use.

Also, following standard Cochrane methodology (Higgins 2011; Higgins 2022), we used the five GRADE considerations (study limitations, consistency of effect, imprecision, indirectness and publication bias) to assess the certainty of the body of evidence for smoking cessation, SAEs and treatment withdrawals, and to draw conclusions about the certainty of the evidence within the text of the review.

## Results

### Description of studies

#### Results of the search

The most recent search for this update yielded 867 records for screening. After we removed 62 duplicate records, 805 records remained for title and abstract screening. We excluded 709 records at this stage, leaving 96 for full‐text screening. We identified five new studies for inclusion, two of which had been previously excluded due to lack of information (Berlin 2011; Garvey 2006), but were deemed eligible upon reassessment in this update. Alongside these five new included studies, we found four new ongoing studies (Characteristics of ongoing studies). We excluded 72 records at the full‐text screening stage. See Figure 1 for study flow information relating to the most recent update search.

**1:**
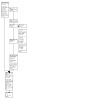
PRISMA flow diagram for the April 2022 search update *Some studies have multiple references

#### Included studies

This review update includes a total of 68 studies (159 references) involving 43,327 participants; five studies are new to this update (Berlin 2011; Dignan 2019; Garvey 2006; LeBlanc 2017; Leung 2019), whilst the remaining 63 were included in the previous review (Lindson 2019). Studies were conducted in the USA (41 studies), Europe (15 studies), Australasia (four studies), Canada (two studies), China (two studies), South Africa (two studies), South America (one study) and across multiple continents (one study). The sample size per study ranged from 45 to 3575 participants, with a median of 401. See Characteristics of included studies for further details.

##### Participants

Participants were typically adults who smoke, with an average age of approximately 44. Seven studies targeted specific populations:

- Moolchan 2005 recruited adolescents;
- Hall 2009 recruited participants over 50 years of age;
- Kornitzer 1987 recruited only men in a workplace setting;
- Cooney 2009 recruited participants who were alcohol‐dependent at the time of the study;
- Kalman 2006 recruited people with a history of alcohol dependence;
- Dennis 2016 recruited adults who smoked diagnosed with post‐traumatic stress disorder (PTSD);
- Berlin 2011 recruited people who smoked with "...either a known smoking‐related disorder or an underlying disease with increased risk for smoking‐related illnesses." 

The average number of cigarettes smoked was greater than or equal to 20 per day in most studies (48 of the 61 trials (79%)). Killen 1999 recruited only people smoking 25 or more cigarettes a day, and Hughes 1999 recruited only people smoking 30 or more a day. Seven studies did not report participants' average number of cigarettes per day.

Thirty‐two studies recruited participants directly from the community, making it the most common source of recruitment. Most participants volunteered in response to media advertisements, with one study using advertisements on internet sites (Hughes 2018). A number of studies recruited participants through referrals from clinicians or healthcare clinics, such as smoking cessation clinics or quit‐lines, substance abuse clinics, or primary care clinics, and one study recruited from referrals to a lung health clinic (Tønnesen 2000). Two studies recruited participants from previous smoking‐cessation studies (Baker 2016; Tønnesen 1996), two from worksites (Kornitzer 1987; Kornitzer 1995), and one from universities (Schnoll 2015). Some studies used a mixture of these approaches or did not report how participants were recruited.

##### Types and uses of nicotine replacement therapy

Studies addressed a range of questions relating to the effectiveness of different types and uses of NRT. The variations on NRT use tested are listed below (some studies tested more than one NRT variant):

- Patch dose (10 studies): three studies compared 25 mg to 15 mg (16‐hour) patches (CEASE 1999; Killen 1999; Paoletti 1996); one study compared 21 mg to 14 mg (24‐hour) patches (TNSG 1991); two studies compared 42 mg and 21 mg (24‐hour) patches (Kalman 2006; Rose 2010); and one study compared 44 mg to 22 mg (24‐hour) patches (Jorenby 1995). Dale 1995 and Hughes 1999 both compared three different doses: 44 mg versus 22 mg versus 11 mg (24‐hour), and 42 mg versus 35 mg versus 21 mg (24‐hour), respectively. Garvey 2006 randomised people to five nicotine patch treatment conditions: placebo, 21 mg, 42 mg, or a tailored dose at either 50% or 100% nicotine replacement based on smoking history.
- 24‐hours‐a‐day versus 16‐hours‐a‐day patch use (one study): one study included a direct comparison between groups wearing the same nicotine patches (dose and delivery system not specified) over either 16 hours (removing the patch at bedtime) or 24 hours (continuous use, including overnight) (Daughton 1991). All participants used patches for four weeks after the quit day.
- Duration of patch therapy (seven studies): Schnoll 2015 compared 52‐week use of nicotine patches to 24‐week use and 8‐week use. CEASE 1999 compared 28‐week with 12‐week use, and Schnoll 2010a compared 24‐week with 8‐week use. Bolin 1999 and Hilleman 1994 both compared 12‐week patch use to shorter patch use, i.e. six weeks and three weeks, respectively. Cummings 2011 compared 6‐, 4‐ and 2‐week use and Glavas 2003 compared 6‐week and 3‐week patch use.
- Effect of tapering patch dose (two studies): these studies compared the effect of stopping patch use abruptly at a high dose to gradually reducing patch dose over a prolonged time. Hilleman 1994 did this by providing one group of participants with 21 mg patches for six weeks and providing another group of participants with 21 mg patches for four weeks, then 14 mg patches for four weeks, then 7 mg patches for another four weeks. Stapleton 1995 gave all participants a 15 mg patch for one week; then participants could choose to receive either a continued 15 mg dose or a higher 35 mg dose for a further 11 weeks. Participants were randomised within these self‐selected groups to either taper their patch dose after the 12 weeks or to receive tapered placebo patches. Participants in the active patch group, therefore, received a further two‐week dose of 15 mg patches, followed by two weeks of 10 mg patches, followed by two weeks of 5 mg patches. The placebo group received the equivalent placebo patches.
- Combination versus single form (16 studies): combination NRT usually describes using nicotine patches and a fast‐acting form of NRT, such as gum or lozenge. Cooney 2009, Kornitzer 1995, Leung 2019, Puska 1995 and Smith 2013 all studied patch in combination with nicotine gum. Puska 1995 compared combination therapy to gum alone, whereas the other studies compared combination therapy to patch alone. Blondal 1999 and Croghan 2003 combined patch with nasal spray. Blondal 1999 used patch alone as the comparator, whereas Croghan 2003 had a group of participants that received patch alone and a group that received nasal spray alone. Bohadana 2000, Caldwell 2016 and Tønnesen 2000 combined patches with inhaler; Caldwell 2016 compared to patch alone, Bohadana 2000 to inhaler alone, and Tønnesen 2000 compared to both patch alone and inhaler alone. Baker 2016, Krupski 2016, Piper 2009 and Smith 2009 all used patch in combination with lozenge. Baker 2016 and Krupski 2016 compared combination NRT to patch alone, whereas both Piper 2009 and Smith 2009 compared combination NRT to a group receiving patch only and a group receiving lozenge only. Caldwell 2014 combined patch with oral spray and compared this to patch use alone. Finally, Dignan 2019 (incomplete factorial design) compared the choice of one NRT product with choice of two NRT products (patch or gum or lozenge); we assumed that patch was likely selected when used in combination with a choice of fast‐acting NRT (gum or lozenge), as this is in‐line with common practice.
- Duration of combination therapy (three studies): these studies investigated the optimum length of combination patch plus gum use. Smith 2013 compared 6‐week to 2‐week use, Piper 2016 compared 16‐week to 8‐week use, and Schlam 2016 compared 26‐week to 8‐week use.
- Fast‐acting NRT versus patch (eight studies): fast‐acting NRT refers to the faster‐acting (non‐patch) formulations of NRT, such as gum, lozenge, nasal spray, and so on. One study compared patch to inhaler (Tønnesen 2000), two studies compared patch to nasal spray (Croghan 2003; Lerman 2004), three studies compared patch to lozenge (Piper 2009; Schnoll 2010b; Smith 2009), and two studies compared patch to gum (Kupecz 1996; Moolchan 2005).
- Type of fast‐acting NRT (one study): only Bolliger 2007 compared the effectiveness of different forms of fast‐acting NRT by comparing oral spray to gum to inhaler.
- Nicotine gum dose (five studies): these studies compared 4 mg nicotine gum to 2 mg nicotine gum (Garvey 2000; Herrera 1995; Hughes 1990; Kornitzer 1987; Tønnesen 1988).
- Duration of gum use (one study): Hall 2009 investigated whether the duration of gum use affected quit rates. The intervention group used gum for 50 weeks and the comparison group used gum for 10 weeks.
- Fixed versus ad lib dosing schedule (four studies): these studies investigated whether instructions on when to use fast‐acting NRT influenced effectiveness. Goldstein 1989 and Killen 1990 provided participants with 2 mg nicotine gum; Rey 2009 and Tønnesen 1996 provided participants with nasal spray. The fixed‐dosing groups were either asked to use one piece/puff per hour (Goldstein 1989; Killen 1990; Tønnesen 1996), or two puffs per hour (Rey 2009), regardless of cravings. The ad lib dosing groups were all asked to use their product when a craving occurred, with a maximum upper limit for daily use, i.e. 30 pieces of gum a day or 80 puffs of nasal spray.
- NRT preloading versus standard post‐quit NRT use (nine studies): traditionally, NRT is used from a quit date onward, after tobacco use has ceased. NRT preloading is when NRT is used before the quit day, whilst the participant is still smoking. Seven studies provided participants with nicotine patches pre‐quit day (Dennis 2016; Preloading Investigators 2018; Rose 1994; Rose 1998; Rose 2006; Rose 2009; Schuurmans 2004), and two studies included participants that used patch alone, gum alone and patch plus gum pre‐quit day (Bullen 2010; Piper 2016). The length of nicotine preloading also varied across studies. Seven studies initiated NRT use two weeks before the quit date (Bullen 2010; Dennis 2016; Rose 1994; Rose 1998; Rose 2006; Rose 2009; Schuurmans 2004), one initiated use three weeks before the quit date (Piper 2016), and one initiated use four weeks before the quit date (Preloading Investigators 2018). Following the quit date, all study arms received active NRT.
- Stopping patch use versus continuing patch use on relapsing (one study): Hughes 2018 tested whether the instruction to stop using a nicotine patch in the event of a smoking lapse resulted in different quit rates to the instruction to continue using a patch in the event of a lapse, in participants who were using nicotine patches after a quit day.
- Free versus purchased NRT (two studies): these studies investigated whether buying NRT versus being provided with NRT free of charge resulted in different quit rates. Hughes 1991 had three study arms that all used nicotine gum. Participants were randomised to: 1) a free prescription for six months; 2) buying the gum for USD 6 per box; 3) buying the gum for USD 20 per box. Hays 1999 also randomised participants to three groups: 1) nicotine patches provided free of charge; 2) placebo patches provided free of charge; 3) nicotine patches bought by participants. The placebo patch group is excluded from this review.
- Duration of free NRT (two studies): these studies provided participants with NRT free of charge for a limited period of the study, then encouraged participants to source the remainder of the treatment themselves. The length of free NRT varied between trial arms. Abdullah 2013 provided two weeks of free patch or gum (depending on participant preference) in one arm and one week free in the other arm. In both arms, participants were encouraged to use NRT for a total of eight to 12 weeks, sourcing the remainder themselves. Burns 2016 provided participants with eight weeks of nicotine patches in one arm and four weeks in another arm. Participants were encouraged to use patches for a total of 10 weeks and to source the remainder themselves.

In addition to the comparisons above, Walker 2011 provided participants with a 1‐week free NRT selection box (including one patch, gum, inhaler, sublingual tablets and oral pouches), followed by eight weeks of free participant‐selected NRT in the intervention arm. The comparison arm received eight weeks of subsidised NRT patches or gum. Tulloch 2016 provided one group of participants with nicotine patches for 10 weeks, beginning on the quit day. Participants were provided with a maximum dose of 21 mg or 14 mg, depending on their baseline cigarettes per day. Dosage was then tapered from weeks seven to 10. Another group of participants self‐titrated their nicotine patch dosage to a maximum of 35 mg, and also used ad libitum nicotine gum or inhaler for up to 22 weeks. LeBlanc 2017 compared a control group receiving 10 weeks of declining, standard dose (not specified) nicotine patch to 10 weeks of nicotine patch, titrated based on smoking history combined with a nicotine inhaler, used ad libitum. Berlin 2011 provided one group of participants with a nicotine dose aimed at substituting 100% (± 5%) of their nicotine prescribed based on the previous week's saliva cotinine concentrations. This group was compared to standard care in which participants received nicotine doses mixed based on dependence. Nicotine doses were delivered via nicotine patch, in addition to gum or lozenge, at the investigators' discretion. 

#### Excluded studies

We listed the reasons for excluding 51 studies (63 references) that were potentially relevant in Characteristics of excluded studies. For this update, we excluded most studies at full‐text screening stage because they had an ineligible comparator; for example, placebo rather than another form of NRT. A separate Cochrane Review assesses this type of study (Hartmann‐Boyce 2018).

#### Ongoing studies

We found four ongoing studies as part of this updated search which may be relevant for inclusion when complete.

- NCT03538938: a four‐factor factorial design with 16 treatment combinations. Factors included: (1) 1‐call versus 4‐call quit‐line counselling; (2) nicotine patch versus patch plus lozenge; (3) enroled versus informed about smokefreeTXT (an 'evidence‐based smoking cessation texting support program'); (4) financial incentives versus no financial incentives for treatment engagement.
- NCT03611881: a three‐factor factorial design with eight treatment combinations. Factors included: (1) 4‐week versus 8‐week behavioural counselling; (2) 2‐week versus 8‐week nicotine patch; (3) no referral versus counsellor‐facilitated referral to a community‐based programme to address social needs.
- NCT04188873: a four‐factor factorial design with 16 treatment combinations. Factors included: (1) varenicline versus combination NRT; (2) 4‐week versus standard preparation medication; (3) 12‐week versus 24‐week medication duration; (4) minimal versus intensive counselling.
- Zawertailo 2020: will compare a daily 21 mg nicotine patch plus placebo patch to a daily 21 mg nicotine patch plus additional patch at a dose based on tolerability and number of cigarettes per day in the preceding week. Both groups will receive treatment for five weeks of titration and five weeks of maintenance, then tapering down by 7 mg/week.

Further details are summarised in Characteristics of ongoing studies.

### Risk of bias in included studies

Overall, we judged nine studies to be at low risk of bias (low risk of bias across all domains), 28 at high risk of bias (high risk of bias in at least one domain), and the remaining 31 at unclear risk of bias. A summary illustration of the risk of bias profile across trials is shown in Figure 2.

**2:**
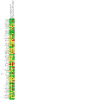
Risk of bias summary: review authors' judgements about each risk of bias item for each included study

#### Allocation

We assessed selection bias through investigating methods of random sequence generation and allocation concealment for each study. We rated 30 studies at low risk of bias for random sequence generation, 37 at unclear risk and one at high risk (Kupecz 1996). We judged Kupecz 1996 to be at high risk as it was described as 'quasi‐experimental', with month of recruitment randomised to study arm (gum or patch), and all people recruited in each month provided with the allotted treatment. We judged 28 studies to be at low risk for allocation concealment and 40 at unclear risk.

When assessing both random sequence generation and allocation concealment, an unclear risk of bias resulted from insufficient information about methods used in studies, making it impossible to be sure whether bias was present or not.

#### Blinding

We assessed any risk of bias linked to blinding as one domain. However, we took into account both performance and detection bias when making this judgement. Although we are assessing a pharmaceutical treatment (NRT) in this review, there were some circumstances where the variation in treatment between arms meant it would be impossible to blind participants and study personnel by using a placebo. For example, in Abdullah 2013, the intervention being tested was the length of time NRT was supplied to participants for free (overall length of NRT use was the same). In such cases, we did not rate studies at high risk as long as participants received similar amounts of face‐to‐face contact between groups, abstinence was biochemically verified, or both. We judged 21 studies to be at low risk of bias for this domain, 23 at unclear risk and 24 at high risk.

#### Incomplete outcome data

We judged studies to be at low risk of attrition bias where the numbers of participants lost to follow‐up were clearly reported, the overall number lost to follow‐up was not more than 50%, and the difference in loss to follow‐up between groups was no greater than 20%. This is in accordance with risk of bias guidance produced by the Cochrane Tobacco Addiction Group for assessing smoking cessation studies. We found that 39 of the studies were at low risk of bias, 22 were at unclear risk and seven were at high risk. In six of the seven studies at high risk (Caldwell 2014; Dennis 2016; Dignan 2019; Krupski 2016; Leung 2019; Rose 2009), this was because overall loss to follow‐up was more than 50%. The rating of high risk in Hughes 1999 was because the study was terminated early by the sponsor, resulting in incomplete long‐term follow‐up data; losses were included in the analysis as non‐abstinent.

### Effects of interventions

See: Table 1; Table 2; Table 3; Table 4; Table 5; Table 6; Table 7

Please see Table 1; Table 2; Table 3; Table 4; Table 5; Table 6; Table 7.

#### Patch therapy

##### Dose

See Table 3. We treated three groups of studies that compared different patch doses as separate groups for our first comparison: group 1: 42/44 mg versus 21/22 mg patches; group 2: 25 mg versus 15 mg patches; group 3: 21 mg versus 14 mg patches. Although the doses included in groups 2 and 3 appear comparable, the patches used in these groups did not have comparable delivery systems, meaning the doses delivered to participants per hour were likely to be different across the two groups. The three studies comparing the 25 mg dose to the 15 mg dose all used patches that delivered nicotine over a 16‐hour period (to be worn during waking hours) (CEASE 1999; Killen 1999; Paoletti 1996), so the doses delivered per hour were approximately 1.6 mg and 0.9 mg. However, in TNSG 1991, which compared a 21 mg dose with a 14 mg dose, the patches used delivered nicotine over 24 hours (to be worn continuously, including overnight), resulting in doses of approximately 0.9 mg and 0.6 mg per hour. The five studies comparing 42/44 mg doses with 21/22 mg doses all used patches that delivered nicotine over 24 hours (Dale 1995; Hughes 1999; Jorenby 1995; Kalman 2006; Rose 2010), so the approximate doses delivered per hour were 1.8 mg and 0.9 mg, respectively.

When we compared 21 mg to 14 mg (24‐hour) patches, we found an effect on smoking cessation in favour of the higher dose, with confidence intervals (CIs) excluding no difference (risk ratio (RR) 1.48, 95% CI 1.06 to 2.08; 1 study, 537 participants; Analysis 1.1). When we compared 25 mg to 15 mg (16‐hour) patches, the point estimate was in favour of the higher dose; however, the lower limit of the CI was one (RR 1.19, 95% CI 1.00 to 1.41; I^2^ = 0%; 3 studies, 3446 participants). Finally, when we compared 42 mg or 44 mg to 21 mg or 22 mg (24‐hour) patches, the point estimate was lower, and CIs included the possibility of no difference and of favouring the lower dose (RR 1.09, 95% CI 0.93 to 1.29; I^2^ = 38%; 5 studies, 1655 participants). Results were not sensitive to the exclusion of one study at a high risk of bias or the removal of the Kalman 2006 study, which focused on a specific population of people with alcohol use disorder (we conducted the latter sensitivity analysis post hoc).

When we compared high‐ (25 mg) and low‐dose (15 mg) 16‐hour patches, evidence was inconclusive and CIs included the possibility of higher, lower and no difference in the risk of fast or irregular heartbeat (RR 0.92, 95% CI 0.64 to 1.33; I^2^ = 0%; 2 studies, 3269 participants; Analysis 1.2) or myocardial infarctions (RR 0.50, 95% CI 0.05 to 5.51; 1 study, 2861 participants; Analysis 1.3) when the higher dose was used. Only two of nine studies reported cardiac adverse events (AEs) by trial arm (CEASE 1999; Killen 1999). Hughes 1999 reported that 8% of the 42 mg (24‐hour) patch group experienced cardiac side effects but did not report data for the other treatment arms, so could not be included in the meta‐analysis.

Three studies comparing patch doses collected data on overall serious adverse effects (SAEs); however, only two studies reported events and contributed to the pooled effect estimate (Hughes 1999; Jorenby 1995; TNSG 1991). This pooled estimate showed an increased number of events in the higher‐dose arm but with wide CIs incorporating no difference as well as potentially favouring the lower‐dose (RR 5.01, 95% CI 0.87 to 28.82; I^2^ = 0%; 3 studies, 1560 participants; Analysis 1.4). The overall number of events was notably very small (seven in the higher‐dose arms and one in the lower‐dose arms).

When we compared 42/44 mg versus 21/22 mg (24‐hour) patches, we found a difference in study withdrawals due to treatment, with more withdrawals occurring in participants receiving higher‐dose patches and CIs excluding no difference (RR 4.99, 95% CI 1.60 to 15.50; I^2^ = 0%; 2 studies, 544 participants; Analysis 1.5). However, when we compared 21 mg to 14 mg (24‐hour) patches, the evidence was inconclusive (RR 0.77, 95% CI 0.36 to 1.64; 1 study, 537 participants; Analysis 1.5). Two additional studies reported treatment withdrawals overall rather than by trial arm: CEASE 1999 reported that 2% of participants withdrew overall and Rose 2010 reported 3% overall.

A final study randomised people to five nicotine‐patch treatment conditions (placebo, 21 mg, 42 mg, or a tailored dose at either 50% or 100% nicotine replacement based on smoking history) (Garvey 2006). The authors concluded that individualising 100% replacement of nicotine based on smoking history was not more efficacious than standard patch treatment in moderately‐ to heavily‐dependent people who smoked; however, findings of other relevant comparisons were not reported. 

##### Duration

See Table 4. None of the comparisons based on duration of patch therapy showed evidence of a difference for smoking cessation (Analysis 2.1), SAEs (Analysis 2.2) or treatment withdrawal (Analysis 2.3). Studies were so clinically heterogenous that we did not pool across subgroups. For individual subgroups, the number of included studies was small, and CIs were generally wide, meaning we cannot rule out a clinically significant difference or conduct sensitivity analyses.

In terms of safety, four studies comparing different durations of patch therapy reported cardiac AEs (CEASE 1999; Glavas 2003; Schnoll 2010a; Schnoll 2015). However, meta‐analysis was not possible due to a lack of reporting of events by the duration of treatment (CEASE 1999), measuring AEs for different lengths of time by treatment arm (Glavas 2003), and not reporting AEs cumulatively across time points (Schnoll 2010a; Schnoll 2015). However, Glavas 2003 reported no cardiac AEs in either the 3‐ or 6‐week NRT groups during the time participants were on treatment. Cardiac AEs were also rare and similar between trial arms in Schnoll 2010a and Schnoll 2015 (see Appendix 2).

##### Effect of tapering

Neither of the two studies that compared the tapering of patch dose before end of treatment to abrupt withdrawal indicated any difference in effect on smoking cessation, but CIs are wide and there was imprecision around this estimate (RR 0.99, 95% CI 0.74 to 1.32; I^2^ = 0%; 2 studies, 264 participants; Analysis 3.1). Results were not sensitive to removing the one study at a high risk of bias. 

Neither of the studies reported cardiac AEs or SAEs. Hilleman 1994 found no clear evidence of a difference between tapering and abrupt withdrawal on withdrawals due to treatment (RR 0.90, 95% CI 0.35 to 2.35; 1 study, 140 participants; Analysis 3.2). Stapleton 1995 reported 2% treatment withdrawals but did not report these by trial arm and so could not be included in the meta‐analysis.

##### Other variations in patch use

Two final studies tested the effects of other variations in patch use (Daughton 1991; Hughes 2018). These studies did not fall under the headings above and we did not enter them into a meta‐analysis.

- Daughton 1991 looked at the effect of using the same nicotine patches (nicotine dose and delivery system not specified) for 24 hours a day versus 16 hours a day (in the former group, participants wore patches overnight, and in the latter, during waking hours only). Quit rates were higher in the 16‐hour a day patch use group (17/55 participants) compared to the 24‐hour group (11/51 participants), but CIs encompassed no difference as well as an effect in the opposite direction (RR 0.70, 95% CI 0.36 to 1.34; 106 participants; Analysis 13.1). Whilst Daughton 1991 reported common AEs, it did not report specifically on cardiac AEs or SAEs. Overall, 1.3% of participants withdrew due to treatment, but withdrawals by treatment arm were not reported (Appendix 2).
- Hughes 2018 found no clear evidence that instructing participants to continue using a patch in the event of a smoking lapse resulted in higher quit rates than instructing participants to stop using a nicotine patch in the event of a lapse: 174/356 quit in the continuing group and 190/345 in the stopping group (RR 0.89, 95% CI 0.77 to 1.02; 701 participants; Analysis 13.1). Hughes 2018 also found no evidence of an effect of differential NRT use on SAEs, though CIs were wide (RR 0.97, 95% CI 0.24 to 3.84; 1 study, 701 participants; Analysis 13.6).

#### Combination therapy

##### Combination versus single form

See Table 1. Pooled data from 16 studies found greater quit rates following combination NRT treatment when compared to single‐type NRT for smoking cessation (RR 1.27, 95% CI 1.17 to 1.37; I^2^ = 12%; 16 studies, 12,169 participants; Analysis 4.1). When split into subgroups, this was equally true for combination therapy compared to: (1) patch alone (RR 1.24, 95% CI 1.13 to 1.37; I^2^ = 28%; 13 studies, 9522 participants); (2) a fast‐acting form of NRT alone (RR 1.30, 95% CI 1.09 to 1.54; I^2^ = 0%; 6 studies, 2364 participants); (3) a choice of a single form of NRT (patch or fast‐acting NRT; RR 5.16, 95% CI 1.18 to 22.6; 1 study, 253 participants). There was no clear evidence of subgroup differences (I^2^= 45.8%, P = 0.16, effect consistent across groups). Results were not sensitive to removing studies at a high risk of bias.

As this meta‐analysis included over 10 studies, we generated a funnel plot to investigate the likelihood of publication bias (Figure 3). The plot does not provide evidence of publication bias, but as the number of studies included is low (16 studies), this should be interpreted with caution.

**3:**
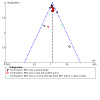
Funnel plot of comparison 4: combination versus single‐form nicotine replacement therapy, outcome: 4.1 smoking cessation

Whilst 12 of the 16 studies comparing combination NRT to single‐type NRT reported some AE data, only three studies reported cardiac AEs (Caldwell 2016; Cooney 2009; Leung 2019). Two of these studies were included in a meta‐analysis (Cooney 2009; Leung 2019). This showed wide CIs which are consistent with both a clinically significant increase and a clinically significant decrease in cardiac AEs when using combination in comparison to single‐form NRT (RR 0.66, 95% CI 0.22 to 2.05; 2 studies, 656 participants; Analysis 4.2). Interpretation was not sensitive to removing the one study at a high risk of bias (Leung 2019). Caldwell 2016 reported chest discomfort and palpitations at multiple time points but did not report these cardiac AEs cumulatively across time points and so could not be included in the meta‐analysis. However, cardiac AEs were generally similar between groups at each time point (Appendix 2).

In this comparison, SAEs were generally rare, with seven such events across the five studies that reported SAEs by treatment arm. CIs were wide and consistent with the possibility of both clinically significant harm and clinically significant benefit when comparing SAEs between combination and single‐form NRT (RR 4.44, 95% CI 0.76 to 25.85; I^2^ = 35%; 5 studies, 2888 participants; Analysis 4.3). Subgrouping by the type of single‐form NRT used (e.g. patch alone or fast‐acting NRT alone) resulted in no evidence of subgroup differences (P = 0.23, I^2^ for subgroup differences = 29.9%). Pooled subgroup effects also had wide CIs consistent with both clinically significant benefit and harm when comparing combination NRT versus patch (RR 11.45, 95% CI 0.64 to 205.90; 4 studies, 2313 participants; Analysis 4.3) and combination NRT versus fast‐acting NRT (RR 1.00, 95% CI 0.06 to 15.88; 2 studies, 575 participants; Analysis 4.3). Piper 2009 (1504 participants) reported 32 SAEs not considered related to treatment over six months but did not report these by trial arm and so could not be included in the meta‐analysis.

Five studies reported withdrawals due to treatment effects by trial arm; however, only three contributed data to the meta‐analysis as the remaining two reported no withdrawals in any of the relevant study arms. Comparing treatment withdrawals for combination NRT versus single‐form NRT, there was no evidence of a difference (RR 1.12, 95% CI 0.57 to 2.20; 5 studies, 3070 participants; Analysis 4.4). However, there was substantial heterogeneity (I^2^ = 73%). When we divided studies into subgroups, and compared combination NRT with NRT patch, the point estimate favoured combination NRT; however, CIs included the possibility of no difference (RR 2.32, 95% CI 0.99 to 5.40; I^2^ = 61%; 5 studies, 1982 participants; Analysis 4.4). The same was observed when we compared combination NRT with fast‐acting forms of NRT (RR 0.14, 95% CI 0.02 to 1.08; I^2^ = not estimable, as one of the studies had no events; 2 studies, 1088 participants; Analysis 4.4).

Our effect estimates for smoking cessation and any cardiac AE were not sensitive to the post hoc removal of the Cooney 2009 study, which focused on a specific population of people with alcohol use disorder.

##### Duration of combination therapy

See Table 2. Two of the studies testing duration of combination NRT found no evidence of a difference in effect on abstinence between shorter‐ and longer‐duration therapy (Analysis 5.1). We did not pool these studies in a meta‐analysis as they compared different durations of use. Piper 2016 compared 16‐week to 8‐week combination NRT use, with an RR of 0.96 (95% CI 0.75 to 1.23; 637 participants), and Smith 2013 compared 6‐week to 2‐week combination NRT use, with an RR of 1.11 (95% CI 0.94 to 1.31; 987 participants). Smith 2013 was a factorial trial and did not report results on duration for combination NRT only; we therefore combined study arms receiving combination NRT and gum alone, as the authors reported there was no interaction between the two groups.

We did not include Schlam 2016 in this analysis. The study had a factorial design and statistical interactions between factors were reported in the paper. We contacted study authors who supplied group‐by‐group quit rates. We checked to see if the odds ratio (OR) generated from these data resulted in a clinically different interpretation of the OR generated for the regression model adjusting for interactions in the paper, for the relevant comparison of 26‐ versus 8‐week use of combination NRT. The ORs were similar, but the wider CIs generated from the basic quit‐rate data changed the interpretation of the results. The analysis accounting for interactions in the paper resulted in an effect of 26‐week gum, with CIs excluding no difference (OR 1.40, 95% CI 1.08 to 1.82); however, the CIs did include the possibility of no difference when we used basic quit‐rate data supplied by the authors (OR 1.42, 95% CI 0.98 to 2.05; 544 participants). This suggests it would be inappropriate to use the basic quit rates to calculate RRs and 95% CIs for the duration of combination therapy comparison, ignoring the interactions detected with other intervention factors.

All three studies testing duration of combination NRT reported SAEs by trial arm (Analysis 5.2). There were no SAEs in either Piper 2016 or Smith 2013.** **Schlam 2016 reported no SAEs in the published paper but reported the occurrence of SAEs on ClinicalTrials.gov. Analysis of the number of SAEs reported in this trial registry found wide CIs and included the possibility of no difference, benefit and harm of longer‐term use (i.e. 26 weeks versus eight weeks; RR 1.63, 95% CI 0.60 to 4.42; 1 study, 544 participants; Analysis 5.2).

None of the studies reported treatment withdrawals by trial arm.

#### Fast‐acting NRT versus patch

See Table 5. None of the studies that compared a form of fast‐acting NRT to nicotine patch found an effect on smoking cessation, whether subgrouped according to type of fast‐acting NRT or combined (RR 0.90, 95% CI 0.77 to 1.05; I^2^ = 0%; 8 studies, 3319 participants). There was no evidence of a difference in effects between subgroups (P = 0.57; I^2^ = 0%; effects for individual subgroups can be found in Analysis 6.1). The overall effect was not sensitive to the removal of studies judged to be at a high risk of bias or the removal of the Moolchan 2005 study, which focused on adolescents, who may respond differently to NRT treatment. The latter sensitivity analysis was conducted post hoc.

Only one small study reported cardiac AEs by trial arm (Kupecz 1996). In this study, there were no events in either the gum or patch groups.

Three of the four studies that reported SAEs by trial arm had no SAEs (Kupecz 1996; Lerman 2004; Tønnesen 2000). Schnoll 2010b found no evidence of a difference in SAEs between lozenge and patch groups (RR 1.75, 95% CI 0.52 to 5.92; 1 study, 642 participants; Analysis 6.3). Piper 2009 reported 36 SAEs over six months, but did not report these by trial arm and so could not be included in a meta‐analysis.

When comparing withdrawals due to treatment between fast‐acting NRT and NRT patches, more participants withdrew in the fast‐acting NRT groups (RR 4.23, 95% CI 1.54 to 11.63; I^2^ = 0%; 3 studies, 1482 participants; Analysis 6.4). We also conducted subgroup analysis by type of fast‐acting NRT. When we compared nasal spray and patch, nasal spray was associated with more withdrawals (RR 3.47, 95% CI 1.15 to 10.46; 1 study, 922 participants; Analysis 6.4), with CIs excluding no difference. The direction of effect favoured greater risk of withdrawals following gum versus patch use; however, CIs were very wide (RR 11.00, 95% CI 0.63 to 191.04; 1 study, 38 participants; Analysis 6.4). There were no treatment withdrawals in either group in the study comparing lozenge with patch.

#### Fast‐acting NRT

##### Type

See Table 6. One small study of 100 participants compared smoking cessation rates across three types of fast‐acting NRT (oral spray, gum and inhaler) (Bolliger 2007). CIs were wide and encompassed the possibility of no difference for all comparisons (Analysis 7.1). Whilst this study reported some adverse event data, it did not report on cardiac AEs, SAEs or treatment withdrawals.

##### Gum dose

Five studies compared 4 mg to 2 mg gum use. Overall, 4 mg gum had a greater effect on long‐term abstinence, with CIs excluding no difference (RR 1.43, 95% CI 1.12 to 1.83; I^2^ = 63%; 5 studies, 856 participants; Analysis 8.1), but with moderate statistical heterogeneity between studies. In this group of studies, we conducted subgroup analyses to test whether effects differed between people with low‐ and high‐dependency on smoking (this was not consistently done in other groups of studies). Our post hoc subgroup analysis found that when we split studies/participants into people with lower‐dependency (Garvey 2000; Hughes 1990; Kornitzer 1987), and higher‐dependency (Garvey 2000; Herrera 1995; Kornitzer 1987; Tønnesen 1988), with Garvey 2000 and Kornitzer 1987 split across the two subgroups, this heterogeneity reduced substantially. We found a benefit of the 4 mg dose (RR 1.85, 95% CI 1.36 to 2.50; I^2^ = 13%; 4 studies, 618 participants) in people highly‐dependent on smoking, with CIs excluding no difference, and no clear evidence of an effect in people with low‐dependency (RR 0.77, 95% CI 0.49 to 1.21; I^2^ = 0%; 3 studies, 238 participants). There was evidence of a subgroup difference (P = 0.002; I^2^ = 90%). None of the studies included in this subgroup analysis were at high risk of bias; however, the findings from this analysis is limited by a low number of studies and an uneven covariate distribution.

One small study reported palpitations by trial arm (Tønnesen 1988). Palpitations were greater in 4 mg compared to 2 mg gum doses, but CIs were wide and also encompassed the opposite effect (RR 3.64, 95% CI 0.15 to 85.97; 1 study, 60 participants; Analysis 8.2). No studies comparing gum dose reported on SAEs. However, two studies reported withdrawals due to treatment by trial arm (Garvey 2000; Tønnesen 1988). There was no evidence of an effect of gum dose on treatment withdrawals (RR 1.08, 95% CI 0.18 to 6.36; I^2^ = 0%; 2 studies, 465 participants; Analysis 8.3).

##### Gum duration

Hall 2009 found no effect of 50‐week gum use over 10‐week gum use on smoking abstinence. Eighty‐five of 203 participants quit in the 50‐week duration group and 80 of 199 participants in the 10‐week duration group (RR 1.04, 95% CI 0.82 to 1.32; 402 participants; Analysis 13.1). The 50‐week group experienced a greater number of SAEs, but CIs were wide and also encompassed the opposite effect (RR 2.21, 95% CI 0.69 to 7.05; 1 study, 402 participants; Analysis 13.6). The same was found for the sensation of midsternal pressure (RR 2.94, 95% CI 0.12 to 71.77; 1 study, 402 participants; Analysis 13.2). It did not report on other cardiac AEs or treatment withdrawals.

##### Fixed versus ad lib dosing schedule

There was no clear evidence of an effect of fixed versus ad lib dosing of fast‐acting NRT on abstinence, with the CI including the possibility of no difference (RR 1.12, 95% CI 0.87 to 1.45; I^2^ = 8%; 4 studies, 828 participants; Analysis 9.1). Two of the studies tested dosing schedule using gum and two using nasal spray; however, neither group demonstrated an effect and there was no evidence of subgroup differences. Removal of one study judged to be at high risk of bias did not affect the interpretation of subgroup or overall effect estimates.

Only one small study reported cardiac AEs and SAEs (Tønnesen 1996). However, the cardiac AEs were not reported cumulatively, or by treatment arm at all time points (Appendix 2). There were no SAEs in the study.

Three studies reported withdrawals due to treatment. In Tønnesen 1996, there were no withdrawals in either the fixed‐dose or the ad lib nasal spray groups. Killen 1990 found no evidence of a difference between fixed‐dose and ad lib gum (RR 0.89, 95% CI 0.49 to 1.59; 1 study, 299 participants; Analysis 9.3). Rey 2009 reported 4% treatment withdrawals across the study, but did not report these by trial arm.

#### NRT preloading versus standard post‐quit use

See Table 7. Overall, evidence from nine studies comparing NRT use with no NRT use before a quit day, whilst concurrently smoking, found a positive effect of NRT preloading on abstinence (RR 1.25, 95% CI 1.08 to 1.44; I^2^ = 0%; 9 studies, 4395 participants; Analysis 10.1), with CIs excluding no difference.

We split participants in the included studies into three subgroups: people who used a patch only for preloading; people who used a patch plus gum; and people who used gum only (Bullen 2010 and Piper 2016 were included in all three groups, as they each had distinct groups of participants who used patch alone, gum alone, or both). The clear positive effect of preloading was only found in those participants where a patch only was used (RR 1.28, 95% CI 1.09 to 1.49; I^2^ = 0%; 9 studies, 3830 participants). Tests for subgroup differences suggested that there was very little heterogeneity between subgroup effects (P = 0.43; I^2^ = 0%), but the numbers of participants contributing to the gum alone (306 participants) and patch plus gum (259 participants) subgroups were comparatively low, resulting in wider CIs.

When we removed the five studies judged to be at high risk of bias for at least one domain from the overall analysis, the CIs for the pooled effect widened but the point estimate still favoured the intervention (RR 1.16, 95% CI 0.93 to 1.46; 4 studies, 1444 participants).

One study reported palpitations (Preloading Investigators 2018), with an increased likelihood found in the preloading arm and CIs excluding no difference (RR 2.05, 95% CI 1.15 to 3.62; 1792 participants; Analysis 10.2). One study reported cardiac AEs (Bullen 2010), with no difference detected between study arms; however, CIs were wide (RR 1.25, 95% CI 0.50 to 3.15; 1100 participants; Analysis 10.3). Three studies reported cardiac SAEs, and again demonstrated no clear evidence of a difference, with CIs incorporating the potential for both benefit and harm of the intervention, as well as no difference (RR 1.94, 95% CI 0.81 to 4.65; I^2^ = 0%; 3529 participants; Analysis 10.4). Four studies reported overall SAEs, and as with cardiac SAEs, found no clear evidence of a difference (RR 1.11, 95% CI 0.59 to 2.09; I^2^ = 0%; 3908 participants; Analysis 10.5). The one study reporting treatment withdrawals also found no evidence of a difference between NRT preloading and standard post‐quit use (Rose 1998) (RR 0.33, 95% CI 0.01 to 7.95; 80 participants; Analysis 10.6).

#### Cost of NRT

##### Free versus purchased

One study found greater quit rates in the group receiving free over purchased patches in an over‐the‐counter setting (Hays 1999); however, CIs were wide and also encompassed the possibility of lower quit rates (RR 1.24, 95% CI 0.77 to 1.99; 636 participants). Another small study investigating the cost of nicotine gum for participants receiving brief physician advice also found greater quit rates in the free gum group compared to the 'close to full price' gum group (Hughes 1991); however, CIs were wide and included the possibility of the opposite effect, as well as no difference (RR 2.70, 95% CI 0.89 to 8.20; 104 participants). This is despite the fact that people who could get free gum were much more likely to obtain it. Only Hays 1999 reported cardiac AEs, finding no clear evidence of a difference between free and purchased patches (RR 0.55, 95% CI 0.18 to 1.61; 1 study, 636 participants; Analysis 11.2). Neither study reported on treatment withdrawals.

##### Duration of free NRT

Abdullah 2013 compared abstinence rates when participants were provided with two weeks versus one week of free NRT (participants were encouraged to use NRT for eight to 12 weeks in total). The point estimate favoured the longer duration of free treatment; however, the CIs also incorporate the possibility of no difference between groups (RR 1.63, 95% CI 0.98 to 2.70; 562 participants; Analysis 12.1). Burns 2016 provided participants with eight weeks versus four weeks of free NRT (participants were encouraged to use NRT for 10 weeks in total), and found no clear evidence of an effect on abstinence (RR 0.97, 95% CI 0.64 to 1.48; 1495 participants; Analysis 12.1). Neither study reported AEs.

#### Participant‐ versus clinician‐selected NRT

Walker 2011 found that providing participants with a one‐week free NRT selection box (including one patch, gum, inhaler, sublingual tablets and oral pouches), followed by eight weeks of free participant‐selected NRT did not result in higher quit rates than providing participants with eight weeks of clinician‐selected NRT patches or gum (RR 1.28, 95% CI 0.90 to 1.83; 1410 participants; Analysis 13.1). However, this RR and 95% CI are based on quit rates validated by saliva sample analysis (63/706 and 49/704 quit in the selection box and control groups, respectively), and a very low proportion of participants who claimed to have quit completed verification (34%). We therefore conducted a sensitivity analysis using data extrapolated from validated proportions to the wider trial population (161/706 and 136/704 quit in the selection box and control groups, respectively: RR 1.18, 95% CI 0.96 to 1.45; 1410 participants), and non‐verified, self‐reported quit rates (143/706 and 133/704 quit in the selection box and control groups, respectively: RR 1.07, 95% CI 0.87 to 1.33; 1410 participants). None of the three analyses showed clear evidence of between‐group differences, and there were no differences in clinical interpretation across sensitivity analyses (Analysis 13.1). Walker 2011 also found no evidence of a difference in SAEs between groups, with wide CIs (RR 1.04, 95% CI 0.72 to 1.50; 1 study, 1410 participants; Analysis 13.6).

#### Other variations in NRT use

Tulloch 2016 was not entered into any meta‐analyses. Although it compared combination patch plus fast‐acting NRT to patch alone, there were other variations in the NRT use that may have confounded the effect. The patches used in the combination arm were self‐titrated to a maximum of 35 mg and used over 22 weeks, whereas the patches in the control arm were a maximum of 21 mg (depending on cigarettes per day), used over 10 weeks with tapering of dose from week seven. The study found higher quit rates in the intervention group (29/233 quit) versus the control group (23/230 quit); however, CIs included the possibility of no difference and of the opposite effect (RR 1.25, 95% CI 0.75 to 2.10; 486 participants; Analysis 13.1). Furthermore, the study found no clear evidence of a difference between the intervention and control groups for cardiac AEs (RR 0.60, 95% CI 0.14 to 2.48; 1 study, 490 participants; Analysis 13.3), SAEs (RR 0.67, 95% CI 0.24 to 1.84; 1 study, 490 participants; Analysis 13.6) or withdrawals due to treatment (RR 1.25, 95% CI 0.34 to 4.60; 1 study, 490 participants; Analysis 13.7), with all point estimates accompanied by wide CIs.

Similarly, LeBlanc 2017 compared 10 weeks of combination patch plus fast‐acting NRT (nicotine inhaler) to 10 weeks of patch treatment alone. Patches used in the combination arm were also titrated based on smoking history, while patches used alone were of standard dose (definition of standard dose not specified), thus potentially confounding the effect. At 52‐week follow‐up, this study reported a difference in quit rates of 5.4% in biochemically‐confirmed abstinence rates between groups, with imprecise CIs (OR 1.51, 95% CI 0.76 to 3.02; 303 participants).

Finally, Berlin 2011 compared (1) providing a nicotine dose aimed at substituting 100% (± 5%) of participants' nicotine, prescribed based on the previous week's saliva cotinine concentrations and delivered in the form of a patch, with gum or lozenge (as needed) to (2) standard care, in which nicotine patches were delivered by fixed monthly dose decreases (mixed based on dependence) with gum or lozenge (as needed). At six months' follow‐up, the study found no clear evidence of a difference between study groups for outcomes of smoking cessation (RR 1.03, 95% CI 0.67 to 1.57; 1 study, 310 participants; Analysis 13.1), chest pain (RR 0.71, 95% CI 0.23 to 2.20; 1 study, 310 participants; Analysis 13.4), palpitations (RR 2.50, 95% CI 0.49 to 12.69; 1 study, 310 participants; Analysis 13.5) or SAEs (RR 0.75, 95% CI 0.33 to 1.73; 1 study, 310 participants; Analysis 13.6).

## Discussion

### Summary of main results

This review summarises and evaluates the evidence investigating the relative efficacy and safety of different types of nicotine replacement therapy (NRT) use for smoking cessation, including variations in duration, dose and modes of delivery. A review of NRT versus controls for smoking cessation has already been published (Hartmann‐Boyce 2018). It provides high‐certainty evidence that offering NRT to people who are dependent on smoking but prepared to attempt to quit increases their chance of success over that achieved with the same level of support but without NRT. This review adds to those findings by investigating different NRT use approaches, to understand which approaches maximise the likelihood of smoking cessation at six months or longer.

This review includes 68 completed studies investigating the effects of: NRT dose; duration of treatment; use in combination versus single form; different types of NRT; a fixed versus ad lib dosing schedule; preloading; and the provision of free NRT. All studies reported smoking abstinence at least six months following baseline; however, cardiac adverse events (AEs), serious adverse events (SAEs) and withdrawals due to treatment were all measured variably and infrequently.

This review update continues to provide high‐certainty evidence that the use of combination NRT results in higher quit rates than single‐form NRT. This finding held true regardless of whether that single form was a patch or a fast‐acting version, such as gum or lozenge, or a choice of patch or fast‐acting single‐form NRT. For patch dose comparisons, we judged the evidence to be of moderate certainty due to imprecision. Of the patch dose comparisons, 21 mg patches resulted in higher quit rates than 14 mg (24‐hour) patches; 25 mg patches resulted in higher quit rates than 15 mg (16‐hour) patches, although the confidence interval (CI) included one; and there was no clear evidence of superiority for 42/44 mg over 21/22 mg (24‐hour) patches. In addition, results suggest that using 4 mg nicotine gum results in higher quit rates than using 2 mg nicotine gum. A post hoc subgroup analysis accounted for the moderate heterogeneity in the associated analysis. It indicated that this may only be true in smokers who are highly dependent, and that 4 mg and 2 mg gum may result in similar quit rates when used by people less dependent on smoking. However, this finding should be treated with caution and tested in primary, adequately‐powered studies to strengthen the evidence in this area. Moderate‐certainty evidence indicates that nicotine preloading (i.e. the use of NRT before the quit date) results in higher quit rates than using NRT from quit day onwards. However, when we removed the five studies (of nine) at high risk of bias from the analysis, the clear evidence of a positive effect did not remain. It is not possible to say conclusively that this was due to bias, and could be because removing more than half of the studies meant that the sample size was reduced by more than half, making the result less precise.

We found no clear evidence of an effect for: duration of nicotine patch use (low‐certainty evidence); 16‐hour versus 24‐hour daily patch use; duration of combination NRT use (low‐ and very low‐certainty evidence); tapering of patch dose versus abrupt patch cessation; fast‐acting NRT type (very low‐certainty evidence); duration of nicotine gum use; ad lib versus fixed dosing of fast‐acting NRT; free versus purchased NRT; length of provision of free NRT; ceasing versus continuous patch use on lapse; and participant‐ versus clinician‐selected NRT. However, this lack of evidence of an effect should not be interpreted as proof that these different forms of NRT will result in equal quit rates. In many cases, these findings are based on very low‐ or low‐certainty evidence and the findings of single studies. The exception to this is the high‐certainty evidence which suggests that using a form of fast‐acting NRT alone, such as gum or lozenge, results in similar quit rates to using a nicotine patch.

Many studies did not report cardiac AEs separately or did not report AEs and SAEs at all. Where these were reported, there was no evidence of differential cardiac AEs or overall SAEs across comparisons. Both rates were low or very low overall, except for one study of nicotine preloading, which found an excess of palpitations in the preloading arm. However, due to variations in reporting, we rate the evidence on which these findings were based as low or very low certainty. The number of withdrawals from trials reported to be due to treatment was also variably reported across studies. We rated the contributing evidence to be of low and very low certainty. For most comparisons, the frequency of these withdrawals was similar between groups; however, more withdrawals due to treatment were reported in participants using nasal spray (3.0%) in comparison to patch (0.9%) in one trial, and in participants using 42/44 mg patches (6.1%) in comparison to 21/22 mg patches (1.1%) across two trials (low‐certainty evidence). In both cases, the withdrawal rates due to treatment were low, so their clinical relevance may be limited when considered alongside other clinical factors, such as initial patient preference and efficacy.

### Overall completeness and applicability of evidence

We conducted broad searches for this review to identify any studies where NRT was used as treatment. Although there was no intention to update the review of NRT versus control (Hartmann‐Boyce 2018), updated search results were needed for a forthcoming component network meta‐analysis on pharmacological interventions for smoking cessation that will also include NRT versus control studies (Lindson 2022). Through this process of screening studies, we can be confident in our approach for identifying all studies that compared one form or delivery mode of NRT with another, regardless of how clear this was at the first stage of eligibility screening. We also searched trial registers to identify any ongoing or completed, but unpublished, registered studies comparing NRT to another form of NRT.

Although the evidence base investigating the efficacy of NRT versus control (no NRT) is considerable and judged to be stable and of high certainty (Hartmann‐Boyce 2018), the evidence base exploring the optimal methods of NRT use is less developed. Although this review includes 67 studies, there are many comparisons of interest; in many cases, the studies and participants contributing to a comparison are sparse, and further research could strengthen or change findings. Although smoking abstinence was reported in all included studies (as this was an inclusion criterion), AEs, SAEs and withdrawals due to treatment were reported rarely and inconsistently across studies, making it difficult to carry out meta‐analyses and draw conclusions.

Studies included in this review update recruited people who smoked, who were motivated to quit and were typically 18 years or older. Across the studies in this review, the highest mean number of cigarettes per day was 38. Caution should therefore be exercised when attempting to generalise results outside these populations. We did not consider the evidence on nicotine‐containing electronic cigarettes in this review, although they may be considered a mode of nicotine delivery. Studies of electronic cigarettes for smoking cessation are included in a separate review (Hartmann‐Boyce 2022).

### Certainty of the evidence

Of the 68 studies included in this review, we judged nine to be at low risk of bias for all domains, and 28 to be at high risk in one or more domains. We deemed the overall risk of bias for the remaining 31 studies to be unclear. In many cases, we had to rate studies at an unclear risk due to a lack of reporting of key information. To investigate the potential impact of studies that we judged to be at high risk of bias on results, we carried out sensitivity analyses, removing these studies and observing the effects on results. In most cases, this had no effect on the clinical interpretation of the analyses. However, removing the five studies we judged to be at high risk of bias from the analysis of NRT preloading versus NRT use from quit day onward did affect the results. Originally, the results showed a positive effect of NRT preloading on smoking quit rates with CIs only encompassing beneficial effects; however, after the five high‐risk studies were removed, the CIs widened and included the potential for no effect and for a benefit of the no preloading comparator. The direction of the effect of the pooled point estimate still favoured the intervention; however, the number of participants in the analysis halved, which will have contributed to the imprecision of the results. 

We did not assess the potential bias imposed by studies funded by industry in this review. Given we investigated variations in the form, dose, delivery, duration and schedule of NRT, rather than the effectiveness of NRT compared to a placebo, we deemed industry funding of studies less applicable in this context.

We assessed the certainty of the evidence by creating summary of findings tables and carrying out GRADE ratings for seven of the comparisons: combination versus single‐form NRT (Table 1); duration of combination therapy (Table 2); patch dose (Table 3); duration of patch use (Table 4); fast‐acting NRT versus nicotine patch (Table 5); type of fast‐acting NRT (Table 6); NRT preloading versus standard post‐quit use (Table 7), across all outcomes, where possible. Two of the seven comparisons we assessed generated high‐certainty evidence for the efficacy of treatment for smoking cessation: combination versus single‐form NRT, and fast‐acting NRT versus nicotine patch. We judged the NRT preloading versus standard post‐quit use comparison to generate moderate‐certainty evidence. We rated the remaining efficacy comparisons to be of low or very low certainty. In all cases where data were available to contribute to any of the safety analyses for these comparisons, we rated the evidence to be of low or very low certainty. This was largely due to the fact that very few studies contributed data to these analyses, and where they did, the number of events was very low. We present effect estimates as risk ratios, as these are easier to interpret than odds ratios, but this means that where there are no events measured in both comparison groups, risk ratios cannot be calculated and therefore do not contribute to the meta‐analysis. We considered alternative statistical approaches to dealing with this data analysis. However, we concluded that other approaches would be more difficult to interpret and that overall conclusions would not change as a result.

The main reasons for downgrading the evidence were imprecision (low overall numbers of participants and events), risk of bias (judgements of high risk that may affect the result) and heterogeneity (high statistical heterogeneity detected in meta‐analyses).

### Potential biases in the review process

We consider the review process used to be robust and do not believe we have introduced any biases. We followed the standard methods for outcome assessment for Cochrane Tobacco Addiction Review Group cessation reviews. Our search strategy included the Cochrane Tobacco Addiction Group Specialised Register, and we captured an ongoing study. However, there may be unpublished data that our searches did not uncover. We did not screen the references lists of included studies uniformly across versions of this review. We also considered participants lost to follow‐up as continuing to smoke, which is current best practice in this field of work (West 2005). Due to the limited number of studies contributing to each comparison, we could only create one funnel plot, comparing combination NRT to single‐form NRT. This provided no evidence of publication bias, although only 16 studies contributed (a relatively small number), so this should be interpreted with caution. 

### Agreements and disagreements with other studies or reviews

There is high‐certainty evidence to suggest that NRT is a safe and effective treatment for quitting smoking (Hartmann‐Boyce 2018). Evidence for the effect of NRT relative to other pharmacotherapies for smoking cessation can be found in a Cochrane Reviews of nicotine agonists (Livingstone‐Banks 2023), as well as in a Cochrane Review of antidepressants for smoking cessation (Hajizadeh 2023). In addition, there is a Cochrane overview of pharmacotherapies for smoking cessation, which also provides indirect comparisons (Cahill 2013); a new, revised version of this network meta‐analysis is expected to be available in 2023 (Lindson 2022). Thomas 2022 also conducted a network meta‐analysis investigating the comparative effectiveness and safety of pharmacotherapies for smoking cessation. The combined evidence from these reviews suggests that, overall, NRT is as effective a quitting aid as the antidepressant bupropion but is less effective than the nicotine agonist varenicline. However, availability of varenicline is limited due to production issues at the time of writing, and there is evidence that combination NRT is as effective as varenicline. This aligns with the findings of this review, which has found high‐certainty evidence that combination NRT is more effective than single forms of NRT.

Clinical practice guidelines in the USA (Fiore 2008; Patnode 2021; US Preventive Services Task Force 2021), New Zealand (NZ MoH 2021), and England (NICE 2022) are consistent with the finding that combination NRT is more effective than single forms of NRT, although British prescribing guidelines do not mention the combination of different forms of NRT (BNF 2018). National Institute for Health and Care Excellence (NICE) guidance does not currently recommend nicotine preloading and explicitly recommends starting NRT on the day before the target quit date (NICE 2022). Preloading is not addressed in some American guidance (Fiore 2008), and is not explicitly recommended in British and other American guidance (BNF 2018; Patnode 2021; US Preventive Services Task Force 2021). British prescribing guidelines and some American guidelines support the use of higher‐dose preparations of NRT in people highly dependent on smoking (BNF 2018; Fiore 2008); this is not included in the US Preventive Services Task Force 2021 guidance. National guidelines have given less consideration to the other comparisons addressed by this review. New Zealand's Ministry of Health guidelines for helping people stop smoking recommend at least eight weeks of NRT use, and state that people can use NRT for longer than 12 weeks when needed (NZ MoH 2021). Currently, our findings do not find clear evidence of increased effectiveness of longer NRT use. However, our confidence in the evidence for this finding ranges from low to very low certainty, and more evidence would aid our interpretation. Appendix 3 highlights key elements of British prescribing guidance (British National Formulary (BNF)) as these relate to the comparisons in this review.

## Authors' conclusions

**Implications for practice**

- Combination NRT (fast‐acting form plus patch) results in approximately 17% to 37% higher long‐term quit rates than a single form of NRT.
- 4 mg nicotine gum results in approximately 12% to 83% higher quit rates than 2 mg nicotine gum, although there is some evidence to suggest this may vary based on nicotine dependence.
- Forms of fast‐acting NRT, such as gum and lozenge, are as effective a cessation aid as nicotine patches.
- There is some evidence that using 21 mg (24‐hour) nicotine patches results in higher quit rates than 14 mg (24‐hour) nicotine patches; however, further evidence could strengthen or weaken this effect.
- There is some evidence that using NRT before a quit day could result in higher quit rates than beginning NRT on a quit day; however, due to potential risks of bias in the existing studies, further research could strengthen or weaken this effect.
- There is insufficient evidence indicating that any other characteristics of NRT influence the efficacy of NRT for smoking cessation.
- There is insufficient evidence to conclude whether different types or methods of NRT delivery result in more frequent cardiac adverse events (AEs), serious adverse events (SAEs) or withdrawals due to treatment. However, these events are rare, and NRT is generally considered to be well‐tolerated.
- These conclusions all apply to adults who smoke, who are motivated to quit and who smoke approximately 20 or more cigarettes per day. There is little evidence about the role of NRT for individuals smoking fewer than 15 cigarettes a day.

**Implications for research**

- More high‐quality studies are needed to assess the efficacy of higher versus lower patch doses, different durations of NRT treatment course, different types of fast‐acting NRT, and NRT preloading versus standard NRT use. In particular, well‐conducted studies examining the use of fast‐acting NRT or combination NRT for preloading would add to the existing evidence base. Studies in people smoking fewer than 15 cigarettes a day or more than 40 cigarettes a day, and in younger age groups, would also add to the existing evidence base.
- New studies should ensure that they report adverse events and withdrawals due to treatment, and that these numbers are reported separately by study arm, as well as overall.

## What's new

**Table:** 

**Date**	**Event**	**Description**
19 June 2023	New citation required but conclusions have not changed	Four new studies added with no change to conclusions or the certainty of the evidence contributing to key comparisons and outcomes
19 June 2023	New search has been performed	Four new studies added. Incorporates evidence up to 29 April 2022.

## History

Review first published: Issue 4, 2019

## Notes

Professor Chris Silagy died in December 2001. In recognition of his major contribution, he remained as first author of the review until 2007. The authorship changed from 2008 Issue 1.

## Appendices

### Appendix 1. Tobacco Addiction Group Specialised Register search strategy

#1 NRT: TI,AB,KY,XKY,MH,EMT

#2 (nicotine NEAR2 patch*):TI,AB,KY,XKY,MH,EMT

#3 (nicotine NEAR2 gum):TI,AB,KY,XKY,MH,EMT

#4 (nicotine NEAR2 nasal spray*):TI,AB,KY,XKY,MH,EMT

#5 (nicotine NEAR2 lozenge*):TI,AB,KY,XKY,MH,EMT

#6 (nicotine NEAR2 tablet*):TI,AB,KY,XKY,MH,EMT

#7 (nicotine NEAR2 sublingual):TI,AB,KY,XKY,MH,EMT

#8 (nicotine NEAR2 inhal*):TI,AB,KY,XKY,MH,EMT

#9 (nicotine NEAR2 strip*):TI,AB,KY,XKY,MH,EMT

#10 (nicotine NEAR2 microtab*):TI,AB,KY,XKY,MH,EMT

#11 (nicotine NEAR2 replacement):TI,AB,KY,XKY,MH,EMT

#12 (nicotine NEAR3 therap*):TI,AB,KY,XKY,MH,EMT

#13 #1 OR #2 OR #3 OR #4 OR #5 OR #6 OR #7 OR #8 OR #9 OR #10 OR #11 OR #12

The specialised register was transferred from Reference Manager to the Cochrane Register of Studies in May 2012. This is the search used for the CRS: KY, XKY, MH & EMT are keyword fields.

### Appendix 2. Withdrawals, cardiovascular adverse events, and serious adverse events by study

**Table:**

**Study ID**	**Withdrawals due to treatment**	**Cardiovascular adverse events (AEs)**	**Serious adverse events (SAEs)**	**Notes**
Abdullah 2013	Not reported	Not reported	Not reported	No AE data reported
Baker 2016	Not reported	Not reported	0/421 combination group; 0/241 patch group.	AEs measured for duration of treatment (12 weeks). Only most common AEs reported (i.e. in > 5% of participants).
Berlin 2011	Not reported	Standard care: Chest pain: 7/155; Palpitations: 2/155; Dose adaptation: Chest pain: 5/155; Palpitations: 5/155	12/155 Standard care; 9/155 Dose adaptation	AEs measured for duration of study.
Blondal 1999	Not reported	Not reported	Not reported	AEs measured during treatment (at 3 months). Not reported in detail by relevant trial arms.
Bohadana 2000	Not reported	Not reported	1/200 intervention group; 1/200 control group. Both unrelated to treatment.	AEs measured at 1 year. Treatment was for 6 months. Only most common AEs reported.
Bolin 1999	Not reported	Not reported	Not reported	No AEs data reported
Bolliger 2007	Not reported	Not reported	Not reported	AEs measured at each visit to 1 year. Treatment was for 12 weeks. Only most common AEs reported (i.e. in > 5% of participants)
Bullen 2010	Not reported	Cardiac: 10/549 (1.8%) pre‐cessation group; 8/551 (1.5%) control group Unspecified chest pain: 9/549 pre‐cessation group; 1/551 control group	Number of participants: 11/549 intervention group; 7/551 control group. Total number of events: 99/549 intervention group; 109/551 control group.	AEs measured at all contacts (6 months). Cardiac AEs numerator is number of people experiencing AEs.
Burns 2016	Not reported	Not reported	Not reported	No AEs data reported
Caldwell 2014	Not reported	Not reported	Not reported	AEs measured at 1 year. Treatment was for 6 months.
Caldwell 2016	15/246 (6.1%) nicotine patch plus inhaler; 3/256 (1.2%) nicotine patch plus placebo inhaler	Chest discomfort: baseline, active 3/246 vs control 1/256. One day quit, active 1/224 vs control 0/234. 1 month quit, active 2/170 vs control 0/179. 3 months quit, active 4/147 vs control 0/143. 6 months quit, active 0/128 vs control 0/119. Palpitations: baseline, active 3/246 vs control 0/256. 1 day quit, active 6/224 vs control 4/234. 1 month quit, active 4/170 vs control 2/179. 3 months quit, active 1/147 vs control 2/143. 6 months quit, active 2/128 vs control 0/119	5/246 nicotine patch and inhaler group; 0/256 nicotine patch and placebo group.	AEs measured during treatment (6 months)
CEASE 1999	72 (2%) overall. Not reported by relevant trial arm.	Palpitations and tachycardia: 32/1430 (2.3%) 25 mg group; 37/1431 (2.6%) 15 mg group	Do not report all SAEs. Not reported by length of treatment. Myocardial infarction 1/1430 25 mg group; 2/1431 15 mg group.	AEs during treatment (8 weeks). SAEs measured during whole study period. Not reported in detail by relevant trial arms.
Cooney 2009	0% overall.	Cardiac (related to treatment): 0/45 (0%) nicotine patch and active gum group; 0/51 (0%) nicotine patch and placebo gum group.	Not reported	AEs measured during treatment (6 months).
Croghan 2003	4/459 (0.9%) patch group; 14/463 (3%) spray group; 2/462 (0.4%) combined group.	Not reported	Not reported	AEs measured to 6 months. Treatment was for 6 weeks. Only most common AEs reported. "No other AEs were reported with a great deal of frequency"
Cummings 2011	Not reported	Not reported	Not reported	No AE data reported
Dale 1995	1/18 (5.6%) 44 mg group; 0/17 (0%) 22 mg group.	Not reported	Not reported	AEs (nicotine toxicity only, not including cardiac) measured during first week of treatment (inpatient phase). Treatment continued for 6 weeks
Daughton 1991	2 (1.3%) participants overall. Not reported by trial arm.	Not reported	Not reported	AEs measured weekly during treatment (4 weeks). Only most common AEs reported (i.e. in > 5% of participants)
Dennis 2016	Not reported	Not reported	Not reported	No AE data reported
Dignan 2019	Not reported	Not reported	Not reported	No AE data reported
Garvey 2000	2/203 4 mg gum group; 1/202 2 mg gum group	Not reported	Not reported	AEs not reported in detail by relevant trial arms.
Garvey 2006	Not reported	Not reported	Not reported	No AE data reported
Glavas 2003	1/40 3‐week group (additional person withdrew as perceived treatment as ineffective); 2/40 6‐week group	Cardiac: 0/40 (0%) 3‐week group; 0/40 (0%) 6‐week group	0/40 intervention group; 0/40 control group.	AEs measured during treatment (3 weeks or 6 weeks depending on treatment group)
Goldstein 1989	Not reported	Not reported	Not reported	No AE data reported
Hall 2009	Not reported	Midsternal pressure: 1/203 (0.5%) extended (50 week) NRT group; 0/199 (0%) in brief (10 week) NRT group	9/203 extended (50 week) NRT group; 4/199 brief (10 week) NRT group. CARDIAC SAEs: 4/203 extended (50 week) NRT group; 0/199 brief (10 week) NRT group.	AEs measured to week 104. Treatment was to week 50.
Hays 1999	Not reported	Cardiovascular (angina pectoris, cardiovascular disorder, chest pain, and/or myocardial infarction): 5/321 (1.6%) free patches group; 9/315 (2.9%) pay for patches group	SAEs not fully reported. 5 cardiovascular SAEs in trial (2 myocardial infarction: 1 in known NRT arm, 1 in placebo arm) (not used in this review).	AEs measured during treatment (6 weeks)
Herrera 1995	Not reported	Not reported	Not reported	Adverse effects measured daily during treatment. Tachycardia was observed. Not reported in detail by relevant trial arms.
Hilleman 1994	7/69 (10%) fixed dose; 8/71 (11%) tapered dose	Not reported	Not reported	Some AE data reported. Time measured not reported.
Hughes 1990	Not reported	Not reported	Not reported	AEs (not including cardiac) measured during treatment (at 1 week).
Hughes 1991	Not reported	Not reported	Not reported	No AE data reported
Hughes 1999	3/260 (1%) 21 mg group; 8/260 (3%) 35 mg group; 16/259 (6%) 42 mg group	Cardiac (mostly tachycardia, vasodilation, and palpitation): 8% of 42 mg group, not reported for other groups	3/259 42 mg group; 1/260 35 mg group; 1/260 21 mg group	Withdrawals in first 4 months. AEs measured to 6 or 12 months depending on site. Treatment was for 16 weeks. AEs not reported in detail by relevant trial arms
Hughes 2018	9% overall. Not reported by trial arm	Not reported	4/356 continue patch group; 4/345 discontinue patch group. 1 SAE in each group was cardiac‐related.	AEs measured to 1 week post treatment (12 weeks). Only most common AEs reported
Jorenby 1995	Not reported	Not reported	4/252 44 mg intervention group (2 cardiovascular: stroke and myocardial infarction); 0/252 control group	AEs measured weekly during treatment (8 weeks). Only most common AEs reported
Kalman 2006	Not reported	Not reported	Not reported	AEs measured during treatment (up to 12 weeks post‐quit)
Killen 1990	21/152 (13.7%) ad lib group; 16/147 (12.5%) fixed group	Not reported	Not reported	AEs measured weekly during treatment (8 weeks). Only most common AEs reported (10 most common)
Killen 1999	Not reported	Irregular heartbeat: 21/206 (10%) 25 mg group; 20/202 (10%) 15 mg group Severe irregular heartbeat: 5/206 (2.4%) 25 mg group; 6/202 (3%) 15 mg group	Not reported	AEs self‐reported by participants. Measured during treatment (to 6 weeks)
Kornitzer 1987	Not reported	Not reported	Not reported	No AE data reported
Kornitzer 1995	1/149 (0.7%) nicotine patch and gum group; 2/150 (1.3%) nicotine patch and placebo gum group	Not reported	Not reported	AEs measured at each visit during treatment (6 months). Not reported in detail by relevant trial arms
Krupski 2016	Not reported	Not reported	Not reported	No AE data reported
Kupecz 1996	0/21 (0%) patch group; 4/17 (23%) gum group	Cardiac: 0/21 (0%) patch group; 0/17 (0%) gum group	0/21 patch group; 0/17 gum group	AEs measured at each session to 1 year. Treatment was for 24 weeks. AEs presented here measured at 6 weeks (during treatment)
LeBlanc 2017	Not reported	Not reported	Not reported	No AEs data reported
Lerman 2004	Not reported	Not reported	0/175 patch group; 0/175 spray group	AEs measured in counselling sessions during treatment (8 weeks)
Leung 2019	Not reported	Palpitations: 3/286 single therapy (nicotine patch); 0/274 combined NRT group	Not reported	Single NRT group: 14 withdrawn from study due to refusal, side effects, geographical reason, and death from cancer. Combined NRT group: 7 withdrawn from study due to refusal, geographical reason and death from cancer. In the single NRT group, 12 (4.2%) reported side effects from nicotine patch. In the combined NRT group, 7 (2.6%) reported side effects from NRT. There was no significant difference between the two groups (P = 0.315).
Moolchan 2005	Not reported	Not reported	Not reported	AEs measured during treatment (12 weeks). Only most common AEs reported (19 most common)
Paoletti 1996	Not reported	Not reported	Not reported	AEs measured at visits. Participants were asked about particular symptoms but none cardiac. Paper states, "Heart rate and blood pressure were not affected by the different treatments."
Piper 2009	0/260 (0%) lozenge group; 0/262 (0%) patch and lozenge group	Not reported	32 SAEs in 6 months. Not reported by trial arm	AEs measured at visits during treatment (8 weeks). No SAEs were possibly related to treatment and no withdrawals due to AEs in relevant trial arms.
Piper 2016	Not reported	Not reported	0 SAEs in any group. 0 cardiac SAEs in any group.	AEs measured to 26 weeks. Not reported in detail by relevant trial arms
Puska 1995	Not reported	Not reported	Not reported	AEs measured at all visits during treatment (52 weeks). Only moderate or severe AEs reported
Rey 2009	2 (4%) participants overall. Not reported by trial arm	Not reported	Not reported	No AE data reported
Rose 1994	Not reported	Not reported	Not reported	AEs measured until 1 week after treatment. Only AEs relating to mecamylamine treatment discussed
Rose 1998	0/40 (0%) preloading group; 1/40 (2.5%) no preloading group	Not reported	Not reported	AEs measured during preloading period. 5 people withdrew for reasons unrelated to treatment.
Rose 2006	Not reported	Not reported	Not reported	No AE data reported
Rose 2009	Not reported	Not reported	1/191 preloading nicotine patch group; 3/188 preloading placebo patch group	Timing of AEs measurements not reported. AEs only reported if self‐reported severity was moderate or greater
Rose 2010	3% overall. Not reported by trial arm.	Not reported	Not reported	AEs measured during treatment (12 weeks). Not reported in detail by relevant trial arms
Schlam 2016	Not reported	Not reported	10/275 26‐week patch group; 6/269 8‐week patch group. CARDIAC SAEs: 4/275 26‐week patch group; 5/269 8‐week patch group	AEs measured to 1 year. Treatment was for 8 or 26 weeks. Only most common AEs reported. SAE data from clinicaltrials.gov. Paper states no SAE in trial
Schnoll 2010a	1/282 (0.4%) extended treatment group; 0/282 (0%) standard treatment group	Pounding heart: Week 1: 2/247 (0.8%) extended group; 3/252 (1.2%) standard group. Week 12: 0/182 (0%) extended group; 2/134 (1.5%) standard group.	3/282 extended NRT group (including 1 myocardial infarction); 1/286 standard NRT group	AEs measured to 1 year. Treatment was for 8 or 24 weeks. AE denominators are participants followed. The myocardial infarction occurred before treatment started
Schnoll 2010b	Not reported	Not reported	4/321 patch group (including 2 strokes); 7/321 lozenge group (including 1 heart disease and 1 myocardial infarction)	AEs measured to 6 months. Treatment was for 12 weeks. AEs not reported in detail by relevant trial arms. All SAEs considered unrelated to study treatment (as did not occur whilst on treatment) except stroke in patch group.
Schnoll 2015	Not reported	Pounding heart: at 12 weeks: 0/128 (0%) 8‐week group; 1/137 (0.7%) 24‐week group; 2/121 (1.7%) 52‐week group. At 30 weeks: 2/103 (1.9%) 8‐week group; 1/116 (0.9%) 24‐week group; 1/103 (1.0%) 52 week group Rapid heartbeat: 1/103 (1%) 8‐week group; 1/116 (0.9%) 24‐week group; 0/103 (0%) 52‐week group	4/180 8‐week patch group; 2/173 24‐week patch group; 8/172 52‐week patch group	Cardiac AEs are not cumulative across time points.
Schuurmans 2004	Not reported	Not reported	Not fully reported. One death in each group	AEs measured at all follow‐up visits (to 6 months). Treatment was for 12 weeks. AEs not reported in detail by relevant trial arms
Smith 2009	Not reported	Not reported	Not reported	No AE data reported
Smith 2013	Not reported	Not reported	0/490 2‐week NRT group; 0/497 6‐week NRT group; 0/494 patch group; 0/493 patch and gum group	No AE data reported
Stapleton 1995	8 (2%) overall. Not reported by trial arm	Not reported	Not reported	AEs measured at each visit. Not reported in detail by relevant trial arms
Preloading Investigators 2018	Not reported	Palpitations: 35/899 (3.9%) preloading group; 17/893 (1.9%) control group	8/899 preloading group (3 cardiac); 8/893 control group (0 cardiac)	AEs measured to 1 week post‐quit (1 week after preloading ceased)
TNSG 1991	11/262 (4.2%) 21 mg group; 15/275 (5.5%) 14 mg group; 1/127 (0.8%) 7 mg group	Not reported	0 SAEs in any group	AEs not reported in detail by relevant trial arms
Tønnesen 1988	0/27 (0%) 4 mg group; 1/33 (3%) 2 mg group	Palpitations: 1/27 (3.7%) 4 mg group; 0/33 (0%) 2 mg group	Not reported	AEs measured in counselling sessions during treatment (either 16 or 20 weeks)
Tønnesen 1996	0/45 (0%) ad libitum group; 0/44 (0%) fixed group	Palpitations: at 1 week: 1 moderate and 1 severe overall (not spilt by treatment group). At 6 weeks: 0% in both groups	0 SAEs in any group	AEs measured on treatment (up to 6 weeks)
Tønnesen 2000	Not reported	Not reported	0/109 5 mg patch group; 0/104 15 mg patch group; 0/118 inhaler group; 0/115 inhaler and 15 mg patch group	AEs measured at every follow‐up (to 12 months). Treatment could continue to 12 months
Tulloch 2016	5/245 (2%) patch and gum group; 4/245 (1.6%) patch group	Cardiovascular (e.g. palpitations, tachycardia, chest pain): 3/245 (1.2%) patch and fast‐acting NRT group; 5/245 (2%) patch only group	6/245 patch and gum group; 9/245 patch group	AEs measured at each appointment
Walker 2011	Not reported	Not reported	53/706 selection box group; 51/704 usual care group	SAEs measured to 6 months. Treatment was for 8 weeks.

### Appendix 3. British National Formulary prescribing guidance for NRT as relates to comparisons in this review

**Table:**

**Comparison of interest**	**BNF recommendation**	**Review findings**
Patch duration	“Individuals who smoke more than 10 cigarettes daily should apply a high‐strength patch daily for 6 ‐ 8 weeks, followed by the medium‐strength patch for 2 weeks and then the low‐strength patch for the final 2 weeks; individuals who smoke fewer than 10 cigarettes daily can usually start with the medium‐strength patch for 6 ‐ 8 weeks, followed by the low‐strength patch for 2 ‐ 4 weeks” > 10 cigarettes per day: 10 to 12 weeks < 10 cigarettes per day: 8 to 12 weeks	Low‐certainty evidence of no effect of duration of nicotine patch use on smoking cessation. Studies in the review typically recruited smokers who were smoking at least 15 cigarettes per day so comparisons with BNF guidance for individuals smoking < 10 cigarettes per day cannot be made.
Patch dose	“Individuals who smoke more than 10 cigarettes daily should apply a high‐strength patch… individuals who smoke fewer than 10 cigarettes daily can usually start with the medium‐strength patch…” > 10 cigarettes per day: high strength (21/22/25 mg) then tapered < 10 cigarettes per day: medium strength (15 mg) then tapered	Moderate‐certainty evidence that 21 mg patches result in higher quit rates than 14 mg 24‐hour patches Moderate‐certainty evidence that 25 mg patches result in higher quit rates than 15 mg (16‐hour) patches, though the CI includes one. Moderate‐certainty evidence that 42/44 mg patches (not available in UK) are as effective as 21/22 mg patches Low‐certainty evidence of no difference of dose on serious adverse events or treatment withdrawals Studies in the review typically recruited smokers who were smoking at least 15 cigarettes per day so comparisons with BNF guidance for individuals smoking < 10 cigarettes per day cannot be made.
**Patch tapering**	“Individuals who smoke more than 10 cigarettes daily should apply a high‐strength patch daily for 6‐8 weeks, followed by the medium‐strength patch for 2 weeks and then the low‐strength patch for the final 2 weeks; individuals who smoke fewer than 10 cigarettes daily can usually start with the medium‐strength patch for 6‐8 weeks, followed by the low‐strength patch for 2‐4 weeks” > 10 cigarettes per day: 6 to 8 weeks high strength, 2 weeks medium strength, 2 weeks low strength < 10 cigarettes per day: 6 to 8 weeks medium strength, 2 to 4 weeks low strength	No evidence of difference between tapering and abrupt patch cessation on abstinence Studies in the review typically recruited smokers who were smoking at least 15 cigarettes per day so comparisons with BNF guidance for individuals smoking < 10 cigarettes per day cannot be made.
**Patch 16‐hour versus 24‐hour** ** **	No reference to hours of use per day	No evidence of effect of hours of use per day on abstinence.
**Ceasing versus continuing on lapse** ** **	“[If] abstinence is not achieved, or if withdrawal symptoms are experienced, the strength of the patch used should be maintained or increased until the patient is stabilised” Continue on lapse	No evidence of effect on abstinence of instructing participants to continue using a patch versus stopping patch use, in the event of a smoking lapse.
**Patch preloading** ** **	No specific reference but does refer to using patch prior to quit day to reduce cigarette consumption: “a slower titration schedule can be used [for patches] in individuals who are not ready to quit but want to reduce cigarette consumption before a quit attempt”	Moderate‐certainty evidence of a positive effect of NRT preloading on abstinence
**Combination NRT** ** **	No reference to combination NRT	High‐certainty evidence that combination NRT results in higher long‐term quit rates, whether combination therapy was compared to patch or to a fast‐acting form of NRT. Low‐ to very low‐certainty evidence of no effect on cardiac adverse events, serious adverse events or study withdrawals
**Type of NRT** ** **	No recommendations on which type of NRT to use.	High‐certainty evidence of no difference between fast‐acting NRT and patch on smoking cessation Very low‐certainty evidence of no difference in effect of type of fast‐acting NRT (oral spray, gum or inhaler) on smoking cessation
**Gum dose**	“In individuals who smoke fewer than 20 cigarettes each day… 2 mg as required, chew 1 piece of gum when the urge to smoke occurs or to prevent cravings” “In individuals who smoke more than 20 cigarettes each day or who require more than 15 pieces of 2 mg strength gum each day… 4 mg as required, chew 1 piece of gum when the urge to smoke occurs or to prevent cravings, individuals should not exceed 15 pieces of 4 mg strength gum daily” > 20 cigarette a day: 4 mg < 20 cigarette a day: 2 mg	Evidence that using 4 mg gum results in higher quit rates than 2 mg gum. A post hoc subgroup analysis found a statistically significant benefit of 4 mg dose over 2 mg dose for higher‐dependency smokers, but not for lower‐dependency smokers.
**Duration of gum** ** **	“Treatment should continue for 3 months before reducing the dose”	No significant effect of 50 weeks gum over 10 weeks gum use on smoking cessation
**Fixed dose versus ad lib dosing for fast‐acting NRT** ** **	Gum: “Chew 1 piece of gum when the urge to smoke occurs or to prevent cravings” Sublingual tablet: “1 [or 2] tablet[s] every 1 hour” Inhalator: “As required, the cartridges can be used when the urge to smoke occurs or to prevent cravings” Lozenges: “1 lozenge every 1‐2 hours as required, one lozenge should be used when the urge to smoke occurs” Oromucosal spray: “1‐2 sprays as required, individuals can spray in the mouth when the urge to smoke occurs or to prevent cravings” Nasal spray: “1 spray as required, individuals can spray into each nostril when the urge to smoke occurs, up to twice every hour for 16 hours daily...maximum 64 sprays per day.” Advice differs by type of fast‐acting NRT. Ad lib for gum and nasal spray	No evidence of an effect of fixed versus ad lib dosing of fast‐acting NRT (gum and nasal spray) on abstinence
As specified in the Methods section, we only carried out GRADE assessments and created summary of findings tables for some of the comparisons (and their associated outcomes) in this review. Therefore, only some of the review findings above are accompanied by a GRADE rating of the certainty of the evidence. **CI:** confidence interval; **NRT:** nicotine replacement therapy		

## Data and analyses

**Comparison 1:** Patch dose

Outcome or subgroup title	No. of studies	No. of participants	Statistical method	Effect size
1.1 Smoking cessation	9		Risk Ratio (M‐H, Fixed, 95% CI)	Subtotals only
1.1.1 21 mg versus 14 mg (24‐hour)	1	537	Risk Ratio (M‐H, Fixed, 95% CI)	1.48 [1.06, 2.08]
1.1.2 25 mg versus 15 mg (16‐hour)	3	3446	Risk Ratio (M‐H, Fixed, 95% CI)	1.19 [1.00, 1.41]
1.1.3 42/44 mg versus 21/22 mg (24‐hour)	5	1655	Risk Ratio (M‐H, Fixed, 95% CI)	1.09 [0.93, 1.29]
1.2 Fast or irregular heartbeat	2	3269	Risk Ratio (M‐H, Fixed, 95% CI)	0.92 [0.64, 1.33]
1.3 Myocardial infarction	1		Risk Ratio (M‐H, Fixed, 95% CI)	Totals not selected
1.4 Overall serious adverse events	3		Risk Ratio (M‐H, Fixed, 95% CI)	Subtotals only
1.4.1 42/44 mg versus 21/22 mg (24‐hour)	2	1023	Risk Ratio (M‐H, Fixed, 95% CI)	5.01 [0.87, 28.82]
1.4.2 21 mg versus 14 mg (24‐hour)	1	0	Risk Ratio (M‐H, Fixed, 95% CI)	Not estimable
1.5 Treatment withdrawals	3		Risk Ratio (M‐H, Fixed, 95% CI)	Subtotals only
1.5.1 42/44 mg versus 21/22 mg (24‐hour)	2	554	Risk Ratio (M‐H, Fixed, 95% CI)	4.99 [1.60, 15.50]
1.5.2 21 mg versus 14 mg (24‐hour)	1	537	Risk Ratio (M‐H, Fixed, 95% CI)	0.77 [0.36, 1.64]

**Comparison 2:** Duration of patch therapy

Outcome or subgroup title	No. of studies	No. of participants	Statistical method	Effect size
2.1 Smoking cessation	7		Risk Ratio (M‐H, Fixed, 95% CI)	Subtotals only
2.1.1 52 weeks versus 24 weeks	1	345	Risk Ratio (M‐H, Fixed, 95% CI)	0.78 [0.53, 1.15]
2.1.2 52 weeks versus 8 weeks	1	352	Risk Ratio (M‐H, Fixed, 95% CI)	0.94 [0.63, 1.41]
2.1.3 28 weeks versus 12 weeks	1	2861	Risk Ratio (M‐H, Fixed, 95% CI)	1.05 [0.88, 1.26]
2.1.4 24 weeks versus 8 weeks	2	921	Risk Ratio (M‐H, Fixed, 95% CI)	1.10 [0.84, 1.45]
2.1.5 12 weeks versus 6 weeks	1	140	Risk Ratio (M‐H, Fixed, 95% CI)	1.03 [0.62, 1.71]
2.1.6 12 weeks versus 3 weeks	1	98	Risk Ratio (M‐H, Fixed, 95% CI)	0.61 [0.26, 1.41]
2.1.7 6 weeks versus 4 weeks	1	1873	Risk Ratio (M‐H, Fixed, 95% CI)	1.06 [0.85, 1.33]
2.1.8 6 weeks versus 2 ‐ 3 weeks	2	1957	Risk Ratio (M‐H, Fixed, 95% CI)	1.13 [0.91, 1.40]
2.1.9 4 weeks versus 2 weeks	1	1862	Risk Ratio (M‐H, Fixed, 95% CI)	1.08 [0.85, 1.37]
2.2 Overall serious adverse events	3		Risk Ratio (M‐H, Fixed, 95% CI)	Subtotals only
2.2.1 52 weeks versus 24 weeks	1	345	Risk Ratio (M‐H, Fixed, 95% CI)	4.02 [0.87, 18.67]
2.2.2 52 weeks versus 8 weeks	1	352	Risk Ratio (M‐H, Fixed, 95% CI)	2.09 [0.64, 6.82]
2.2.3 24 weeks versus 8 weeks	2	921	Risk Ratio (M‐H, Fixed, 95% CI)	1.03 [0.30, 3.54]
2.2.4 6 weeks versus 2 ‐ 3 weeks	1	0	Risk Ratio (M‐H, Fixed, 95% CI)	Not estimable
2.3 Treatment withdrawals	2		Risk Ratio (M‐H, Fixed, 95% CI)	Totals not selected
2.3.1 24 weeks versus 8 weeks	1		Risk Ratio (M‐H, Fixed, 95% CI)	Totals not selected
2.3.2 6 weeks versus 2 ‐ 3 weeks	1		Risk Ratio (M‐H, Fixed, 95% CI)	Totals not selected

**Comparison 3:** Effect of tapering patch dose

Outcome or subgroup title	No. of studies	No. of participants	Statistical method	Effect size
3.1 Smoking cessation	2	264	Risk Ratio (M‐H, Fixed, 95% CI)	0.99 [0.74, 1.32]
3.2 Treatment withdrawals	1		Risk Ratio (M‐H, Fixed, 95% CI)	Totals not selected

**Comparison 4:** Combination versus single‐form NRT

Outcome or subgroup title	No. of studies	No. of participants	Statistical method	Effect size
4.1 Smoking cessation	16	12169	Risk Ratio (M‐H, Fixed, 95% CI)	1.27 [1.17, 1.37]
4.1.1 Combination NRT versus patch alone	13	9552	Risk Ratio (M‐H, Fixed, 95% CI)	1.24 [1.13, 1.37]
4.1.2 Combination NRT versus fast‐acting NRT alone	6	2364	Risk Ratio (M‐H, Fixed, 95% CI)	1.30 [1.09, 1.54]
4.1.3 Combination NRT versus choice of single form NRT (patch or fast‐acting)	1	253	Risk Ratio (M‐H, Fixed, 95% CI)	5.16 [1.18, 22.59]
4.2 Any cardiac adverse event	2	656	Risk Ratio (M‐H, Fixed, 95% CI)	0.66 [0.22, 2.05]
4.3 Overall serious adverse events	5	2888	Risk Ratio (M‐H, Fixed, 95% CI)	4.44 [0.76, 25.85]
4.3.1 Combination NRT versus patch alone	4	2313	Risk Ratio (M‐H, Fixed, 95% CI)	11.45 [0.64, 205.90]
4.3.2 Combination NRT versus fast‐acting NRT alone	2	575	Risk Ratio (M‐H, Fixed, 95% CI)	1.00 [0.06, 15.88]
4.4 Treatment withdrawals	5	3070	Risk Ratio (M‐H, Fixed, 95% CI)	1.12 [0.57, 2.20]
4.4.1 Combination NRT versus patch alone	5	1982	Risk Ratio (M‐H, Fixed, 95% CI)	2.32 [0.99, 5.40]
4.4.2 Combination NRT versus fast‐acting NRT alone	2	1088	Risk Ratio (M‐H, Fixed, 95% CI)	0.14 [0.02, 1.08]

**Comparison 5:** Duration of combination therapy

Outcome or subgroup title	No. of studies	No. of participants	Statistical method	Effect size
5.1 Smoking cessation	2		Risk Ratio (M‐H, Fixed, 95% CI)	Subtotals only
5.1.1 16 weeks versus 8 weeks	1	637	Risk Ratio (M‐H, Fixed, 95% CI)	0.96 [0.75, 1.23]
5.1.2 6 weeks versus 2 weeks	1	987	Risk Ratio (M‐H, Fixed, 95% CI)	1.11 [0.94, 1.31]
5.2 Overall serious adverse events	3		Risk Ratio (M‐H, Fixed, 95% CI)	Totals not selected
5.2.1 26 weeks versus 8 weeks	1		Risk Ratio (M‐H, Fixed, 95% CI)	Totals not selected
5.2.2 16 weeks versus 8 weeks	1		Risk Ratio (M‐H, Fixed, 95% CI)	Totals not selected
5.2.3 6 weeks versus 2 weeks	1		Risk Ratio (M‐H, Fixed, 95% CI)	Totals not selected

**Comparison 6:** Fast‐acting NRT versus patch

Outcome or subgroup title	No. of studies	No. of participants	Statistical method	Effect size
6.1 Smoking cessation	8	3319	Risk Ratio (M‐H, Fixed, 95% CI)	0.90 [0.77, 1.05]
6.1.1 Inhaler versus patch	1	222	Risk Ratio (M‐H, Fixed, 95% CI)	0.59 [0.22, 1.60]
6.1.2 Nasal spray versus patch	2	1272	Risk Ratio (M‐H, Fixed, 95% CI)	0.90 [0.64, 1.27]
6.1.3 Lozenge versus patch	3	1707	Risk Ratio (M‐H, Fixed, 95% CI)	0.94 [0.79, 1.12]
6.1.4 Gum versus patch	2	118	Risk Ratio (M‐H, Fixed, 95% CI)	0.58 [0.26, 1.31]
6.2 Cardiac adverse events	1		Risk Ratio (M‐H, Fixed, 95% CI)	Totals not selected
6.3 Overall serious adverse events	4		Risk Ratio (M‐H, Fixed, 95% CI)	Totals not selected
6.3.1 Inhaler versus patch	1		Risk Ratio (M‐H, Fixed, 95% CI)	Totals not selected
6.3.2 Nasal spray versus patch	1		Risk Ratio (M‐H, Fixed, 95% CI)	Totals not selected
6.3.3 Lozenge versus patch	1		Risk Ratio (M‐H, Fixed, 95% CI)	Totals not selected
6.3.4 Gum versus patch	1		Risk Ratio (M‐H, Fixed, 95% CI)	Totals not selected
6.4 Treatment withdrawals	3	1482	Risk Ratio (M‐H, Fixed, 95% CI)	4.23 [1.54, 11.63]
6.4.1 Nasal spray versus patch	1	922	Risk Ratio (M‐H, Fixed, 95% CI)	3.47 [1.15, 10.46]
6.4.2 Gum versus patch	1	38	Risk Ratio (M‐H, Fixed, 95% CI)	11.00 [0.63, 191.04]
6.4.3 Lozenge versus patch	1	0	Risk Ratio (M‐H, Fixed, 95% CI)	Not estimable

**Comparison 7:** Type of fast‐acting NRT

Outcome or subgroup title	No. of studies	No. of participants	Statistical method	Effect size
7.1 Smoking cessation	1		Risk Ratio (M‐H, Fixed, 95% CI)	Totals not selected
7.1.1 Oral spray versus gum	1		Risk Ratio (M‐H, Fixed, 95% CI)	Totals not selected
7.1.2 Oral spray versus inhaler	1		Risk Ratio (M‐H, Fixed, 95% CI)	Totals not selected
7.1.3 Gum versus inhaler	1		Risk Ratio (M‐H, Fixed, 95% CI)	Totals not selected

**Comparison 8:** 4 mg versus 2 mg gum

Outcome or subgroup title	No. of studies	No. of participants	Statistical method	Effect size
8.1 Smoking cessation	5	856	Risk Ratio (M‐H, Fixed, 95% CI)	1.43 [1.12, 1.83]
8.1.1 High‐dependency smokers	4	618	Risk Ratio (M‐H, Fixed, 95% CI)	1.85 [1.36, 2.50]
8.1.2 Low‐dependency smokers	3	238	Risk Ratio (M‐H, Fixed, 95% CI)	0.77 [0.49, 1.21]
8.2 Palpitations	1		Risk Ratio (M‐H, Fixed, 95% CI)	Totals not selected
8.3 Treatment withdrawals	2	465	Risk Ratio (M‐H, Fixed, 95% CI)	1.08 [0.18, 6.36]

**Comparison 9:** Fixed versus ad lib dose schedule

Outcome or subgroup title	No. of studies	No. of participants	Statistical method	Effect size
9.1 Smoking cessation	4	828	Risk Ratio (M‐H, Fixed, 95% CI)	1.12 [0.87, 1.45]
9.1.1 Gum	2	689	Risk Ratio (M‐H, Fixed, 95% CI)	1.22 [0.92, 1.61]
9.1.2 Nasal spray	2	139	Risk Ratio (M‐H, Fixed, 95% CI)	0.67 [0.35, 1.30]
9.2 Overall serious adverse events	1		Risk Ratio (M‐H, Fixed, 95% CI)	Totals not selected
9.3 Treatment withdrawals	2		Risk Ratio (M‐H, Fixed, 95% CI)	Totals not selected
9.3.1 Gum	1		Risk Ratio (M‐H, Fixed, 95% CI)	Totals not selected
9.3.2 Nasal spray	1		Risk Ratio (M‐H, Fixed, 95% CI)	Totals not selected

**Comparison 10:** Preloading versus standard use

Outcome or subgroup title	No. of studies	No. of participants	Statistical method	Effect size
10.1 Smoking cessation	9	4395	Risk Ratio (M‐H, Fixed, 95% CI)	1.25 [1.08, 1.44]
10.1.1 Patch	9	3830	Risk Ratio (M‐H, Fixed, 95% CI)	1.28 [1.09, 1.49]
10.1.2 Gum	2	306	Risk Ratio (M‐H, Fixed, 95% CI)	0.93 [0.58, 1.49]
10.1.3 Patch + gum	2	259	Risk Ratio (M‐H, Fixed, 95% CI)	1.35 [0.80, 2.28]
10.2 Palpitations	1		Risk Ratio (M‐H, Fixed, 95% CI)	Totals not selected
10.3 Cardiac adverse events	1		Risk Ratio (M‐H, Fixed, 95% CI)	Totals not selected
10.4 Cardiac serious adverse events	3	3529	Risk Ratio (M‐H, Fixed, 95% CI)	1.94 [0.81, 4.65]
10.5 Overall serious adverse events	4	3908	Risk Ratio (M‐H, Fixed, 95% CI)	1.11 [0.59, 2.09]
10.6 Treatment withdrawals	1		Risk Ratio (M‐H, Fixed, 95% CI)	Totals not selected

**Comparison 11:** Free NRT versus purchased NRT

Outcome or subgroup title	No. of studies	No. of participants	Statistical method	Effect size
11.1 Smoking cessation	2	740	Risk Ratio (M‐H, Fixed, 95% CI)	1.38 [0.90, 2.13]
11.1.1 Patch	1	636	Risk Ratio (M‐H, Fixed, 95% CI)	1.24 [0.77, 1.99]
11.1.2 Gum	1	104	Risk Ratio (M‐H, Fixed, 95% CI)	2.70 [0.89, 8.20]
11.2 Cardiac adverse events	1		Risk Ratio (M‐H, Fixed, 95% CI)	Totals not selected

**Comparison 12:** Duration of free NRT

Outcome or subgroup title	No. of studies	No. of participants	Statistical method	Effect size
12.1 Smoking cessation	2		Risk Ratio (M‐H, Fixed, 95% CI)	Subtotals only
12.1.1 2 weeks versus 1 week patch or gum	1	562	Risk Ratio (M‐H, Fixed, 95% CI)	1.63 [0.98, 2.70]
12.1.2 8 weeks versus 4 weeks patch	1	1495	Risk Ratio (M‐H, Fixed, 95% CI)	0.97 [0.64, 1.48]

**Comparison 13:** Other comparisons

Outcome or subgroup title	No. of studies	No. of participants	Statistical method	Effect size
13.1 Smoking cessation	6		Risk Ratio (M‐H, Fixed, 95% CI)	Totals not selected
13.1.1 24‐hour versus 16‐hour patch	1		Risk Ratio (M‐H, Fixed, 95% CI)	Totals not selected
13.1.2 50 weeks versus 10 weeks gum	1		Risk Ratio (M‐H, Fixed, 95% CI)	Totals not selected
13.1.3 Continue versus stop patch use on lapse	1		Risk Ratio (M‐H, Fixed, 95% CI)	Totals not selected
13.1.4 35 mg patch + fast‐acting for 22 weeks versus 21 mg patch for 10 weeks	1		Risk Ratio (M‐H, Fixed, 95% CI)	Totals not selected
13.1.5 NRT tester period + choice versus clinician‐advised	1		Risk Ratio (M‐H, Fixed, 95% CI)	Totals not selected
13.1.6 100% nicotine dose substitution based on weekly saliva cotinine concentration versus nicotine patch with monthly dose decreases plus gum or lozenge at investigator discretion	1		Risk Ratio (M‐H, Fixed, 95% CI)	Totals not selected
13.2 Midsternal pressure	1		Risk Ratio (M‐H, Fixed, 95% CI)	Totals not selected
13.2.1 50 weeks versus 10 weeks gum	1		Risk Ratio (M‐H, Fixed, 95% CI)	Totals not selected
13.3 Cardiac adverse events	1		Risk Ratio (M‐H, Fixed, 95% CI)	Totals not selected
13.4 Chest pain	1		Risk Ratio (M‐H, Fixed, 95% CI)	Totals not selected
13.5 Palpitations	1		Risk Ratio (M‐H, Fixed, 95% CI)	Totals not selected
13.6 Overall serious adverse events	5		Risk Ratio (M‐H, Fixed, 95% CI)	Totals not selected
13.6.1 50 weeks versus 10 weeks gum	1		Risk Ratio (M‐H, Fixed, 95% CI)	Totals not selected
13.6.2 Continue versus stop patch use on lapse	1		Risk Ratio (M‐H, Fixed, 95% CI)	Totals not selected
13.6.3 35 mg patch + fast‐acting for 22 weeks versus 21 mg patch for 10 weeks	1		Risk Ratio (M‐H, Fixed, 95% CI)	Totals not selected
13.6.4 NRT tester period + choice versus clinician advised	1		Risk Ratio (M‐H, Fixed, 95% CI)	Totals not selected
13.6.5 100% nicotine dose substitution adapted based on weekly saliva cotinine concentration versus monthly decreases in nicotine dose	1		Risk Ratio (M‐H, Fixed, 95% CI)	Totals not selected
13.7 Treatment withdrawals	1		Risk Ratio (M‐H, Fixed, 95% CI)	Totals not selected
13.7.1 35 mg patch + fast‐acting for 22 weeks versus 21 mg patch for 10 weeks	1		Risk Ratio (M‐H, Fixed, 95% CI)	Totals not selected

## Characteristics of studies

### Characteristics of included studies [ordered by study ID]

**Table:** Abdullah 2013

***Study characteristics***		
Methods	Study design: parallel RCT Country: China Recruitment: from a smoking cessation health centre ‐ smokers who called the booking line and attended the health centre during the study period were recruited by smoking cessation counsellor	
Participants	562 smokers: aged ≥ 16 years, ≥ 5 cigarettes per day, clearly motivated to quit 78.3% men; average cigarettes per day: 18.8; average years smoking: 18.5	
Interventions	1) 2 weeks of free NRT (patch or gum according to participant preference). However, participants were encouraged to use NRT for 8 to 12 weeks, sourcing the remainder themselves. 2) 1 week of free NRT (patch or gum according to participant preference). However, participants were encouraged to use NRT for 8 to 12 weeks, sourcing the remainder themselves.	
Outcomes	PPA at 6‐month follow‐up; CO validated (< 9 ppm) Other abstinence measures: self‐reported 7‐day PPA at 6 months; self‐reported 24‐hour PPA at 6 and 12 months; self‐reported continuous at 6 and 12 months; quit for at least 24 hours at some point before 6‐ and 12‐month follow‐up Adverse events: not measured	
Notes	70% of participants chose patch, 30% chose gum, with similar between‐group percentages The study was funded by the Hong Kong Council on Smoking and Health (COSH). Pfizer Consumers and Novartis partially sponsored the printing cost of the clinic pamphlets and provided some free NRT samples. Conflicts of interest: the authors declared no conflict of interests	
***Risk of bias***		
**Bias**	**Authors' judgement**	**Support for judgement**
Random sequence generation (selection bias)	Low risk	Quote: “The random numbers for group assignment were generated by the research assistant (not the counselors) of the project using a personal computer before subject recruitment.”
Allocation concealment (selection bias)	Low risk	Quote: “Eligible selected subjects signed the consent form and completed the baseline measures…before the counselor opened a serially numbered, opaque, and sealed envelope (SNOSE) to reveal the random assignment of each smoker to A1 or A2 group.”
Blinding (performance bias and detection bias) All outcomes	Low risk	Quote: “An independent interviewer, who was unaware of the subject’s group allocation, carried out the 6 and 12 months follow‐up interview.” Participants were aware whether they were provided 1 or 2 weeks of free NRT; however, it would be impossible to blind for this
Incomplete outcome data (attrition bias) All outcomes	Low risk	Dropout rates at 6 months were 75/278 in group 1 (2 weeks of free NRT) and 83/284 in group 2 (1 week of free NRT). There was therefore less than 50% dropout overall and rates were similar between groups.

**Table:** Baker 2016

***Study characteristics***		
Methods	Study design: parallel RCT Country: USA Recruitment: participants were recruited from 2 sources: (1) by contacting participants in the authors' ongoing longitudinal study of smokers, the Wisconsin Smokers Health Study; and (2) by media and community outreach	
Participants	1086 smokers (662 in relevant trial arms): aged > 17 years, ≥ 5 cigarettes per day, desire to quit smoking but not engaged in smoking treatment, willingness to use the tested cessation treatments and not using e‐cigarettes 47.9% men; average age: 48.1 years; average cigarettes per day: 17; average FTND: 4.8; average exhaled CO: 15.1 ppm	
Interventions	1) Combination NRT: nicotine patch (12 weeks: 21 mg for 8 weeks, 14 mg for 2 weeks, 7 mg for 2 weeks) and lozenge (12 weeks: 2 mg or 4 mg based on addiction level, asked to use at least 5 lozenges a day) 2) Nicotine patch only (12 weeks: 21 mg for 8 weeks, 14 mg for 2 weeks, 7 mg for 2 weeks) In both groups, treatment began on quit day.	
Outcomes	7‐day PPA at 52 weeks' follow‐up; CO validated (≤ 5 ppm) Other abstinence measures: 7‐day PPA at 26 weeks with CO validation; self‐reported prolonged abstinence at 26 weeks (no smoking from day 7 to day 181 post‐quit day) Adverse events: measured for duration of treatment (12 weeks)	
Notes	This was a 3‐arm trial comparing varenicline, nicotine patch and nicotine patch plus lozenge. For the purposes of this review, we are only interested in the nicotine patch and nicotine patch plus lozenge groups. The study was funded by grant 5R01HL109031 from the National Heart, Lung, and Blood Institute and grant K05CA139871 from the National Cancer Institute. Conflicts of interest: Dr Stein reports receipt of data and safety monitoring board honoraria from Lilly and Abbott. No other disclosures were reported.	
***Risk of bias***		
**Bias**	**Authors' judgement**	**Support for judgement**
Random sequence generation (selection bias)	Low risk	Quote: “Computer‐based randomization”
Allocation concealment (selection bias)	Low risk	Quote: “Computer‐based randomization”
Blinding (performance bias and detection bias) All outcomes	High risk	At risk of both performance and detection bias Quote: “Treatment assignment was unblinded” Quote: “The follow‐up telephone assessments were intended to be blinded, but a database search by interviewers could have revealed treatment assignment.”
Incomplete outcome data (attrition bias) All outcomes	Low risk	Overall loss to follow‐up across all 3 groups at 12 months = 22.5%. Loss to follow‐up of 22.4% in nicotine patch group and 21.6% in the combination NRT group; therefore similar between trial arms of interest. We obtained information on losses to follow‐up directly from the study authors.

**Table:** Berlin 2011

***Study characteristics***		
Methods	Study design: parallel RCT Country: France Recruitment: people attending smoking cessation clinics were invited to participate	
Participants	310 smokers with medical comorbidities ('known smoking‐related disorder or an underlying disease with increased risk for smoking‐related illnesses') 62.6% men; mean age: 50 years; ≥ 10 cigarettes per day; average cigarettes per day: 25.4; motivated to quit	
Interventions	1) Standard care: nicotine patches with monthly dose decreases; buccal absorption NRT products of gum (2 mg) or lozenges (1.5 mg) could be co‐administered at the discretion of the investigator. Patch dose regime was mixed, based on dependence (FTND score and number of cigarettes per day). Co‐administration of a second nicotine patch was not permitted. FTND ≥ 5 or cigarettes per day ≥ 20: 21 mg 24‐hour patch followed by a monthly decrease to 21 mg/24 hours to 7 mg/24 hours. FTND < 5 or cigarettes per day < 20: 14 mg 24‐hour patch for 2 months; 7 mg/24 hours during month 3. 2) Dose adaptation: 'received a nicotine dose (either by patch or buccal absorption NRT or both) according to the saliva cotinine concentration based on a saliva cotinine/daily NRT dose conversion factor of 0.1'; aimed for 100% (± 5%) substitution. All participants received counselling (type and content at discretion of investigator) for at least 10 minutes at each visit, starting at the pre‐quit inclusion visit.	
Outcomes	Continuous abstinence at 6 months' follow‐up Validation: CO validated (≤ 8 ppm) Serious adverse events and adverse events	
Notes	Funding: "The trial was funded by the Programme Hospitalier de Recherche Clinique (PHRC) Loco‐regional 2004, registration number: 050558 and Agence française de sécurité sanitaire des produits de santé (AFSSAPS), Convention Pharmacologie Clinique et Thérapeutique 2003. Nicotine patches, nicotine gums and nicotine lozenges were generously provided by Pierre Fabre Santé. The study’s sponsor was Assistance publique‐Hôpitaux de Paris, registration number: AOR04001//P040406. The sponsor or the funding sources had no role in the design, conduct of the study; in the collection, analysis and interpretation of the data; or in the preparation, review or approval of the manuscript. The views and opinions expressed in this manuscript are those of the authors and should not be construed to represent the views of any of the sponsors." Conflicts of interest: "I. Berlin reports having received occasional honoraria for participation on the advisory boards of Sanofi‐Aventis, Pfizer Ltd; he is an employee of Assistance publique‐ Hôpitaux de Paris—Université P and M.Curie‐Faculté de médecine. N. Jacob reports no conflict of interest; she is an employee of Assistance publique‐Hôpitaux de Paris. M. Coudert reports no conflict of interest; he is an employee of the Clinical Research Unit, Assistance publique‐Hôpitaux de Paris. J. Perriot reports having received honoraria." This study was a previously excluded study in Lindson 2019. We reassessed it and deemed it eligible, and thus it is new to this current update version.	
***Risk of bias***		
**Bias**	**Authors' judgement**	**Support for judgement**
Random sequence generation (selection bias)	Low risk	"A computer‐generated list containing 400 numbers was created independently of the coordination centre and investigators."
Allocation concealment (selection bias)	Low risk	Randomisation list was incorporated into people’s electronic medical record and so assigned without investigator intervention
Blinding (performance bias and detection bias) All outcomes	High risk	Single‐blinded study. Investigators were aware of group allocation.
Incomplete outcome data (attrition bias) All outcomes	Low risk	At 6‐month follow‐up, 59% in the standard arm and 61% in the dose adaptation arm were followed up.

**Table:** Blondal 1999

***Study characteristics***		
Methods	Study design: parallel RCT Country: Iceland Recruitment: community volunteers	
Participants	237 smokers (≥ 1 cigarettes per day) 33% men, average age 41 to 43, average tobacco use 25 g/day	
Interventions	1) Nicotine nasal spray (NNS) (0.5 mg/dose) plus 15 mg nicotine patches for 3 months, weaning over further 2 months. NNS could be continued for 1 year 2. Placebo nasal spray plus 15 mg nicotine patches on same schedule	
Outcomes	Sustained abstinence at 12 months (6‐year data also reported) Validation: CO < 10 ppm Adverse events: measured within 3 months of follow‐up (still using NRT)	
Notes	6‐year abstinence 19/118 versus 10/119, OR 2.1 Pharmacia and Upjohn provided the drugs and placebo for this study and measured the cotinine concentrations. Conflicts of interest: TB was a consultant for Pharmacia and Upjohn, and GG and AW are employed by Pharmacia and Upjohn	
***Risk of bias***		
**Bias**	**Authors' judgement**	**Support for judgement**
Random sequence generation (selection bias)	Low risk	Quote: "computer generated randomisation code at a local pharmacy"
Allocation concealment (selection bias)	Low risk	Quote: "Pharmacy staff were blinded to the content of the bottles"
Blinding (performance bias and detection bias) All outcomes	Low risk	Clinic staff, pharmacy staff and participants were all blinded to assignment. Codes not broken until after data entry and analyses completed
Incomplete outcome data (attrition bias) All outcomes	Low risk	All participants followed up for at least 12 months

**Table:** Bohadana 2000

***Study characteristics***		
Methods	Study design: parallel RCT Country: France Recruitment: community volunteers	
Participants	400 smokers, 18 to 70 years, > 10 cigarettes per day, > 1 previous quit attempt, motivated 49% men, average cigarettes per day: Group 1: 26.1, Group 2: 23.5; FTND > 6 Participants required to be motivated to quit	
Interventions	1) Nicotine inhaler, 26 weeks, combined with nicotine patch (15 mg/16‐hour) for first 6 weeks, placebo patch for next 6 weeks 2) Nicotine inhaler, 26 weeks, placebo patch for first 12 weeks	
Outcomes	Sustained abstinence at 12 months (prolonged from week 2, no slips allowed) Validation: CO < 10 ppm at each visit (2 weeks, 6 weeks, 6 months, 12 months) (Study also reports respiratory symptoms and pulmonary function tests for completely abstinent participants) Adverse events: measured to 1‐year follow‐up (treatment ceased at 6 months)	
Notes	Gender subgroup results reported 2003 This study was supported by a grant from Pharmacia and Upjohn Consumer Healthcare. Conflicts of interest: not reported	
***Risk of bias***		
**Bias**	**Authors' judgement**	**Support for judgement**
Random sequence generation (selection bias)	Low risk	Quote: "computer‐generated randomization code"
Allocation concealment (selection bias)	Low risk	Quote: "sealed randomization envelopes were provided for each subject and were held by the hospital pharmacy, which was responsible for dispensing medication"
Blinding (performance bias and detection bias) All outcomes	Unclear risk	Described as double‐blind
Incomplete outcome data (attrition bias) All outcomes	Unclear risk	Losses over 12 months were steep but similar in both groups, i.e. 148 from NRT group and 155 from placebo group. Losses counted as continuing smokers

**Table:** Bolin 1999

***Study characteristics***		
Methods	Study design: parallel RCT Country: USA Recruitment: smoking cessation clinic	
Participants	98 smokers 84% men, average age 54, average cigarettes per day 20	
Interventions	1) Nicotine patch for 12 weeks (21 mg/3 weeks, 14 mg/3 weeks, 7 mg/3 weeks) 2) Nicotine patch for 3 weeks (21 mg/1 week, 14 mg/1 week, 7 mg/1 week)	
Outcomes	Continuous abstinence at 5 months (PPA also recorded) Validation: CO Adverse events: not measured	
Notes	Borderline follow‐up length ‐ 20 weeks from beginning of programme, 16 weeks from start of NRT Funding and declarations of interest not reported	
***Risk of bias***		
**Bias**	**Authors' judgement**	**Support for judgement**
Random sequence generation (selection bias)	Unclear risk	Quote: "Participants were randomly assigned ... random assignment took place on the first day of patch administration"
Allocation concealment (selection bias)	Unclear risk	Insufficient information
Blinding (performance bias and detection bias) All outcomes	Low risk	Quote: "Both participants and experimenters were unaware of assignment during the baseline phase of the study"
Incomplete outcome data (attrition bias) All outcomes	Unclear risk	Dropout rates not reported; any dropouts counted as treatment failures in analysis

**Table:** Bolliger 2007

***Study characteristics***		
Methods	Study design: parallel RCT Country: South Africa Recruitment: by a newspaper advertisement	
Participants	100 smokers: aged ≥ 18 years, > 15 cigarettes per day, smoked for > 3 years, exhaled CO > 10 ppm, serious quit attempts in the past 12 months, willing to stop smoking immediately 60% men; average age: 43.1 years; average cigarettes per day: 23.4; average FTND: 5.6; average exhaled CO: 25.5 ppm	
Interventions	1) Nicotine mouth spray 2) Nicotine gum 3) Nicotine inhaler Participants in all groups were advised to use their allocated product for 12 weeks from quit day, ad libitum (recommended 6 to 12 actuations/cartridges a day)	
Outcomes	Continuous smoking abstinence at 6‐month follow‐up (not a puff since quit day); CO‐validated (< 10 ppm) Other abstinence measures: self‐reported continuous at 12‐month follow‐up; self‐reported PPA at 12‐month follow‐up; CO‐validated PPA at 6 months Adverse events: measured at each visit to final follow‐up at 1 year (treatment only lasted 12 weeks)	
Notes	The trial was fully funded by NicoNovum AB (the pharmaceutical company who manufactured the mouth spray tested) Conflicts of interest: not reported	
***Risk of bias***		
**Bias**	**Authors' judgement**	**Support for judgement**
Random sequence generation (selection bias)	Unclear risk	Insufficient details given to make a judgement Quote: “Subjects were then randomly allocated (block randomization of 4, i.e. after each block of 4 subjects, 2 were allocated to the spray, 1 to the gum and 1 to the inhaler) to the mouth spray (n = 50), the gum (n = 25) and the inhaler (n = 25) group, irrespective of their preference.”
Allocation concealment (selection bias)	Unclear risk	As above
Blinding (performance bias and detection bias) All outcomes	High risk	Open‐label trial. No description is given of any attempts to blind participants or assessors. 7 participants changed their product during treatment: 2 from spray to gum and inhaler (1 each), 2 from gum to spray and inhaler (1 each), 3 from inhaler to spray (n = 2) and gum (n = 1); all 7 were considered treatment failures according to the principle of intention‐to‐treat
Incomplete outcome data (attrition bias) All outcomes	Unclear risk	Only 46% of participants attended final follow‐up (12 months), i.e. less than 50% of those randomised. There was differential dropout between groups (60% spray; 40% gum; 56% inhaler), with a 20% difference between the spray and gum groups.

**Table:** Bullen 2010

***Study characteristics***		
Methods	Study design: parallel RCT Country: New Zealand Recruitment: callers to New Zealand Quitline	
Participants	1100 smokers, motivated to quit 40% men; mean age: 40; average cigarettes per day: 19	
Interventions	Trial of pre‐cessation NRT Intervention: NRT initiated 14 days before quit date, continued for 8 weeks after quit date. 91% used patch only, 6% gum only, 3% both Control: NRT for 8 weeks from quit date. 85% patch, 11% gum, 4% both	
Outcomes	Continuous abstinence at 6 months (data supplied by 1st author) (Self‐reported 7‐day PPA at 6 months reported in paper) Validation: salivary cotinine in subgroup only. Self‐reported outcomes used in analysis Adverse events: measured at all contacts (assumed to be up to 6 months)	
Notes	Participants able to select their treatment (patch, gum, or patch plus gum) after discussion with adviser. Patch and gum outcomes supplied by 1st author; contribute to separate subgroups; 39 participants using combination not included in analysis. The study was funded by the Health Research Council and the Heart Foundation of New Zealand. HealthPAC approved the use of pre‐cessation NRT vouchers and the Pharmacy Guild of New Zealand supported the trial by alerting its member pharmacists to the PQNIQ trial and the special vouchers. Conflicts of interest: HM has received honoraria for speaking at research symposia and received benefits in kind and travel support from, and has provided consultancy to the manufacturers of smoking cessation medications, including those that manufacture nicotine patches and gum. MG has provided consultancy to the manufacturers of smoking cessation medications, including those that manufacture nicotine patches and gum.	
***Risk of bias***		
**Bias**	**Authors' judgement**	**Support for judgement**
Random sequence generation (selection bias)	Low risk	Quote: "People giving verbal consent by telephone were allocated randomly using central computerized randomization."
Allocation concealment (selection bias)	Low risk	Quote: "randomization sequence concealed until interventions were assigned"
Blinding (performance bias and detection bias) All outcomes	Unclear risk	No placebo. Single blinding: "Participants were aware of the group to which they were allocated but 3‐ and 6‐month follow‐up methods were identical for all participants, and all follow‐up telephone calls and outcome verification procedures were made by research assistants blind to treatment allocation."
Incomplete outcome data (attrition bias) All outcomes	Low risk	Similar rates of dropouts in treatment and control groups (148 treatment, 139 control). Participants lost to follow‐up included as smokers in outcome data

**Table:** Burns 2016

***Study characteristics***		
Methods	Study design: parallel RCT Country: USA Recruitment: by the Colorado quit‐line ‐ participants were recruited during regular initial quit‐line calls	
Participants	1495 smokers: smoking 16 to 20 cigarettes per day, agreed to receive free NRT, absence of a condition requiring physician approval for NRT 40.0% men; average cigarettes per day 19.8; most smoked within 5 minutes of waking and had been smoking for > 10 years	
Interventions	1) 4 weeks of free NRT (patches). However, participants were encouraged to complete 10 weeks of NRT, sourcing the remainder themselves. 2) 8 weeks of free NRT (patches), shipped in 2 x 4‐week batches. Participants were required to request the second batch. Participants were encouraged to complete 10 weeks of NRT, sourcing the remainder themselves.	
Outcomes	Self‐reported prolonged abstinence at 6‐month post‐quit day; no biochemical validation Other abstinence measures: self‐reported 7‐day and 30‐day PPA at 6 months Adverse events: not measured	
Notes	Only two‐thirds of group 2 (8 weeks of free NRT) accepted second 4‐week batch of NRT. Median time NRT used same in both groups (35 days) The study was funded by a Pfizer Scholar Grant in public health and the Colorado Department of Public Health and Environment contract number FLA‐11‐16830 Conflicts of interest: none	
***Risk of bias***		
**Bias**	**Authors' judgement**	**Support for judgement**
Random sequence generation (selection bias)	Unclear risk	Quote: "Enrolled study participants were randomised"; but no detail given on how randomisation took place
Allocation concealment (selection bias)	Unclear risk	As above. No detail on allocation concealment in text
Blinding (performance bias and detection bias) All outcomes	High risk	Quote: "Coaches ask all quitline enrolees during second and subsequent coaching calls about their NRT utilisation, and those who are eligible for a second shipment are asked whether they need it." No blinding. Although it would have been impossible to blind participants, it would have been possible to blind outcome assessors, and we therefore deem this study to be at high risk of detection bias
Incomplete outcome data (attrition bias) All outcomes	Low risk	Dropout rates at 6 month were 311/738 in group 1 (4 weeks of free NRT) and 321/757 (8 weeks of free NRT). There was less than 50% dropout overall and rates were similar between groups.

**Table:** Caldwell 2014

***Study characteristics***		
Methods	Study design: parallel RCT Country: New Zealand Recruitment: from media advertisements, clinician referrals, and a database of people interested in trying to stop smoking	
Participants	1423 smokers: aged 18 to 70 years, ≥ 9 cigarettes per day, FTND ≥ 3. Ineligible if currently taking psychoactive medication/illicit drugs, drank > 28 units of alcohol a week, had hyperthyroidism/diabetes/severe renal or hepatic disease, were female and using inadequate contraception or were breastfeeding 46% men; mean age 45; average cigarettes per day: 20; mean FTND: 6.1	
Interventions	1) 6 months of nicotine oral spray parallel to 5 months of free 24‐hour nicotine patch. Each spray actuation contained 1 mg nicotine. 2. 6 months of placebo oral spray parallel to 5 months of free 24‐hour nicotine patch. The placebo spray was dispensed in opaque bottles identical to the nicotine spray. Both groups were instructed to use the spray ad libitum whenever they felt the urge to smoke, up to a maximum of 30 sprays/day. Both groups received 21 mg/24‐hour nicotine patches for 18 weeks, then 14 mg/24‐hour nicotine patches for 2 weeks, and then 7 mg/24‐hour nicotine patches for 2 weeks.	
Outcomes	Prolonged abstinence at 12 months' post‐quit day; CO‐validated (< 10 ppm). Prolonged abstinence defined as no smoking since end of grace period (4 weeks after quit day) to 12 months' post‐quit day Other abstinence measures: 7‐day PPA at 12‐month follow‐up (CO‐validated) Adverse events: measured for 12 months (treatment was for 6 months)	
Notes	Authors provided information on dosing schedule Funding for the study was provided by the Health Research Council of New Zealand (HRC 09/200). Active Zonnic mouth‐spray was provided by Niconovum. Placebo Zonnic was manufactured by Argenta according to instructions from Niconovum. Nicotine patches were provided without charge by the New Zealand Ministry of Health. Conflicts of interest: none	
***Risk of bias***		
**Bias**	**Authors' judgement**	**Support for judgement**
Random sequence generation (selection bias)	Low risk	The randomisation sequence was computer‐generated. Quote: "Subjects were randomised centrally for all three trial sites using a random allocation algorithm built into the access database that was used for all of the data collection"
Allocation concealment (selection bias)	Low risk	Study participants were allocated into groups by a computer.
Blinding (performance bias and detection bias) All outcomes	Low risk	Double‐blinding maintained throughout trial Quote: "Active and placebo bottles were identical", "all staff remained blind to the allocation during the course of the trial"
Incomplete outcome data (attrition bias) All outcomes	High risk	Dropout rates at 12 months were 612/716 for group 1 (nicotine spray plus nicotine patch), and 621/707 for group 2 (placebo spray plus nicotine patch). There was more than 50% dropout overall, but rates were similar between groups.

**Table:** Caldwell 2016

***Study characteristics***		
Methods	Study design: parallel RCT Country: New Zealand Recruitment: from media advertisements, a study website, primary care practices and smoking cessation services	
Participants	502 smokers: aged 18 to 70 years, ≥ 9 cigarettes per day, FTND ≥ 3 49% men; mean age: 45; average cigarettes per day: 19; mean FTND: 6.2	
Interventions	1) 6 months of nicotine inhaler used parallel to 5 months of 24‐hour nicotine patch. The nicotine inhaler contained 2 doses of nicotine lactate: 100 micrograms/puff and 200 micrograms/puff. Participants were instructed to start with the lower dose and move onto the higher dose once they had developed tolerance to the upper airway effects of the lower dose. 2) 6 months of placebo inhaler used parallel to 5 months of 24‐hour nicotine patch. The placebo inhaler contained menthol in 2 doses to mimic the 2 doses of active inhaler and participants were also instructed to move onto the higher dose once they had developed tolerance to the upper airway effects of the lower dose. Both groups were instructed to use the inhaler when they had an urge to smoke, and to have as many puffs as required to satisfy their urge (maximum 10 puffs). Both groups were instructed to use 21 mg/24‐hour nicotine patch for 18 weeks, 14 mg/24‐hour for 2 weeks, and 7 mg/24‐hour for 2 weeks.	
Outcomes	Prolonged abstinence (defined as not even a puff) at 6‐month post‐quit date; CO‐validated at 1‐month visit (≤ 10 ppm) Other abstinence measures: self‐reported 7‐day PPA at 6 months, self‐reported prolonged abstinence at 6 months Adverse events: measured for 6 months (duration of treatment)	
Notes	Study funded by the Health Research Council of New Zealand (grant number 09/199) Conflicts of interest: none	
***Risk of bias***		
**Bias**	**Authors' judgement**	**Support for judgement**
Random sequence generation (selection bias)	Low risk	Quote: "Eligible subjects were randomised to active or placebo inhaler in a 1:1 ratio by the trial database according to a sequential randomisation list that was not visible to research staff or subjects"
Allocation concealment (selection bias)	Low risk	Allocation concealment upheld (see quote above)
Blinding (performance bias and detection bias) All outcomes	Low risk	Quote: "The database provided staff with a product code, which identified which inhaler to give to each subject. The product codes and inhalers for both groups had the same appearance... both subjects and staff were masked to treatment assignment"
Incomplete outcome data (attrition bias) All outcomes	Low risk	Dropout rates at 6 months were 88/246 in group 1 (nicotine inhaler plus nicotine patch), and 102/256 in group 2 (placebo inhaler plus nicotine patch). There was therefore less than 50% dropout overall and rates were similar between groups.

**Table:** CEASE 1999

***Study characteristics***		
Methods	Study design: factorial RCT Country: multicentre ‐ 36 clinic centres in 17 European countries Recruitment: community volunteers	
Participants	3575 smokers (> 14 cigarettes per day) 52% men, average age 41, average cigarettes per day: 27 (34% had previously used NRT)	
Interventions	Factorial design compared 2 patch doses and 2 treatment durations. Dose 15 mg or 25 mg (16‐hour), duration of active treatment 28 weeks (including 4‐week fading) or 12 weeks (including 4‐week fading) 1) 25 mg patch for 28 weeks 2) 25 mg patch for 12 weeks 3) 15 mg patch for 28 weeks 4) 15 mg patch for 12 weeks 5) Placebo	
Outcomes	Prolonged abstinence at 12 months, sustained from week 2 Validation: expired CO < 10 ppm at each clinic visit Adverse events: SAEs measured during whole study period, but cardiac AEs reported within 8‐week treatment period	
Notes	Level of support reclassified to high for 2007, because of repeated visits. Limited support at these visits This study was sponsored by Pharmacia and Upjohn Conflicts of interest: not reported	
***Risk of bias***		
**Bias**	**Authors' judgement**	**Support for judgement**
Random sequence generation (selection bias)	Low risk	Quote: "A computer‐generated allocation list was prepared centrally and allocated subjects to treatment numbers". Randomisation stratified by centre
Allocation concealment (selection bias)	Low risk	See process above
Blinding (performance bias and detection bias) All outcomes	Low risk	Quote: "Active and placebo patches were identical in appearance and packaging. In order to maintain blinding, all subjects continued to use two patches for a total of 26 weeks"; i.e. non‐tapered groups were switched to placebo patches
Incomplete outcome data (attrition bias) All outcomes	Low risk	22% lost to 12‐month follow‐up, and 54% withdrew

**Table:** Cooney 2009

***Study characteristics***		
Methods	Study design: parallel RCT Country: USA Recruitment: community volunteers and referrals from substance abuse clinic	
Participants	96 alcohol‐dependent tobacco smokers (≥ 15 cigarettes per day) 75% men, average age 45, average cigarettes per day 25, motivated to quit, average FTND 6, 31% veterans	
Interventions	1) Nicotine patch (titrated, 21 mg/day for 8 weeks, 14 mg/day for 2 weeks, 7 mg/day for 2 weeks) plus nicotine gum (2 mg for 24 weeks, ad lib but advised 6 to 20/day) 2) Nicotine patch plus placebo gum (doses as above)	
Outcomes	Continuous abstinence at 12 months (with 30‐day grace period immediately following quit date) Validation: CO < 10 ppm Adverse events: measured at 2 weeks, 3 months and 6 months (gum or placebo gum use continued until 6 months)	
Notes	This study was supported by award number R01 AA011197 and P50 AA1563 from the National Institute on Alcohol Abuse and Alcoholism and by a MIRECC award from the Department of Veterans Affairs. Conflicts of interest: JC and KS have worked as promotional speakers for Pfizer	
***Risk of bias***		
**Bias**	**Authors' judgement**	**Support for judgement**
Random sequence generation (selection bias)	Low risk	Quote: "urn randomization computer program that balanced the two groups for history of previous substance use treatment, age, sex, baseline drinks/drinking day and baseline [cigarettes per day]."
Allocation concealment (selection bias)	Low risk	Randomisation procedure required participant characteristics to be provided before allocation assigned
Blinding (performance bias and detection bias) All outcomes	Unclear risk	Quote: "Double blind." "Research assistants who collected these data were blind to medication assignment and did not conduct psychosocial treatments."
Incomplete outcome data (attrition bias) All outcomes	Low risk	26 dropouts at 12 months included as smokers; all previously verified as having relapsed

**Table:** Croghan 2003

***Study characteristics***		
Methods	Study design: parallel RCT Country: USA Recruitment: multicentre community volunteers	
Participants	1384 smokers (≥ 15 cigarettes per day) 42% men, average age 42, average cigarettes per day: 26	
Interventions	1) 15 mg/16‐hour nicotine patch plus 0.5 mg/dose nasal spray, maximum 5/hour, 40/day, for 6 weeks 2) Nicotine nasal spray only 3) Nicotine patch only	
Outcomes	PPA at 6 months Validation: CO Adverse events: measured to 6 months (treatment duration was 6 weeks)	
Notes	This study was supported in part by Public Health Service Grants CA‐25224, CA‐37404, CA63849, CA‐35269, CA‐52352, CA‐37417, CA‐63848, CA‐35195, and CA‐35103 from the National Cancer Institute, Department of Health and Human Services. Medication was provided by McNeil Consumer Products Conflicts of interest: not reported	
***Risk of bias***		
**Bias**	**Authors' judgement**	**Support for judgement**
Random sequence generation (selection bias)	Low risk	Randomisation by Mayo Clinic Co‐ordinating Centre
Allocation concealment (selection bias)	Low risk	Quote: "Treatment assignment was carried out using a dynamic allocation procedure" which took account of stratification by gender, cigarettes per day, years smoking, study site
Blinding (performance bias and detection bias) All outcomes	High risk	Open‐label study
Incomplete outcome data (attrition bias) All outcomes	Low risk	Dropouts reported in detail. 34% of participants completed study. Losses to follow‐up similar across groups, treated as non‐abstinent

**Table:** Cummings 2011

***Study characteristics***		
Methods	Study design: parallel RCT Country: USA Recruitment: from callers to the New York State Smokers’ Quit Line (NYSSQL) between July and October of 2008	
Participants	2806 smokers: aged ≥ 18 years, ≥ 10 cigarettes per day, interested in using nicotine patch to help them stop smoking, no known contraindications to the patch, willing to make quit attempt within 2 weeks 44.3% men; average age: 45 to 54 years (mode); average cigarettes per day: 20 to 29 (mode); time to first cigarette: within 5 minutes (mode category)	
Interventions	1) 2 weeks of free nicotine patch treatment provided 2) 4 weeks of free nicotine patch treatment provided 3) 6 weeks of free nicotine patch treatment provided All participants received the quit‐line’s standard cessation guide, providing tips on quitting smoking, along with information on the benefits of smoking cessation. In addition, all participants received 1 x 10‐ to 15‐minute proactive follow‐up call conducted 2 weeks after initially contacting the quit‐line. The counselling call was intended to help participants address barriers to quitting and prompt them to use the medications sent to them.	
Outcomes	Self‐reported 30‐day PPA at 7‐month follow‐up Other abstinence measures: self‐reported 7‐day PPA at 7 months No biochemical validation Adverse events: not measured	
Notes	Funded by the New York State Department of Health Conflicts of interest: not reported The mean number of patches used was significantly greater in the groups that received more medication (2‐week group: 13.0; 4‐week group: 16.3; 6‐week group: 20.1)	
***Risk of bias***		
**Bias**	**Authors' judgement**	**Support for judgement**
Random sequence generation (selection bias)	Unclear risk	Insufficient information given Quote: “Eligible participants were assigned according to a prerandomized assignment sheet”
Allocation concealment (selection bias)	Unclear risk	Insufficient information given Quote: “Eligible participants were assigned according to a prerandomized assignment sheet”
Blinding (performance bias and detection bias) All outcomes	High risk	Quote: “Quit line phone coaches were not aware of the callers’ group assignment.” However, participants were not blinded and it is unclear whether abstinence assessors were blind to allocation.
Incomplete outcome data (attrition bias) All outcomes	Low risk	59.9% of participants responded to the follow‐up survey overall, with a similar response rate between groups – 58% in 2‐week group; 62% in the 4‐week group; 60% in the 6‐week group

**Table:** Dale 1995

***Study characteristics***		
Methods	Study design: parallel RCT Country: USA Recruitment: community volunteers and smoking clinic attenders	
Participants	71 smokers stratified according to light, moderate and heavy smoking rates, and motivated to quit 44% men, average age 48, average cigarettes per day: 26	
Interventions	1) 11 mg/24‐hour nicotine patch 2) 22 mg/24‐hour nicotine patch 3) 44 mg/24‐hour nicotine patch 4) Placebo patch for 1 week followed by 11 or 22 mg patch for 7 weeks Duration of patch use: 8 weeks	
Outcomes	PPA at 12 months Validation: blood cotinine Adverse events: measured daily for 6 days post‐baseline (treatment continued for 6 weeks)	
Notes	This study was supported by Lederle Laboratories, Pearl River, NY. RH, IC and KO have worked on clinical research studies funded in part by Lederle Laboratories, Elan Pharmaceutical Research Corporation, Burroughs‐Wellcome and Kabi. Conflicts of interest: RH has received honoraria for educational activities from CibaGeigy Corporation, Marion Merrell Dow, Inc, and McNeil Pharmaceuticals. KO has received honoraria for educational activities from Elan Pharmaceutical Research Corporation.	
***Risk of bias***		
**Bias**	**Authors' judgement**	**Support for judgement**
Random sequence generation (selection bias)	Unclear risk	Quote: "subjects ... were randomly assigned"
Allocation concealment (selection bias)	Unclear risk	Not stated
Blinding (performance bias and detection bias) All outcomes	Low risk	Quote: "To blind the subjects, staff, and investigators, each subject simultaneously wore three patches during the 6‐day inpatient phase"
Incomplete outcome data (attrition bias) All outcomes	Low risk	Apart from one light smoker dropping out from 44 mg group for nicotine toxicity in week 1, apparently no dropouts.

**Table:** Daughton 1991

***Study characteristics***		
Methods	Study design: parallel RCT Country: USA Recruitment: community volunteers at 2 sites	
Participants	158 smokers (at least 1 pack of cigarettes per day) 47% men, average age 42, average cigarettes per day: 33	
Interventions	1) Nicotine patch (15 cm², 4 weeks) worn for 16 hrs/day 2) Nicotine patch (15 cm², 4 weeks) worn for 24 hrs/day 3) Placebo patch, 4 weeks	
Outcomes	Sustained abstinence at 6 months Validation: CO at 2 to 4 weeks (none after 4 weeks) Adverse events: assessed weekly during treatment (4 weeks)	
Notes	This study was funded by ALZA Corporation, California. Conflicts of interest: 3 of the authors have corporate affiliations or contractual agreements with, or own stock in, ALZA or Merrell Dow.	
***Risk of bias***		
**Bias**	**Authors' judgement**	**Support for judgement**
Random sequence generation (selection bias)	Unclear risk	Quote: "All 158 study‐eligible volunteers were randomly assigned"
Allocation concealment (selection bias)	Unclear risk	Not stated
Blinding (performance bias and detection bias) All outcomes	Low risk	Described as: "double‐blind"; "All of the patches were physically identical in appearance".
Incomplete outcome data (attrition bias) All outcomes	Unclear risk	Dropouts (if any) not reported; included as treatment failures in our analysis; results presented on an ITT basis

**Table:** Dennis 2016

***Study characteristics***		
Methods	Study design: parallel RCT Country: USA Recruitment: from outpatient clinic referrals, and by flyers and letters advertising a study on PTSD and smoking cessation posted in local hospitals	
Participants	63 smokers: diagnosed with PTSD, aged 18 to 70 years, cigarettes per day ≥ 10, willing to quit within the following 30 days 46% men; average age 42; average cigarettes per day: 17.7; mean FTND: 4.1	
Interventions	1) 2 weeks of nicotine patch (preloading) treatment pre‐quit date, followed by 6 weeks of nicotine patch and nicotine gum/lozenge from quit date 2) 2 weeks of placebo patch pre‐quit date, followed by 6 weeks of nicotine patch and nicotine gum/lozenge from quit date Initial patch dose 21 mg/24‐hour – unclear if tapered down and if so at what dose	
Outcomes	30‐day PPA at 6‐month follow‐up Validation: salivary cotinine (< 10 ng/mL) Adverse events: not measured	
Notes	Participants were compensated up to USD 650 for complete participation The study was funded by the National Institutes of Health (R21CA128965; R01CA037220; R34DA038272), by the Department of Veterans Affairs (VA) Office of Research and Development (ORD) Health Services Research and Development Service (HSR&D; I01HX000132; I01HX001109), and by the VA Mid‐Atlantic Mental Illness Research, Education, and Clinical Center Conflicts of interest: none to declare	
***Risk of bias***		
**Bias**	**Authors' judgement**	**Support for judgement**
Random sequence generation (selection bias)	Unclear risk	No detail on exactly how participants were randomised Quote: "randomisation to active nicotine patch or placebo patch was stratified by gender and presence of current MDD [major depressive disorder]".
Allocation concealment (selection bias)	Low risk	Quote: "patch allocation was concealed by maintaining a list through the pharmacy that was unavailable to study investigators and coordinators"
Blinding (performance bias and detection bias) All outcomes	Unclear risk	Quote: “Participants were randomized…in a double blind fashion.” No detail is given on who was blinded and how exactly this occurred, but the control group received placebo patch rather than no patch
Incomplete outcome data (attrition bias) All outcomes	High risk	> 50% participants lost to follow‐up (18/32 in active patch group; 19/31 in placebo patch group), although similar dropout in each group

**Table:** Dignan 2019

***Study characteristics***		
Methods	Study design: 2 x 2 x 2 x 2 incomplete factorial RCT (15 possible treatment combinations) Country: USA Recruitment: self‐referral following promotion via radio, newspaper, flyers at community events, organised activities by American Indian Health organisations, markets, casinos, tribal headquarters, chapter houses. Participants were referred by pharmacists and healthcare providers at one study site.	
Participants	254 American Indian smokers; willing to stop smoking within 3 months from date of recruitment. 36% men, age range: 18 to 80 years (63% under 50 years old), average cigarettes per day: 13, mean FTND: 6	
Interventions	2 x 2 x 2 x 2 incomplete factorial design (15 possible treatment combinations) 1) NRT component: Minimal: 1 NRT product (nicotine patch or fast‐acting oral NRT) Intense: 2 NRT products (nicotine patch and/or fast‐acting oral NRT) 2) Pre‐cessation telephone counselling component: Minimal: 2 counselling sessions Intense: 3 counselling sessions 3) Cessation in‐person counselling component: Minimal: counselling on the quit date plus 2 additional sessions Intense: counselling on the quit date plus 3 additional sessions 4) mHealth intervention component: Minimal: 2 text messages daily Intense: 4 text messages daily Behavioural therapy components 2, 3 and 4 were collapsed for inclusion in meta‐analysis as no interactions between components were detected.	
Outcomes	PPA at 18 months after target quit date Validation: CO monitoring (< 10 ppm) Adverse events: not reported	
Notes	Funding: National Cancer Institute, United States of America, R01CA170336 Conflicts of interest: the authors declare no potential conflicts of interest. This study is new to the 2023 review update.	
***Risk of bias***		
**Bias**	**Authors' judgement**	**Support for judgement**
Random sequence generation (selection bias)	Unclear risk	Quote: "Subjects were assigned at random to one of 15 groups…". No further information provided.
Allocation concealment (selection bias)	Unclear risk	Not specified
Blinding (performance bias and detection bias) All outcomes	High risk	No placebo or blinding
Incomplete outcome data (attrition bias) All outcomes	High risk	Total sample followed‐up at 18 months: 16 participants, < 50% of the total sample

**Table:** Garvey 2000

***Study characteristics***		
Methods	Study design: parallel RCT Country: USA Recruitment: community volunteers	
Participants	608 smokers, aged > 20, smoking > 5 cigarettes per day 49% men, average cigarettes per day: 23	
Interventions	1) 4 mg nicotine gum (recommended 9 to 15 pieces), weaning from 2 months 2) 2 mg nicotine gum, use as described for group 1 3) Placebo gum All received brief counselling (5 to 10 minutes) at each study visit (1, 7, 14, 30 days, 2, 3, 6, 9, 12 months)	
Outcomes	Sustained abstinence at 12 months (relapse defined as 7+ consecutive days or episodes of smoking) Validation: CO ≤ 8 ppm Adverse events: not measured	
Notes	This study was supported by grants DA06183 and DA10073 from the National Institute on Drug Abuse, and by the Department of Veterans Affairs. Conflicts of interest: not reported	
***Risk of bias***		
**Bias**	**Authors' judgement**	**Support for judgement**
Random sequence generation (selection bias)	Unclear risk	Stratified by dependence level (high/low) and then allocated [Quote]: "using a randomized, double‐blind procedure"
Allocation concealment (selection bias)	Unclear risk	No further detail
Blinding (performance bias and detection bias) All outcomes	Unclear risk	Described as double‐blind, but no further information
Incomplete outcome data (attrition bias) All outcomes	Unclear risk	Relapsers were included as failures. Dropout rates not reported

**Table:** Garvey 2006

***Study characteristics***		
Methods	Study design: parallel RCT Country: USA Recruitment: predominantly through newspaper advertisements	
Participants	391 adult smokers; average cigarettes per day: 25	
Interventions	1) Placebo 2) Individualised nicotine replacement transdermal patch: dose at 50% nicotine replacement of baseline level 3) Individualised nicotine replacement transdermal patch: dose at 100% nicotine replacement of baseline level 4) 21 mg nicotine patch 5) 42 mg nicotine patch To reach target replacement levels, patch dose was adjusted if necessary during the first 2 weeks post‐cessation for participants assigned to 100% or 50% replacement (i.e. arms 2 and 3 above).	
Outcomes	Quit rate at 1‐year follow‐up (no further definition provided) Validation: not reported Adverse events: not reported	
Notes	Information extracted from a conference abstract and a secondary publication reporting on unrelated outcomes. Funding: NIDA ‐ DA12165 Conflicts of interest: not reported in conference abstract. Acknowledgements reported in a secondary publication stated: "The authors would like to thank GlaxoSmithKline, Parsippany, New Jersey, for kindly supplying the nicotine (NicoDerm CQ) and placebo patches used in the study. The authors also would like to thank Mary Cooley for assisting in the early conduct phases of the study and Brianna Wadler for editorial help." This study was excluded from previous versions of this review due to insufficient information (Lindson 2019); it was reassessed and deemed eligible and is thus new to this current update version.	
***Risk of bias***		
**Bias**	**Authors' judgement**	**Support for judgement**
Random sequence generation (selection bias)	Unclear risk	Quote: "….prospective, randomized, clinical trial…". No further information provided
Allocation concealment (selection bias)	Unclear risk	Not specified
Blinding (performance bias and detection bias) All outcomes	Unclear risk	Placebo‐controlled but blinding not described
Incomplete outcome data (attrition bias) All outcomes	Unclear risk	Not specified

**Table:** Glavas 2003

***Study characteristics***		
Methods	Study design: parallel RCT Country: Croatia Recruitment: community volunteers	
Participants	160 smokers	
Interventions	1) Nicotine patch, 24‐hour, 25 mg/15 mg/8 mg starting dose depending on baseline cigarettes per day. 6 weeks 2) Nicotine patch, 24‐hour, 25 mg/15 mg starting dose depending on baseline cigarettes per day. 3 weeks 3) Placebo patch. 6 weeks 4) Placebo patch. 3 weeks	
Outcomes	Abstinence at 6 months after EOT (abstinence defined as ≤ 2 cigarettes per week) Validation: CO < 11 ppm Adverse events: monitored during treatment (3 weeks in 1 group and 6 weeks in another)	
Notes	Study funding information not reported Conflicts of interest: not reported Author supplied additional details in personal communication	
***Risk of bias***		
**Bias**	**Authors' judgement**	**Support for judgement**
Random sequence generation (selection bias)	Unclear risk	Method not stated
Allocation concealment (selection bias)	Low risk	Quote: "presealed numbered envelopes"
Blinding (performance bias and detection bias) All outcomes	Unclear risk	Quote: "The envelopes were prepared well in advance and the distribution was commissioned to a nurse not taking part in the evaluation process"
Incomplete outcome data (attrition bias) All outcomes	Unclear risk	Not stated

**Table:** Goldstein 1989

***Study characteristics***		
Methods	Study design: factorial RCT Country: USA Recruitment: community volunteers	
Participants	89 smokers (excluding 18 early treatment dropouts not included in results)	
Interventions	Factorial design of 2 types of group treatment, and 2 schedules for use of nicotine gum. Behaviour therapy arms combined. 1) Fixed‐schedule nicotine gum (2 mg); 1 piece/hour for 1st week with tapering over 10 weeks 2) Ad lib nicotine gum; to be used when urge to smoke, maximum 30/day	
Outcomes	PPA at 6 months Validation: saliva cotinine < 10 ng/mL or CO < 8 ppm for people still using gum Adverse events: not measured	
Notes	Each participant paid USD 130 at start of study, of which they recovered USD 30 for supplying follow‐up information. This study was funded by grant IN‐45Z from the American Cancer Society and by grant HL‐32318 from the National Heart, Lung, and Blood Institute. Conflicts of interest: not reported	
***Risk of bias***		
**Bias**	**Authors' judgement**	**Support for judgement**
Random sequence generation (selection bias)	Unclear risk	Method not stated
Allocation concealment (selection bias)	Unclear risk	Quote: "each subject was assigned"
Blinding (performance bias and detection bias) All outcomes	High risk	Not relevant; placebo gum not used
Incomplete outcome data (attrition bias) All outcomes	Low risk	18 early dropouts (16.8%) not included. Dropout rate by EOT was 7.9%, by 6 months 3.4%; losses included as failures

**Table:** Hall 2009

***Study characteristics***		
Methods	Study design: factorial RCT Country: USA Recruitment: from the general public through advertising, public service announcements and flyers	
Participants	402 smokers; aged ≥ 50 years, ≥ 10 cigarettes per day 59.7% men; average age 56.7 years; average cigarettes per day 20.5; mean FTND 4.8; average years regular smoking 37.8	
Interventions	Factorial 2 x 2 design: extended NRT and extended CBT All participants completed a 12‐week treatment programme that included group counselling, 12 weeks of bupropion and 10 weeks of nicotine gum (beginning on quit day). Participants were asked to taper their gum use down completely by week 12. 1) Standard treatment: participants received no further treatment after week 12 2) Extended NRT: participants were provided with another 40 weeks of nicotine gum from their quit day (a total of 50 weeks of gum treatment). No CBT past 12 weeks 3) Extended CBT: participants received 11 additional CBT sessions between weeks 10 and 52. 10 weeks of NRT 4) Extended NRT and extended CBT: participants received an extra 40 weeks of nicotine gum and an additional 11 CBT sessions following the planned quit day (total 50 weeks gum treatment)	
Outcomes	7‐day PPA at 52 weeks post‐baseline; biochemically validated (CO ≤ 10 ppm and anatabine/anabasine ≤ 2 mg/mL) Other abstinence measures: 7‐day PPA at 12, 24, 64, 104 weeks post‐baseline; biochemically validated (CO ≤ 10 ppm and anatabine/anabasine ≤ 2 mg/mL) Adverse events: measured to week 104 (treatment was to week 50)	
Notes	Factorial trial: authors do not appear to have tested for any interaction between the effects of the 2 interventions tested. However, the review team carried out the same analysis, testing for an interaction at the relevant follow‐up point, and found no statistically significant interaction. As there was no significant interaction between the 2 treatments tested, we combined groups 1 and 3, and groups 2 and 4 for meta‐analysis, so that we could compare 50 weeks extended NRT treatment to 10 weeks 'standard' NRT treatment. Participants were paid USD 25 per completed assessment The study was funded by the National Institute on Drug Abuse (R01 DA02538, K05 DA016752, K23 DA018691 and P50 DA 09253) Conflicts of interest: none	
***Risk of bias***		
**Bias**	**Authors' judgement**	**Support for judgement**
Random sequence generation (selection bias)	Low risk	Quote: “…assigned randomly to one of four experimental conditions using a computerized allocation list by the project statistician (Ms Robbins), who had no contact with participants.”
Allocation concealment (selection bias)	Low risk	As above, plus the following: Quote: “The assignment of individual participants by subject number was then transmitted electronically to clinical staff.”
Blinding (performance bias and detection bias) All outcomes	High risk	No blinding for NRT intervention
Incomplete outcome data (attrition bias) All outcomes	Low risk	> 50% followed up by strictest quit time point. Similar follow‐up between groups

**Table:** Hays 1999

***Study characteristics***		
Methods	Study design: parallel RCT Country: USA (3 sites) Recruitment: community volunteers	
Participants	958 smokers, > 15 cigarettes per day, motivated to quit 50% men, average age 44, typically smoked 21 to 40 cigarettes per day	
Interventions	1) Nicotine patches (22 mg, 24‐hour for 6 weeks) purchased by participants, open‐label 2) Nicotine patches (22 mg, 24‐hour for 6 weeks) provided, double‐blind 3) Placebo patches provided The intervention replicated an over‐the‐counter environment, with no counselling intervention and minimal study recording. Weekly visits required for CO measurement and adverse experience recording, but study sites were not in medical centres and there was no advice, counselling or interaction with medical personnel.	
Outcomes	Abstinence at 6 months (7‐day PPA) Validation: CO ≤ 8 ppm Adverse events: measured for 6 weeks (during the treatment phase)	
Notes	Study was supported by Elan Pharmaceutical Research Corp, Gainesville, GA Conflicts of interest: not reported	
***Risk of bias***		
**Bias**	**Authors' judgement**	**Support for judgement**
Random sequence generation (selection bias)	Low risk	Quote: "Computer‐generated random schedule"
Allocation concealment (selection bias)	Low risk	2‐stage process. 1. random allocation to 1 of 2 trials, i.e. open‐label pay trial or placebo‐controlled. 2. Those in placebo trial were then assigned "by means of a computer‐generated code, in blocks of 20".
Blinding (performance bias and detection bias) All outcomes	Low risk	Quote: "The randomization code was not revealed to any of the investigators until completion of the study." Packaging identical
Incomplete outcome data (attrition bias) All outcomes	Unclear risk	Participants who missed follow‐up visits classified as failures. Dropout rates not reported

**Table:** Herrera 1995

***Study characteristics***		
Methods	Study design: parallel RCT Country: Venezuela Recruitment: community volunteers	
Participants	322 smokers > 10 cigarettes per day, scoring ≥ 4 on FTND, no serious illness. Only those who were ready to quit after 4 weeks of behavioural treatment were randomised. 57% men, average age ~38, average cigarettes per day: 33 for high dependence, 16 for low dependence	
Interventions	Low‐dependence smokers (FTND 4 ‐ 6): 1) 2 mg nicotine gum 2) Placebo gum High‐dependence smokers (FTND 7 ‐ 11): 1) 4 mg nicotine gum plus 2) 2 mg nicotine gum Participants also randomised to starting medication with increasing dose for 1 week before TQD, or to start at full dose on TQD ‐ there was no blinding for this	
Outcomes	Sustained abstinence at 2 years (1 year also reported) Validation: expired CO < 6 ppm Adverse events: measured daily during treatment	
Notes	Relapse between 1 and 2 years similar between low‐dependence groups. Higher relapse in 4 mg high‐dependence group than 2 mg Funding and conflicts of interest not reported	
***Risk of bias***		
**Bias**	**Authors' judgement**	**Support for judgement**
Random sequence generation (selection bias)	Unclear risk	Method not stated
Allocation concealment (selection bias)	Unclear risk	Stratified on dependency scores, to determine dosage. Then "randomly assigned"
Blinding (performance bias and detection bias) All outcomes	Unclear risk	Described as double‐blind
Incomplete outcome data (attrition bias) All outcomes	Unclear risk	68 participants dropped out in phase 1 (weeks 1 to 2) and 10 participants in phase 2 (weeks 4 to 6), i.e. before randomisation. Dropout rates not reported, but classified as relapsed "and not further analyzed"

**Table:** Hilleman 1994

***Study characteristics***		
Methods	Study design: parallel RCT Country: USA Recruitment: community volunteers	
Participants	140 smokers (excluding a buspirone treatment group), smoking > 20 cigarettes per day, FTND ≥ 8 45% men, average age 46, average cigarettes per day 25 to 26	
Interventions	1) Nicotine patch (21 mg/24‐hour) for 6 weeks, no weaning 2) Nicotine patch, 21 mg 4 weeks, weaning to 14 mg 4 weeks, 7 mg 4 weeks	
Outcomes	Abstinence at 6 months Validation: plasma thiocyanate Adverse events: not measured	
Notes	Funding and conflicts of interest not reported	
***Risk of bias***		
**Bias**	**Authors' judgement**	**Support for judgement**
Random sequence generation (selection bias)	Unclear risk	Quote: "open‐label, randomized"
Allocation concealment (selection bias)	Unclear risk	Method not stated
Blinding (performance bias and detection bias) All outcomes	High risk	Open trial with no placebo‐controlled study arm
Incomplete outcome data (attrition bias) All outcomes	Unclear risk	Quote: "The number of patients discontinuing therapy among the three treatment groups was not significantly different"; analyses included all randomised

**Table:** Hughes 1990

***Study characteristics***		
Methods	Study design: parallel RCT Country: USA Recruitment: community volunteers	
Participants	78 smokers, motivated to quit 46% men, average age 34 to 44, average cigarettes per day 24 to 30	
Interventions	1) Placebo gum 2) 1 mg nicotine gum (unbuffered formula, available dose approximately 0.5 mg) 3) 2 mg nicotine gum 4) 4 mg nicotine gum Gum use not recommended for longer than 3 months	
Outcomes	Sustained abstinence at 6 months Validation: independent observer report Adverse events: measured at 1‐week follow‐up (within treatment) using a 13‐item side effects scale. Note: none of the side effects included in the scale are cardiovascular	
Notes	This study was supported by Grants DA‐03728 and DA‐04066 and Research Scientist Development Award DA‐00109 (to JRH) from the National Institute on Drug Abuse. Merrell‐Dow Research Institute provided the drug for the study. Conflicts of interest: not reported	
***Risk of bias***		
**Bias**	**Authors' judgement**	**Support for judgement**
Random sequence generation (selection bias)	Unclear risk	Quote: "Subjects were randomly assigned"
Allocation concealment (selection bias)	Unclear risk	Not stated
Blinding (performance bias and detection bias) All outcomes	Unclear risk	Quote: "in a double‐blind manner"; participants guessed to which group they had been assigned
Incomplete outcome data (attrition bias) All outcomes	Unclear risk	Quote: "Subjects unable to be contacted were counted as smokers". Losses not reported

**Table:** Hughes 1991

***Study characteristics***		
Methods	Study design: parallel RCT Country: USA Recruitment: primary care patients	
Participants	106 smokers, motivation to quit not required 48% men, average age 38, average cigarettes per day 26	
Interventions	1) Free prescription for nicotine gum for up to 6 months 2) Nicotine gum at cost of USD 6/box (96 pieces 2 mg) 3) Nicotine gum at USD 20/box All participants received brief physician advice with 1 follow‐up	
Outcomes	Abstinence at 6 months Validation: observer verification of all 6‐month quitters Adverse events: not measured	
Notes	Tested effect of price on gum use and efficacy. We combined groups 2 and 3 to make 1 purchasing arm in meta‐analysis. Similar quit rates in the 2 combined arms This study was supported by a grant (DA‐04066) and Research Scientist Development Award (DA‐00109) from the National Institute on Drug Abuse. Merrell‐Dow Research Institute provided nicotine gum. Conflicts of interest: not reported	
***Risk of bias***		
**Bias**	**Authors' judgement**	**Support for judgement**
Random sequence generation (selection bias)	Unclear risk	Not stated
Allocation concealment (selection bias)	Low risk	Quote: "Physician opened a sealed envelope" which assigned participants to a price group
Blinding (performance bias and detection bias) All outcomes	High risk	Double‐blind, as described above. But physicians knew how much each participant paid, and therefore which group they were in, so could have managed them differently (Quote: "no anecdotal evidence that this occurred")
Incomplete outcome data (attrition bias) All outcomes	Unclear risk	Losses at 6 months reported; all were counted as failures, but distribution across the groups not reported

**Table:** Hughes 1999

***Study characteristics***		
Methods	Study design: parallel RCT Country: USA (12 sites), Australia (1 site) Recruitment: community volunteers and referrals	
Participants	1039 smokers (≥ 30 cigarettes per day) who had made a prior quit attempt, motivated to try again 50% men, average age 43, average cigarettes per day 38	
Interventions	1) 42 mg nicotine patch (24‐hour, 6 weeks plus 10 weeks tapering) 2) 35 mg nicotine patch 3) 21 mg nicotine patch 4) Placebo patch	
Outcomes	Prolonged abstinence at 6 months (from 2 weeks post‐quit) verified at each follow‐up visit (12‐month follow‐up only completed for 11/13 sites) Validation: CO ≤ 10 ppm Adverse events: measured up to 10 weeks and then at 6‐month and 12‐month follow‐up. Note: measurement at 12 months only occurred at some sites. Treatment duration was to 16 weeks	
Notes	6‐month abstinence rates used in analyses, since not all centres completed 12‐month follow‐up due to sponsor termination of study. Denominators confirmed by author This study was funded by ALZA and Hoechst Marion Roussel. The writing of the study was funded by a Research Scientist Development Award DA‐00109 from the National Institute on Drug Abuse. Conflicts of interest: not reported	
***Risk of bias***		
**Bias**	**Authors' judgement**	**Support for judgement**
Random sequence generation (selection bias)	Unclear risk	Quote: "Subjects were randomly assigned in a double‐blind manner"
Allocation concealment (selection bias)	Unclear risk	Quote: "Subjects were randomly assigned in a double‐blind manner"
Blinding (performance bias and detection bias) All outcomes	Unclear risk	Described as "double‐blind" but no further detail
Incomplete outcome data (attrition bias) All outcomes	High risk	Early termination by sponsor, resulting in incomplete long‐term follow‐up data collection. Losses were included as failures

**Table:** Hughes 2018

***Study characteristics***		
Methods	Study design: parallel RCT Country: USA Recruitment: through internet sites, such as Craigslist, and referral by friends already enrolled	
Participants	701 smokers: aged ≥ 18 years, ≥ 10 cigarettes per day for ≥ 1 year, probably or definitely intend to quit smoking in the next month, no medical caution to use of patch, no use of other nicotine or tobacco products in the last month 43.5% men; average cigarettes per day: 19; FTND: 5.5; average age started smoking: 17.8; any prior quit attempt: 78%	
Interventions	1) Participants advised to 'continue' nicotine patch use in the case of a lapse post‐quit day. Those in the 'continue patch' condition were told: “If you smoke after quitting, continue to use the nicotine patches. Wearing the patches will make it easier for you to return to not smoking. We know that using the patches and smoking a few cigarettes is not harmful. So, if you slip and have a cigarette after quitting, return to not smoking as soon as possible, get rid of any cigarettes you may have, and continue to use the nicotine patches. Do you have any questions or concerns about this?” To minimise adverse events, participants were also told to only use the patch while smoking if they were smoking ≤ 75% of their baseline number of cigarettes per day. 2) Participants advised to 'discontinue' nicotine patch use in the case of a lapse post‐quit day. Those in the 'discontinue patch' condition were told: “If you smoke after quitting, take off your patch for the rest of the day. Using the patches while smoking may give you nicotine levels that are too high, and it's not known if patch use while smoking helps smokers quit. So, if you slip and have a cigarette after quitting, return to not smoking as soon as possible, get rid of any cigarettes you may have, but stop using the patch the day you slip, and resume use on future days only if you completely stop smoking again. Do you have any questions or concerns about this?” For both groups, counsellors delivered the instructions above at least 8 times throughout the interventions, and patches were provided for 10 weeks post‐quit date. For all participants, the behavioural counselling protocol was based on USPHS [United States Public Health Service] Clinical Practice Guidelines that emphasise the provision of social support and problem‐solving around high‐risk‐for‐lapse situations. Counselling was delivered in 6 proactive phone calls that occurred 7 and 3 days before, and 2, 7, 14 and 28 days after participants' designated quit date. The first call lasted about 20 minutes; subsequent calls were 10 to 15 minutes.	
Outcomes	Self‐reported 7‐day PPA smoking abstinence at 6 months post‐quit Other abstinence measures: self‐reported 7‐day PPA at 4 months post‐quit Adverse events: measured to 1 week post‐treatment (12 weeks)	
Notes	The study was funded by the US National Cancer Institute (Grant CA165080) Conflicts of interest: Dr. Hughes has received consulting and speaking fees from several companies that develop or market pharmacological and behavioral treatments for smoking cessation or harm reduction and from several non‐profit organizations that promote tobacco control. He also consults (without payment) to Swedish Match.	
***Risk of bias***		
**Bias**	**Authors' judgement**	**Support for judgement**
Random sequence generation (selection bias)	Low risk	Quote: “The randomization schedule and implementation of randomization was conducted by a statistician who had no contact with participants” Quote: “Treatment condition was based on a stratified block design using the SAS procedure PLAN”
Allocation concealment (selection bias)	Low risk	As above
Blinding (performance bias and detection bias) All outcomes	Low risk	Quote: “Neither participants, research assistants, nor counselors were blind to condition”. However, this is a trial of a behavioural instruction so blinding is impossible. Not biochemically validated, and unknown if participants were aware of the treatment the other group was receiving, but both groups received the same contact. Quote: “We matched the Continue Patch and Discontinue Patch use messages on length and frequency.” Collection of outcomes (detection bias) was blinded as participants completed a survey through a phone line, entering data using the phone keypad.
Incomplete outcome data (attrition bias) All outcomes	Low risk	10% across conditions – reported that this did not differ between groups. 34/321 in 'continue' group did not make a quit attempt and 26/345 in 'discontinue' group ‐ similar between groups

**Table:** Jorenby 1995

***Study characteristics***		
Methods	Study design: factorial RCT Country: USA Recruitment: community volunteers	
Participants	504 adult smokers (≥ 15 cigarettes per day) 47% men, average age 44, average cigarettes per day ~27	
Interventions	1) Nicotine patch 22 mg for 6 weeks then 2 weeks 11 mg with minimal counselling 2) Same patch, individual counselling 3) Same patch, group counselling 4) 44 mg patch for 4 weeks then 2 weeks 22 mg then 2 weeks 11 mg with minimal counselling 5) Same patch, individual counselling 6) Same patch, group counselling	
Outcomes	Abstinence (> 1 week) at 6 months Validation: CO < 10 ppm Adverse events: measured weekly for 8 weeks (during treatment)	
Notes	"This study was sponsored by a grant from Elan Pharmaceutical Research Corporation, Gainesville, Ga. Drs Jorenby, Smith, Fiore, Lewis, and Baker have worked on clinical research studies funded in part by Alza Corporation; Ciba‐Geigy Corporation; Elan Pharmaceutical Research Corporation; Lederle Laboratories; and Marion Merrell Dow, Inc. Drs Hurt, Croghan, and Hays and Mr Offord have worked on clinical research studies funded in part by Lederle Laboratories, Elan Pharmaceutical Research Corporation, BurroughsWellcome, and Kabi. Dr Fiore has received honoraria for educational activities from Ciba‐Geigy Corporation; Elan Pharmaceutical Research Corporation, Lederle Laboratories Division; Marion Merrell Dow, Inc; and Parke‐Davis Conflicts of interest: Dr Hurt has received honoraria for educational activities from Ciba‐Geigy Corporation, Marion Merrell Dow, Inc, and McNeil Pharmaceuticals. Mr Offord has received honoraria for educational activities from Elan Pharmaceutical Research Corporation."	
***Risk of bias***		
**Bias**	**Authors' judgement**	**Support for judgement**
Random sequence generation (selection bias)	Unclear risk	Quote: "randomly assigned"; "All participants were also randomly assigned to one of the three types of counselling"
Allocation concealment (selection bias)	Unclear risk	Quote: "randomly assigned"; "All participants were also randomly assigned to one of the three types of counselling"
Blinding (performance bias and detection bias) All outcomes	Unclear risk	Quote: "in a double‐blind manner" for weeks 1 to 4, then open‐label for weeks 5 to 8
Incomplete outcome data (attrition bias) All outcomes	Low risk	Losses reported, but included as failures

**Table:** Kalman 2006

***Study characteristics***		
Methods	Study design: parallel RCT Country: USA Recruitment: Veterans Admin Medical Centre and community‐based substance abuse treatment facility	
Participants	130 smokers (≥ 20 cigarettes per day with history of alcohol dependence and ≥ 2 month abstinence from alcohol and illicit drugs) 84% men, average age 47, average cigarettes per day: 32	
Interventions	Dose response trial 1) Nicotine patch (42 mg (2 x 21 mg)) 4 weeks, then tapered for 8 weeks 2) Nicotine patch (21 mg and placebo) for 4 weeks then same tapering as group 1	
Outcomes	Abstinence at 36 weeks (26 weeks post‐EOT) (7‐day PPA) Validation: CO < 10 ppm Adverse events: measured during treatment (up to 12 weeks post‐quit date)	
Notes	This study was supported by National Institute on Drug Abuse Research Grant R29‐DA11713‐01. GlaxoSmithKline Beecham provided the nicotine patches for this project. Conflicts of interest: not reported	
***Risk of bias***		
**Bias**	**Authors' judgement**	**Support for judgement**
Random sequence generation (selection bias)	Unclear risk	Not stated
Allocation concealment (selection bias)	Unclear risk	Quote: "[participants] were randomly assigned".
Blinding (performance bias and detection bias) All outcomes	Unclear risk	Double‐blind for 4 weeks, then open‐label dose tapering phase
Incomplete outcome data (attrition bias) All outcomes	Low risk	10 dropped out before treatment, and 4 excluded for protocol violation. Analyses were ITT, with dropouts reported and counted as failures

**Table:** Killen 1990

***Study characteristics***		
Methods	Study design: factorial RCT Country: USA Recruitment: community volunteers who had abstained from smoking for 48 hours	
Participants	1218 adult smokers 48% men, average age 43, average cigarettes per day: 25	
Interventions	1) Nicotine gum (2 mg, 8 weeks) ad lib dosing 2) Nicotine gum on a fixed dose 3) Placebo gum 4) No gum Each group was also factorially randomised to 1 of 3 psychological interventions.	
Outcomes	PPA at 12 months (7‐day PPA) Validation: cotinine, except participants who moved away Adverse events: measured weekly for 8 weeks (during treatment)	
Notes	This study was supported by US Public Health Service grant 5 ROI CA38303 from the National Cancer Institute and by the Merrell Dow Research Institute, Cincinnati Conflicts of interest: not reported	
***Risk of bias***		
**Bias**	**Authors' judgement**	**Support for judgement**
Random sequence generation (selection bias)	Unclear risk	Not stated
Allocation concealment (selection bias)	Unclear risk	Quote: "randomly assigned"
Blinding (performance bias and detection bias) All outcomes	Low risk	Quote: "Assignment to gum condition was double‐blind"
Incomplete outcome data (attrition bias) All outcomes	Low risk	8 deaths removed from final analyses. Participants moving out of the area were removed from the analyses. Unconfirmed claims of abstinence counted as smokers

**Table:** Killen 1999

***Study characteristics***		
Methods	Study design: parallel RCT Country: USA Recruitment: community volunteers responding to advertisements ‐ heavy smokers selected from responders	
Participants	408 heavy smokers (> 25 cigarettes per day) 59% men; average age 47; average cigarettes per day: 36; modified FTND score: 18	
Interventions	1) 25 mg nicotine patch for 6 weeks (16‐hour, no tapering) 2) 15 mg nicotine patch for 6 weeks Self‐help treatment manual, short video showing patch use and placement	
Outcomes	Sustained abstinence at 12 months (7‐day PPA at both 6 and 12 months) Validation: saliva cotinine < 20 ng/mL (not required for 3 individuals not in area) Adverse events: measured at 24 hours, and 1, 2, 4 and 6 weeks (during treatment)	
Notes	85% of self‐reported quitters provided samples for validation at 12 months This study was funded by the US Public Health Service Grant 1 R01 CA 68968 from the National Cancer Institute. Pharmacia and Upjohn AB (Sweden) provided the nicotine patches. Conflicts of interest: not reported	
***Risk of bias***		
**Bias**	**Authors' judgement**	**Support for judgement**
Random sequence generation (selection bias)	Unclear risk	Not stated
Allocation concealment (selection bias)	Unclear risk	Quote: "Smokers ... were randomized"
Blinding (performance bias and detection bias) All outcomes	Low risk	Quote: "Assignment to treatment dose was double‐blind"
Incomplete outcome data (attrition bias) All outcomes	Low risk	Participants leaving the area were excluded from analyses; all other unconfirmed claims of abstinence were counted as failures. Losses fully reported

**Table:** Kornitzer 1987

***Study characteristics***		
Methods	Study design: parallel RCT Country: Belgium Recruitment: worksite primary care clinic	
Participants	199 smokers (average cigarettes per day 24 to 25)	
Interventions	1) Nicotine gum (4 mg) for at least 3 months 2) Nicotine gum (2 mg) for same time period	
Outcomes	PPA at 12 months Validation: cotinine and carboxyhaemoglobin in a subsample of participants Adverse events: not reported	
Notes	Funding and conflicts of interest not reported	
***Risk of bias***		
**Bias**	**Authors' judgement**	**Support for judgement**
Random sequence generation (selection bias)	Unclear risk	Not stated
Allocation concealment (selection bias)	Unclear risk	Quote: "subjects were randomised"
Blinding (performance bias and detection bias) All outcomes	Unclear risk	Quote: "in a double‐blind way"; blinding was broken at 3 months
Incomplete outcome data (attrition bias) All outcomes	Unclear risk	Losses evident in Tables II and IV

**Table:** Kornitzer 1995

***Study characteristics***		
Methods	Study design: parallel RCT Country: Belgium Recruitment: worksite volunteers	
Participants	374 healthy smokers (> 10 cigarettes per day for > 3 years), motivated to quit 61% men, average age 40, average cigarettes per day 25	
Interventions	1) Nicotine patch (12 weeks 15 mg/16‐hour, 6 weeks 10 mg, 6 weeks 5 mg) and nicotine gum (2 mg, as required) 2) Nicotine patch and placebo gum 3) Placebo patch and placebo gum	
Outcomes	Sustained abstinence at 12 months Validation: CO < 10 ppm Adverse events: measured at each visit during treatment (6 months)	
Notes	This study was supported by Pharmacia Consumer Pharma Conflicts of interest: not reported	
***Risk of bias***		
**Bias**	**Authors' judgement**	**Support for judgement**
Random sequence generation (selection bias)	Low risk	See below
Allocation concealment (selection bias)	Low risk	Quote: "randomized list generated by a computer program". Randomisation balanced between companies 2/2/1
Blinding (performance bias and detection bias) All outcomes	Low risk	Quote: "The investigator and the subjects were completely blind concerning treatment" Quote: "unblinding was never requested during the whole study"
Incomplete outcome data (attrition bias) All outcomes	Unclear risk	Withdrawals counted as treatment failures. All analyses conducted on ITT basis. Dropout and withdrawal rates not reported

**Table:** Krupski 2016

***Study characteristics***		
Methods	Study design: parallel RCT Country: USA Recruitment: smokers who contacted the New York stop smoking quit‐line between March 2010 and October 2010	
Participants	3118 smokers; aged ≥ 18 years, ≥ 20 cigarettes per day, 5 or 6 on Heaviness of Smoking Index, interested in using NRT to quit smoking 53% men, mode age range 45 to 54 years, average cigarettes per day not available but a large majority smoked > 30 cigarettes per day, 88% time to first cigarette < 5 minutes	
Interventions	1) 2‐week supply of nicotine patches plus 2‐week supply of nicotine lozenges 2) 2‐week supply of nicotine patches Advice to wear each patch for 24 hours, and to use lozenges consistently (every 1 to 2 hours while awake)	
Outcomes	Self‐reported 30‐day PPA at 7 months Other abstinence measures: self‐reported 7‐day PPA at 7 months Validation: none Adverse events: not measured	
Notes	The study was funded by New York State Smokers’ Quitline (NYS Department of Health) and Roswell Park Cancer Institute Cancer Center Support Grant (NCI grant #P30 CA016056). Conflicts of interest: "Dr. Cummings provides expert testimony in litigation against cigarette manufacturers, provides consulting advice and has received grants from Pfizer, and previously served as a co‐investigator on a multi‐center trial evaluating a nicotine vaccine from Nabi Biopharmaceuticals. Dr. Mahoney has provided expert testimony in litigation against cigarette manufacturers, has received research grants and speaker fees from Pfizer and served as an investigator on a multi‐center trial evaluating the potential efficacy of a nicotine vaccine for cessation sponsored by Nabi Biopharmaceuticals. Dr. Toll has received a grant from Pfizer for medicine only."	
***Risk of bias***		
**Bias**	**Authors' judgement**	**Support for judgement**
Random sequence generation (selection bias)	Unclear risk	No detail on exactly how the randomisation sequence was generated or allocated. Quote: "a randomised experimental design"
Allocation concealment (selection bias)	Unclear risk	As above
Blinding (performance bias and detection bias) All outcomes	High risk	No blinding and no biochemical validation of abstinence
Incomplete outcome data (attrition bias) All outcomes	High risk	Only 41.6% of participants were followed up, but loss to follow‐up was similar between groups (903/1557 in group 1 and 917/1561 in group 2)

**Table:** Kupecz 1996

***Study characteristics***		
Methods	Study design: prospective quasi‐experimental design Country: USA Recruitment: smokers attending for smoking cessation treatment at the Veterans’ Affairs Medical Center in Denver between September 1992 and March 1993 (following self‐enrolment or referral by physician or nurse) were invited to participate	
Participants	45 smokers: motivated to quit 94.7% men; average age: 50.2 years; average FTND: 7; 69% living in a smoking household environment, average pack/year history: 47.2 years	
Interventions	1) Nicotine patch treatment for 10 weeks (21 mg/day for 6 weeks, then 14 mg/day for 2 weeks, then 7 mg/day for 2 weeks) 2) Nicotine gum: 2 mg pieces (chewed for 20 minutes) ad libitum for 12 weeks, then an individualised tapering schedule with the goal of discontinuing therapy within the next 12 weeks All participants began the above treatment on their quit date and attended 4 weekly sessions, which included contract negotiation, positive reinforcement, relaxation exercises, visual imagery and group support. Following the cessation programme, participants attended 7 follow‐up sessions.	
Outcomes	PPA (defined as not smoking at time of asking) 52‐week follow‐up, validated by exhaled CO < 8 ppm Other abstinence measures: PPA at 6, 12 and 26 weeks (CO‐validated) Adverse events: recorded at each session or follow‐up. Note: follow‐up was to 1 year, and treatment was to 24 weeks	
Notes	ITT numbers are not available. There were 7 dropouts after randomisation, but how these were split across study arms is not reported, making it impossible to perform an ITT analysis. There was no response to a request for the numbers randomised. Funding and conflicts of interest not reported	
***Risk of bias***		
**Bias**	**Authors' judgement**	**Support for judgement**
Random sequence generation (selection bias)	High risk	It appears that treatment (gum or patch) was assigned randomly to the month of recruitment and then all participants recruited in that month received the allotted treatment rather than allocating treatment to individual participants. Quote: “A prospective quasi‐experimental design was employed…” Quote: “During this study, patients were assigned to nicotine gum or a nicotine patch on random months.” Quote: “A random number table was used to assign which product would be used. Each month, the nicotine patch or nicotine gum was randomly assigned to participants in that group by blindly selecting the treatment from an envelope that contained both options.”
Allocation concealment (selection bias)	Unclear risk	Quote: “Each month, the nicotine patch or nicotine gum was randomly assigned to participants in that group by blindly selecting the treatment from an envelope that contained both options.” It is unclear whether the treatment for that month was selected before or after the participants had been enrolled for the month. If the treatment was allocated pre‐enrolment, then this could have influenced allocation of individuals.
Blinding (performance bias and detection bias) All outcomes	High risk	Not placebo‐controlled; participants were aware which intervention they were receiving
Incomplete outcome data (attrition bias) All outcomes	Unclear risk	Quote: “Seven dropped out prior to completing the program”

**Table:** LeBlanc 2017

***Study characteristics***		
Methods	Study design: parallel RCT Country: Canada Recruitment: "...from the UOHI Smoking Cessation Clinic and by media advertisements."	
Participants	303 smokers: ≥ 10 cigarettes per day; 18+ years of age; willing to set a date to quit smoking within the 30 days following the baseline assessment; 63.4% men	
Interventions	1) Control group: 10 weeks of declining, standard‐dose, transdermal nicotine patch 2) STEP group: 10 weeks of titrated transdermal nicotine patch (dose based on smoking history) plus ad libitum nicotine inhaler All participants received 5 x 15‐minute counselling sessions from a smoking cessation counsellor. These sessions occurred at 1, 3, 5, 8 and 10 weeks post‐target quit date. Counselling sessions focused on practical counselling (problem‐solving and skills training) and social support.	
Outcomes	Continuous smoking abstinence at 52 weeks' follow‐up Validation: biochemically‐confirmed with a carbon monoxide breath test Other abstinence measures: continuous, biochemically‐confirmed smoking abstinence at 10 and 26 weeks; validated 7‐day PPA at 10, 26 and 52 weeks of follow‐up Adverse events: not reported	
Notes	This study was listed as an ongoing study (NCT01622998) in the previous version of this review. Further information on abstinence outcomes found in a conference abstract allowed this study to be included in the 2023 review update. Limited information could be obtained from abstract (only the risk difference between groups, with an odds ratio and its 95% confidence interval). We contacted study authors for further information but received no response. Funding: not reported Conflicts of interest: not reported	
***Risk of bias***		
**Bias**	**Authors' judgement**	**Support for judgement**
Random sequence generation (selection bias)	Unclear risk	Not specified
Allocation concealment (selection bias)	Unclear risk	Not specified
Blinding (performance bias and detection bias) All outcomes	High risk	Biochemically‐validated abstinence at 52 weeks; no placebo control
Incomplete outcome data (attrition bias) All outcomes	Unclear risk	Not specified

**Table:** Lerman 2004

***Study characteristics***		
Methods	Study design: parallel RCT Country: USA Recruitment: community volunteers and referrals	
Participants	350 smokers (≥ 10 cigarettes per day) (includes 51 who withdrew before treatment) 46% men, average age 46, average cigarettes per day 21	
Interventions	1) Nicotine patch (21 mg/24‐hour) for 8 weeks including tapering 2) Nicotine nasal spray (8 to 40 doses/day, maximum 5/hour) for 8 weeks, tapering over final 4 weeks	
Outcomes	PPA at 6 months (continuous no slips and prolonged lapse‐free unvalidated outcomes also reported) Validation: CO < 10 ppm Adverse events: measured during counselling sessions during treatment (8 weeks)	
Notes	This study was supported by the Transdisciplinary Tobacco Use Research Center grant P5084718 from the National Cancer Institute and the National Institute on Drug Abuse and Public Health Services Research grant M01‐RR0040 from the National Institutes of Health. Dr. Lerman was supported by the Abramson Cancer Center and Annenberg Public Policy Center. Dr. Benowitz was supported by Public Health Services grants DA02277, DA12393, and CA078703, as well as the University of California, San Francisco, Comprehensive Cancer Center. Nicotine nasal spray (Nicotrol) was provided by Pharmacia and Upjohn, Helsingborg, Sweden. Conflicts of interest: consultancies: N. Benowitz (GlaxoSmithKline); grants received: C. Lerman (National Cancer Institute), N. Benowitz (GlaxoSmithKline)	
***Risk of bias***		
**Bias**	**Authors' judgement**	**Support for judgement**
Random sequence generation (selection bias)	Low risk	Quote: "computer‐generated randomization scheme", stratified by study site
Allocation concealment (selection bias)	Low risk	See above
Blinding (performance bias and detection bias) All outcomes	Low risk	Open‐label treatment Outcome assessment quote: "interviewers were blinded to study group assignment"
Incomplete outcome data (attrition bias) All outcomes	Low risk	Dropouts and withdrawals fully tabulated in study publication figure 1. ITT analyses confined to those known to have received treatment, with dropouts included as treatment failures

**Table:** Leung 2019

***Study characteristics***		
Methods	Study design: parallel RCT Country: China Recruitment: referral by doctors and nurses from 20 primary care clinics across Hospital Authority	
Participants	560 smokers; ≥ 10 cigarettes per day for ≥1 year; 85.2% men; average cigarettes per day: 18.6; FTND: 5.71	
Interventions	1) Single therapy (nicotine patch) for 8 weeks plus counselling 2) Combined therapy (nicotine patch and gum ‐ 2 mg every 1 to 2 hours, as required) for 8 weeks plus counselling Patch dose regime mixed based on dependence (number of cigarettes per day before quitting) for both intervention arms: 10 to 19 cigarettes per day: 4 weeks: 14 mg, 4 weeks: 7 mg ≥ 20 cigarettes per day: 4 weeks: 21 mg, 2 weeks: 14 mg, 2 weeks: 7 mg Both groups started treatment on target quit day. Counselling was delivered by registered nurse trained in smoking cessation.	
Outcomes	7‐day PPA at 52 weeks' follow‐up Validation: CO validated (≤ 6 ppm) Other abstinence measures: 7‐day PPA at 4, 12 and 26 weeks follow‐up; CO validated (≤ 6 ppm) Adverse events: cardiac AEs measured up to 1 year	
Notes	Funding: nil Conflicts of interest: the authors declare that they have no competing interests. This study is new to the 2023 review update.	
***Risk of bias***		
**Bias**	**Authors' judgement**	**Support for judgement**
Random sequence generation (selection bias)	Low risk	Quote: "A statistician who was not involved in the statistical analysis independently randomized participants by using a predetermined random table generated by Microsoft Excel 2002."
Allocation concealment (selection bias)	Unclear risk	Quote: "The counsellor who had been concealed from the randomisation and allocation sequence, then assigned the patient to their specified intervention according to the allocated number". No further information given and therefore method of concealment unclear.
Blinding (performance bias and detection bias) All outcomes	High risk	Pharmaceutical intervention with no placebo control group.
Incomplete outcome data (attrition bias) All outcomes	High risk	52‐week retention rate: patch group: 92/286 = 32%; patch and gum group: 88/274 = 32%

**Table:** Moolchan 2005

***Study characteristics***		
Methods	Study design: randomised, 3‐arm trial Country: USA Recruitment: community volunteers	
Participants	120 adolescent smokers (aged 13 to 17) (≥ 10 cigarettes per day), motivated to quit 30% male, average age 15, average cigarettes per day: 19	
Interventions	1) Nicotine patch (21 mg, or 14 mg for < 20 cigarettes per day) for 6 weeks plus placebo gum 2) Nicotine gum (4 mg, or 2 mg for < 24 cigarettes per day) for 6 weeks plus placebo patch 3) Double placebo	
Outcomes	PPA at 6 months Validation: CO and cotinine Adverse events: measured during treatment visits (treatment length 12 weeks)	
Notes	This study was supported by funds from the National Institute on Drug Abuse, Intramural Research Program. GlaxoSmithKline (Research Triangle Park, NC) provided study medications (21‐ and 14‐mg Nicoderm, 2‐ and 4‐mg Nicorette, and placebo patch and gum) Conflicts of interest: none	
***Risk of bias***		
**Bias**	**Authors' judgement**	**Support for judgement**
Random sequence generation (selection bias)	Low risk	Quote: "randomized ... according to an algorithm held by the National Institute on Drug Abuse Pharmacy, with true replacement of the non‐completers"
Allocation concealment (selection bias)	Unclear risk	Not stated
Blinding (performance bias and detection bias) All outcomes	Unclear risk	Described as "double‐blind, double‐dummy", but no further information
Incomplete outcome data (attrition bias) All outcomes	Low risk	Losses to follow‐up were included as failures for cessation. Losses fully reported

**Table:** Paoletti 1996

***Study characteristics***		
Methods	Study design: parallel RCT Country: Italy Recruitment: community volunteers	
Participants	297 smokers (≥ 10 cigarettes per day), motivated to quit Stratified according to baseline cotinine levels 60% men, average age 43, average cigarettes per day 24 in low cotinine group (n = 120), 30 in high group (n = 177)	
Interventions	Stratum A (baseline cotinine < 250 ng/mL) 1) Nicotine patch (15 mg/16‐hour, 18 weeks including taper) 2) Placebo patch Stratum B (baseline cotinine > 250 ng/mL) 3) Nicotine patch 15 mg 4) Nicotine patch 25 mg	
Outcomes	PPA at 12 months Validation: CO and plasma cotinine Adverse events: measured at visits. Note: participants were only asked about particular symptoms (none of which were cardiac)	
Notes	This study was supported by a grant from Pharmacia. Conflicts of interest: AC and FM were recipients of a fellowship at the University of Pisa, sponsored by Pharmacia	
***Risk of bias***		
**Bias**	**Authors' judgement**	**Support for judgement**
Random sequence generation (selection bias)	Unclear risk	Randomisation stratified on plasma cotinine levels. No detail on methods used
Allocation concealment (selection bias)	Unclear risk	Not stated
Blinding (performance bias and detection bias) All outcomes	Unclear risk	Described as double‐blind. All participants got 2 patches, to ensure maintenance of blinding
Incomplete outcome data (attrition bias) All outcomes	Low risk	Losses to follow‐up fully reported

**Table:** Piper 2009

***Study characteristics***		
Methods	Study design: parallel RCT Country: USA Participants: community volunteers	
Participants	1504 smokers motivated to quit 42% men, average age 45, average cigarettes per day: 21.4	
Interventions	1) Nicotine lozenge 2 mg or 4 mg for 12 weeks (based on dose‐for‐dependence level as per instructions) 2) Nicotine patch (24‐hour, 21 mg, 14 mg and 7 mg titrated down over 8‐week period post‐quit) 3) Bupropion slow‐release (SR) (150 mg twice daily, 1 week pre‐quit, 8 weeks post‐quit) 4) Lozenge plus patch (duration and dosage as above) 5) Bupropion plus lozenge (duration and dosage as above) 6) Placebo (5 groups matched to above 5 interventions)	
Outcomes	7‐day PPA at 6 months; initial cessation Validation: CO < 10 ppm Adverse events: measured at study visits during treatment (8 weeks)	
Notes	Analyses conducted using ITT "This study was supported by grant P50 DA019706 from the National Institute on Drug Abuse and by grant M01 RR03186 from the General Clinical Research Centers Program of the National Center for Research Resources. Dr Piper was supported by an Institutional Clinical and Translational Science Award, University of Wisconsin–Madison (KL2 grant 1KL2RR025012‐01). Medication was provided to patients at no cost under a research agreement with GlaxoSmithKline." Conflicts of interest: "Dr Smith has received research support from Elan Corporation. Dr Baker has served as an investigator on research projects sponsored by pharmaceutical companies, including Sanofi‐Synthelabo, Pfizer Inc, and Nabi Biopharmaceuticals. Dr Jorenby has received research support from the National Institute on Drug Abuse, the National Cancer Institute, Pfizer Inc, Sanofi‐Synthelabo, and Nabi Biopharmaceuticals. He has received support for educational activities from the National Institute on Drug Abuse and the Veterans Administration and consulting fees from Nabi Biopharmaceuticals. Dr Fiore has received honoraria from Pfizer. He has served as an investigator on research studies at the University of Wisconsin that were funded by Pfizer, SanofiSynthelabo, GlaxoSmithKlein, and Nabi Biopharmaceuticals. In 1998, the University of Wisconsin appointed Dr Fiore to a named chair funded by an unrestricted gift to University of Wisconsin from Glaxo Wellcome."	
***Risk of bias***		
**Bias**	**Authors' judgement**	**Support for judgement**
Random sequence generation (selection bias)	Unclear risk	Quote: "Randomization was double‐blind and used a block randomization scheme with sex and self‐reported race as the blocking variables."
Allocation concealment (selection bias)	Low risk	Quote: "Staff did not know to which type(s) of medication a participant would be assigned until the moment of randomization, and study staff were blinded to whether the medication was active or placebo."
Blinding (performance bias and detection bias) All outcomes	Unclear risk	Quote: "Double blind." Quote: "Study staff were blinded to whether the medication was active or placebo" (Type of medication (i.e. patch, gum, pill) would have been apparent to both groups).
Incomplete outcome data (attrition bias) All outcomes	Low risk	90 dropouts (out of 1504). Analyses conducted using ITT. Individuals with missing data considered to be smoking

**Table:** Piper 2016

***Study characteristics***		
Methods	Study design: factorial RCT Country: USA Recruitment: smokers attending primary care clinics were invited to participate in a research programme to help them quit smoking	
Participants	637 smokers; aged ≥ 18 years, ≥ 5 cigarettes per day for 6 months, motivated to quit 45.4% men; average age 45.8 years; average cigarettes per day 17.7; mean FTND 4.8; baseline CO 20.3 ppm; HSI 3.1	
Interventions	2 x 2 x 2 x 2 x 2 x 2 factorial design. There were 6 intervention components tested (detailed below) that were tested in different combinations resulting in 32 study groups. 1) Nicotine patches for 3 weeks prior to quit date (patch preloading) versus no preloading patches 2) Nicotine gum for 3 weeks prior to quit date (gum preloading) versus no preloading gum 3) Preparation counselling versus no preparation counselling 4) Intensive cessation in‐person counselling versus minimal in‐person counselling 5) Intensive cessation telephone counselling versus minimal telephone counselling 6) 16 weeks of nicotine patch and gum from quit date versus 8 weeks of nicotine patch and gum from quit date For the purposes of this review, we are interested in comparisons 1, 2 and 6.	
Outcomes	Self‐reported 7‐day PPA at 6‐month post‐quit date Self‐reported 7‐day PPA at 16‐week post‐quit date Validation: none Adverse events: measured in visits at weeks ‐1 and 4, and in calls at weeks 8, 16, and 26	
Notes	This study had a factorial design, and an interaction between interventions was detected. However, results of a regression accounting for this have been presented in the publication and authors supplied group‐by‐group data. We checked to see if the odds ratios generated from these raw data were significantly, clinically different from those generated for the model adjusting for interactions in the paper, for comparisons 1, 2 and 6. Odds ratios were similar in all cases, and in all cases CIs indicated statistically non‐significant results. We have therefore entered raw data, supplied by authors, into meta‐analyses. This results in wider confidence intervals than the models accounting for interactions, but does not affect interpretation. The study was funded by grants 9P50CA143188 and 1K05CA139871 from the National Cancer Institute. Conflicts of interest: "The authors have received no direct or indirect funding from, nor do they have a connection with, the tobacco, alcohol, pharmaceutical or gaming industries or anybody substantially funded by one of these organizations. W.‐Y.L. is partially supported by a grant from Eli Lilly and Company for research that is unrelated to smoking or tobacco dependence treatment."	
***Risk of bias***		
**Bias**	**Authors' judgement**	**Support for judgement**
Random sequence generation (selection bias)	Low risk	Quote: “Participants were randomized to treatment conditions via a database that used stratified permuted block randomization”
Allocation concealment (selection bias)	Low risk	Quote: “Staff were blinded to randomization until eligibility was confirmed; participants were blinded until consent was provided.”
Blinding (performance bias and detection bias) All outcomes	High risk	No placebos. Quote: “assessed by staff who were not involved in treatment, but were not blind to treatment assignment”
Incomplete outcome data (attrition bias) All outcomes	Low risk	Loss to follow‐up < 50% overall (263/637), and similar for each of 6 study comparisons

**Table:** Preloading Investigators 2018

***Study characteristics***		
Methods	Study design: parallel RCT Country: UK Recruitment: by general practice surgeries and a National Health Service (NHS) smoking cessation clinic	
Participants	1792 smokers: aged ≥ 18 years, motivated to quit, suitable for nicotine preloading treatment (evidenced by an addiction to smoking) 52.6% men; average age 48.9; average cigarettes per day 18.9; mean FTND 5.2; mean CO 23.7 ppm; mean longest previous abstinence 400.3 days; cessation support in last 6 months 32.5%	
Interventions	1) Nicotine patch for 4 weeks before quit date (nicotine preloading) 2) No nicotine patch before quit date All participants received usual care from stop‐smoking services, including pharmacotherapy, beginning 1 to 2 weeks before their quit date	
Outcomes	Prolonged abstinence at 12 months post‐quit, biochemically validated (CO < 10 ppm ‐ salivary cotinine or anabasine were measured instead in a minority of cases, where participants could not attend in person for validation) Other abstinence measures: 7‐day PPA at 4 weeks, 6 months and 12 months Prolonged abstinence at 4 weeks and 6 months Adverse events: measured to 1 week post‐quit (1 week post‐cessation of preloading)	
Notes	Participants received payment for travel and inconvenience at 1‐week, 6‐month and 12‐month follow‐up. The study was funded by the National Institute for Health and Care Research (NIHR), Health Technology Assessment programme 09/110/01. The nicotine patches for pre‐quit treatment were provided free of charge by GSK. Conflicts of interest: Paul Aveyard is an NIHR senior investigator and is funded by NIHR Biomedical Research Centre and CLAHRC, Oxford. Peter Hajek and Hayden McRobbie have done consultancy for manufacturers of smoking cessation treatments and investigator‐initiated research funded by a manufacturer of smoking cessation medication. No authors have financial relationships with any organisation that may have a financial interest in the submitted work in the previous three years and no relationships or activities that could have influenced the submitted work.	
***Risk of bias***		
**Bias**	**Authors' judgement**	**Support for judgement**
Random sequence generation (selection bias)	Low risk	Quote: “An independent statistician used Stata to generate a randomisation list…” Quote: "Participants shall be randomized to a treatment arm at their baseline visit. They will be randomized to the intervention or control (1:1 ratio) on the basis of a computer‐generated allocation sequence via the internet, with telephone backup, which will be provided by our electronic Primary Care Research Network (ePCRN)." Quote: "For very rare occasions when access to the network, and therefore database randomization is not available, we will have a backup process involving sequentially numbered, opaque, sealed envelopes for randomization."
Allocation concealment (selection bias)	Low risk	As above
Blinding (performance bias and detection bias) All outcomes	High risk	No blinding Quote: “open label trial so participants, research staff, and NHS Stop Smoking Service personnel knew the arm to which participants were assigned.” Due to UK clinical guidelines in place at the time of the study, stop smoking services were less likely to prescribe varenicline to people in the intervention arm post‐quit than the control arm. Authors tested whether this difference between trial arms affected the study effect size and found that it did. As we have used raw data for our NRT preloading meta‐analysis and cannot control for this, we deem this to be a high bias risk. Groups received different common behavioural support initially. However, the behavioural support in the control arm was designed to reduce bias by offering the same intensity of support in the absence of a placebo. It is not possible to know whether this behavioural support was suitably matched, and therefore whether it was successful in minimising bias.
Incomplete outcome data (attrition bias) All outcomes	Low risk	> 50% followed up at strictest quit time point. Similar attrition between groups (210/899 in group 1 (preloading) and 193/893 in group 2)

**Table:** Puska 1995

***Study characteristics***		
Methods	Study design: parallel RCT Country: Finland Recruitment: community volunteers	
Participants	300 volunteers aged 20 to 65, smoking > 10 cigarettes per day for > 3 years, no serious illness	
Interventions	1) Nicotine patch (15 mg/16‐hours, 12 weeks plus 6 weeks taper) plus nicotine gum (2 mg at least 4 daily) 2) Placebo patch plus nicotine gum (same regimen)	
Outcomes	Sustained abstinence at 12 months Validation: expired CO < 10 ppm Adverse events: measured at all study visits during treatment (treatment length 52 weeks)	
Notes	Funding and conflicts of interest not reported. However, 2 authors are affiliated with Pharmacia Consumer Pharma, Department of Clinical Research	
***Risk of bias***		
**Bias**	**Authors' judgement**	**Support for judgement**
Random sequence generation (selection bias)	Unclear risk	Quote: "The subjects were randomly allocated"
Allocation concealment (selection bias)	Unclear risk	Not stated
Blinding (performance bias and detection bias) All outcomes	Unclear risk	Quote: "The study was carried out in a strictly double blind fashion"
Incomplete outcome data (attrition bias) All outcomes	Low risk	Losses to follow‐up fully reported

**Table:** Rey 2009

***Study characteristics***		
Methods	Study design: parallel RCT Country: Switzerland Recruitment: from smokers attending an academic outpatients clinic (Department of Ambulatory Care and Community Medicine) in Western Switzerland (Lausanne)	
Participants	50 smokers: highly dependent on smoking, defined as smoking ≥ 20 cigarettes per day and/or within 30 minutes of waking 72% men; average age: 40.5 years; average cigarettes per day: 29.9; average exhaled CO: 41.5 ppm; average years of consumption: 20.5 years; average previous quit attempts: 2.7	
Interventions	1) Nicotine nasal spray ‐ advised to use spray when a craving appeared, but to also ensure using 2 puffs an hour 2) Nicotine nasal spray ‐ advised to use spray when craving appeared only Both groups advised to use spray for 2 months from quit date and to reduce use in the second month if tolerable	
Outcomes	Continuous smoking abstinence at 6‐month follow‐up (defined as from the beginning of nasal spray use to the end of the 6th month, occasional slips < 1 cigarettes per day tolerated) Validation: CO ≤ 10 ppm Adverse events: not measured	
Notes	Despite differing usage instructions, study arms used similar amounts of the spray: group 1 used the spray an average of 2.6 (95% CI −2.7 to 7.9) more doses/day compared to group 2 Pharmacia, Switzerland provided free nicotine nasal spray to the participants. They were not involved in data collection, the analysis of the results, in writing or correcting the manuscript, or in deciding whether the paper should be published or not. No further information provided on study funding Conflicts of interest: none	
***Risk of bias***		
**Bias**	**Authors' judgement**	**Support for judgement**
Random sequence generation (selection bias)	Low risk	Quote: "Prior to data collection, a pharmacist prepared a randomization list of 50 blinded shuffled paper slips including 25 As and 25 Bs which were used to assign patients to treatment groups. Each paper slip was sealed in an opaque numbered envelope. Once a patient was included in the study and baseline data was collected, the sealed envelope was opened by the investigator to reveal the patient's allocation."
Allocation concealment (selection bias)	Low risk	As above
Blinding (performance bias and detection bias) All outcomes	Low risk	Investigators employed as much blinding as was feasible Quote: "patients were blinded to the other intervention but were aware of their own. Investigator could not be blinded, as he was to give instructions on the use of NNS [nicotine nasal spray]. During follow‐up, the research nurse was not expressively made aware of the allocation but made all patients aware of the importance of using the spray when craving appeared. Statistician was blinded to which group received which intervention until the end of the analysis."
Incomplete outcome data (attrition bias) All outcomes	Low risk	24/25 participants followed up in group 1 and 25/25 in group 2. Attrition < 50% and similar in both groups

**Table:** Rose 1994

***Study characteristics***		
Methods	Study design: factorial RCT Country: USA Recruitment: community volunteers	
Participants	48 smokers (≥ 20 cigarettes per day) 40% men, average age 34, average cigarettes per day 27 to 29	
Interventions	2 x 2 factorial trial. Mecamylamine arms combined. 1) Nicotine patch (21 mg/24‐hour for 2 weeks before TQD) 2) Placebo After TQD, both groups received active patch for 6 weeks, counselling at clinic visits and self‐help materials	
Outcomes	Sustained abstinence at 12 months Validation: CO ≤ 8 ppm Adverse events: measured at visits until 1 week post‐treatment	
Notes	This study was supported by grant PBR‐61 from the American Cancer Society (Atlanta, GA); by grant DA 02665 from the National Institute on Drug Abuse (Rockville, MD), and by the Medical Research Service of the Department of Veterans Affairs (Washington, DC). Conflicts of interest: not reported	
***Risk of bias***		
**Bias**	**Authors' judgement**	**Support for judgement**
Random sequence generation (selection bias)	Unclear risk	Not stated
Allocation concealment (selection bias)	Unclear risk	Not stated
Blinding (performance bias and detection bias) All outcomes	Unclear risk	Assessment of blinding indicated higher‐than‐chance participant awareness of treatment regimen
Incomplete outcome data (attrition bias) All outcomes	Low risk	Dropout rate reported (low)

**Table:** Rose 1998

***Study characteristics***		
Methods	Study design: factorial RCT Country: USA Recruitment: community volunteers	
Participants	80 smokers (≥ 20 cigarettes per day) 51% men, average age 41, average cigarettes per day: 30	
Interventions	2 x 2 factorial trial. Mecamylamine pretreatment arms combined 1) Nicotine patch (21 mg/24‐hour for 4 weeks before TQD) 2) Placebo After TQD, both groups received active patch and mecamylamine for 6 weeks, counselling at clinic visits and self‐help materials	
Outcomes	Sustained abstinence at 6 months Validation: CO ≤ 8 ppm Adverse events: measured at visits during treatment	
Notes	This study was supported by Grant PBR‐61 from the American Cancer Society and conducted with the assistance of the Medical Research Service of the Department of Veterans Affairs. Conflicts of interest: Jed E. Rose is a patent holder of the nicotine—mecamylamine combination treatment tested in this study	
***Risk of bias***		
**Bias**	**Authors' judgement**	**Support for judgement**
Random sequence generation (selection bias)	Unclear risk	Quote: "participants were randomly assigned"
Allocation concealment (selection bias)	Unclear risk	Not stated
Blinding (performance bias and detection bias) All outcomes	High risk	Placebo patches not used, but participants were blinded to mecamylamine
Incomplete outcome data (attrition bias) All outcomes	Unclear risk	Early dropouts (up to 4 weeks pre‐cessation) reported, but not long‐term

**Table:** Rose 2006

***Study characteristics***		
Methods	Study design: factorial RCT Country: USA Recruitment: community volunteers	
Participants	96 smokers (≥ 20 cigarettes per day), motivated to quit 47% men, average age 45, average cigarettes per day: 29	
Interventions	2 x 3 x 3 factorial trial ‐ only pre‐cessation patch condition contributes to meta‐analysis, other conditions combined. 1) Nicotine patch (21 mg/24‐hour for 2 weeks before TQD) 2) Placebo All participants received mecamylamine 2.5 mg twice a day for 4 weeks post‐TQD, and either 0 mg, 21 mg or 42 mg patch	
Outcomes	PPA at 6 months Validation: CO ≤ 8 ppm Adverse events: not measured	
Notes	Post‐quit conditions did not affect cessation, data not reported in paper This study was supported by grant DA 02665 from the National Institute on Drug Abuse. Conflicts of interest: Dr. Rose is an inventor named on several patent applications dealing with nicotine skin patch and combination nicotine/mecamylamine treatment, and receives royalties from sales of certain nicotine patches. Dr. Rose receives research funding from Phillip Morris USA, Inc.	
***Risk of bias***		
**Bias**	**Authors' judgement**	**Support for judgement**
Random sequence generation (selection bias)	Unclear risk	Not stated
Allocation concealment (selection bias)	Unclear risk	Not stated
Blinding (performance bias and detection bias) All outcomes	Unclear risk	Patch assignment was blinded, but not cigarette type. After quit date, all participants received mecamylamine
Incomplete outcome data (attrition bias) All outcomes	Unclear risk	8.3% of participants dropped out before TQD, and were excluded from analyses

**Table:** Rose 2009

***Study characteristics***		
Methods	Study design: factorial RCT Country: USA Recruitment: community volunteers	
Participants	379 participants, smoking > 15 cigarettes per day for ≥ 3 years, motivated to quit 43% men, average age 42, average cigarettes per day 23, average FTND 6	
Interventions	1) Usual brand of cigarette plus 21 mg/24‐hour patch for 2 weeks pre‐quit 2) Usual brand of cigarette plus placebo patch for 2 weeks pre‐quit 3) Low tar and nicotine cigarette plus 21 mg/24‐hour patch for 2 weeks pre‐quit 4) Low tar and nicotine cigarette plus placebo patch for 2 weeks pre‐quit All groups received same treatment post‐quit: 6 weeks 21 mg/24‐hour patch, following 2 weeks 14 mg/24‐hour patch, remaining 2 weeks 7 mg/24‐hour patch	
Outcomes	Continuous abstinence at 6 months Validation: CO ≤ 8 ppm Adverse events: not measured	
Notes	Treatment had greater effect for those with low FTND Funding provided through grant to Duke University by Philip Morris, USA Conflicts of interest: Dr. Rose has received royalties from sales of certain nicotine patches and is named as inventor on nicotine skin patch patents that expired in 2008.	
***Risk of bias***		
**Bias**	**Authors' judgement**	**Support for judgement**
Random sequence generation (selection bias)	Unclear risk	Quote: "a total of 400 subjects were randomly assigned to one of four treatment groups"
Allocation concealment (selection bias)	Unclear risk	Not specified
Blinding (performance bias and detection bias) All outcomes	Low risk	Quote: "two members of the study team…placed the required number of active or placebo patches into individual plastic bags labelled with subject number and session number… In order to maintain blinding, these members of the study team did not interact with study participants."
Incomplete outcome data (attrition bias) All outcomes	High risk	High number lost to follow‐up (169/379)

**Table:** Rose 2010

***Study characteristics***		
Methods	Study design: parallel RCT Country: USA Recruitment: community volunteers	
Participants	479 smokers of ≥ 10 cigarettes per day, motivated to quit 43% men, average age 44, average cigarettes per day: 24	
Interventions	1) Nicotine patch, 21 mg group: weeks 1 to 7 21 mg/24‐hour (1 active 21 mg/24‐hour patch, 1 placebo patch) 2) Nicotine patch, 42 mg group: weeks 1 to 7 42 mg/24‐hour (2 active 21 mg/24‐hour patches) TQD set at 2 weeks. Weeks 7 to 12: all participants received same NRT dose (weeks 7 to 8: 21 mg/24‐hour patch; weeks 9 to 10: 14 mg/24‐hour patch; weeks 11 to 12: 7 mg/24‐hour patch) All participants provided with denicotinised cigarettes during 2‐week pre‐cessation period to minimise adverse effects of high‐dose NRT	
Outcomes	PPA at 6 months Validation: CO ≤ 10 ppm Adverse events: measured during treatment (treatment length 12 weeks)	
Notes	Primarily a study of effects of genotype on smoking cessation Number of successful quitters at 6 months obtained through communication with author Participants with difficulty sleeping instructed to remove patch at bedtime and apply new ones when they awoke. Participants with other symptoms of nicotine toxicity instructed to reduce dose This study was supported by the National Institutes of Health (NIH) – Intramural Research Program, National Institute on Drug Abuse, Department of Health and Social Services (GR Uhl); a grant to Duke University (Principal Investigator, JE Rose) from Philip Morris USA, Richmond, VA, USA Conflicts of interest: GR Uhl and JE Rose are listed as inventors for a patent application filed by Duke University based on genomic markers that distinguish successful quitters from unsuccessful quitters in data from other clinical trials.	
***Risk of bias***		
**Bias**	**Authors' judgement**	**Support for judgement**
Random sequence generation (selection bias)	Unclear risk	Quote: "Randomized", but method not specified
Allocation concealment (selection bias)	Unclear risk	Not specified
Blinding (performance bias and detection bias) All outcomes	Unclear risk	Placebo used, method of blinding not described
Incomplete outcome data (attrition bias) All outcomes	Unclear risk	197 lost to follow‐up before 10 weeks (not known how many lost at 6 months); similar numbers across groups; participants lost to follow‐up counted as smokers

**Table:** Schlam 2016

***Study characteristics***		
Methods	Study design: factorial RCT Country: USA Recruitment: smokers attending primary care clinics were invited to participate in a research programme to help them quit smoking. Electronic health record technology prompted clinic staff to invite smokers to participate	
Participants	544 smokers; aged ≥ 18 years, ≥ 5 cigarettes per day for 6 months, motivated to quit 41% men, average age 46.2, average cigarettes per day 18.6, mean FTND 4.9, HSI 3.2, baseline CO 18.5 ppm	
Interventions	2 x 2 x 2 x 2 x 2 factorial design. There were 5 intervention components tested (detailed below) that were tested in different combinations resulting in 32 study groups. 1) Nicotine patches and gum for 8 weeks starting on quit date versus nicotine patches and gum for 26 weeks starting on quit date 2) Maintenance counselling versus no maintenance counselling 3) Medication adherence counselling versus no medication adherence counselling 4) Automated adherence calls versus no adherence calls 5) Helping Hand medication dispenser with feedback and counselling versus no medication dispenser, feedback or related counselling For the purposes of this review, we are only interested in comparison 1.	
Outcomes	Self‐reported 7‐day PPA at 52 weeks post‐quit date Validation: none Other abstinence measures: self‐reported 7‐day PPA at 26 weeks post‐quit date Adverse events: measured at 1, 4 and 8 weeks by completed assessments with case managers (and at 16 weeks if receiving extended medication). Also measured at weeks 16, 26, 39 and 52 during follow‐up calls with assessors	
Notes	The study was funded by grants 9P50CA143188 and 1K05CA139871 from the National Cancer Institute. Conflicts of interest: "The authors have received no direct or indirect funding from, nor do they have a connection with, the tobacco, alcohol, pharmaceutical or gaming industries or anybody funded substantially by one of these organizations. W.‐Y.L. is supported partially by a grant from Eli Lilly and Company for research that is unrelated to smoking or tobacco dependence treatment." This study has a factorial design and statistical interactions between factors were reported in the paper. Authors supplied group‐by‐group data. We checked to see if the odds ratios generated from these raw data were significantly, clinically different from those generated for the regression model adjusting for interactions in the paper, for comparison 1. The odds ratios were similar, but the wider confidence intervals generated from the raw data changed the interpretation of the results. The analysis accounting for interactions in the paper resulted in a significant effect of 26‐week gum, but this effect was found to be non‐significant when using raw data from the authors. We therefore have not entered raw data, supplied by authors, into any analysis. We have reported this study narratively in the main text.	
***Risk of bias***		
**Bias**	**Authors' judgement**	**Support for judgement**
Random sequence generation (selection bias)	Low risk	Quote: “Participants were randomized to one of 32 unique experimental conditions… via a database that used stratified, computer‐generated, permuted block randomization…”
Allocation concealment (selection bias)	Low risk	Quote: “Staff could not view the allocation sequence. The database did not reveal participants’ treatment condition to staff until participants’ eligibility was confirmed; participants were blinded to treatment condition until they provided consent.”
Blinding (performance bias and detection bias) All outcomes	High risk	No placebo used, therefore participants were not blinded to treatment condition. Assessors were not involved in treatment but were not blinded to treatment assignment
Incomplete outcome data (attrition bias) All outcomes	Low risk	Attrition: 127/275 extended NRT, 129/269 standard NRT. < 50%, similar in both groups

**Table:** Schnoll 2010a

***Study characteristics***		
Methods	Study design: parallel RCT Country: USA Recruitment: community volunteers	
Participants	575 adult smokers of > 10 cigarettes per day for > 1 year, motivated to quit 53% men, average age 48, average cigarettes per day 21.1, average FTND 5.3	
Interventions	1) 21 mg/24‐hour patch for 24 weeks 2) 21 mg/24‐hour patch for 8 weeks, followed by 16 weeks of placebo patch	
Outcomes	7‐day PPA at 12 months (also reported for 24 weeks) Validation: CO ≤ 10 ppm Adverse events: measured throughout treatment (24 weeks), and also at 52‐week follow‐up	
Notes	This study was supported by a Transdisciplinary Tobacco Use Research Center Grant from the National Cancer Institute and the National Institute on Drug Abuse (P50 CA/DA84718 and P50 CA143187). Conflicts of interest: Dr. Lerman has served as a consultant to GlaxoSmithKline, one company that manufactures the nicotine patch. She has also served as a consultant or has received research funding from AstraZeneca, Pfizer, and Novartis.	
***Risk of bias***		
**Bias**	**Authors' judgement**	**Support for judgement**
Random sequence generation (selection bias)	Low risk	Quote: "computer‐based randomization table"
Allocation concealment (selection bias)	Unclear risk	Not specified
Blinding (performance bias and detection bias) All outcomes	Low risk	Quote: "supply of patches was prepackaged and coded with participant information. The computer program linked the randomization to the patch supply, and only the database manager could link identification with treatment allocation."
Incomplete outcome data (attrition bias) All outcomes	Low risk	Dropouts included as smokers in outcome data. Similar number of dropouts in both groups

**Table:** Schnoll 2010b

***Study characteristics***		
Methods	Study design: parallel RCT Country: USA Recruitment: community volunteers and physician referrals	
Participants	642 treatment‐seeking smokers smoking ≥ 10 cigarettes per day 43% men, average age 45, average cigarettes per day 20.3, average FTND 5.1; average years smoking 26.7	
Interventions	Direct comparison of patch versus lozenge 1) Patch: 21 mg/day for first 6 weeks, 14 mg/day for weeks 7 and 8, 7 mg/day for weeks 9 to 12 2) Lozenge: 4 mg for participants who smoked first cigarette of day within 30 minutes of waking; 2 mg for all other participants. Asked to use 9/day for first 6 weeks, 5/day for weeks 7 to 9, 3/day for weeks 10 to 12	
Outcomes	24‐hour PPA at 6 months Validation: CO ≤ 10 ppm Adverse events: measured at end of treatment (12 weeks) and at 6‐month follow‐up	
Notes	This study was supported by grant RSGPB‐05‐240‐01‐CPPB to Dr. Schnoll from the American Cancer Society and National Institutes of Health grant U10 101178 to Dr. Paul Engstrom. This work was also supported in part by grants: P50 CA143187, R01 CA126969, R01 DA025078, and R21 DA026889. Conflicts of interest: "Dr. Ferris has received grant funding through his institution to conduct research trials for GSK and Novartis during the past 3 years".	
***Risk of bias***		
**Bias**	**Authors' judgement**	**Support for judgement**
Random sequence generation (selection bias)	Unclear risk	Quote: "Randomization was coordinated by Fox Chase Cancer Center and was stratified at each site."
Allocation concealment (selection bias)	Unclear risk	Not specified
Blinding (performance bias and detection bias) All outcomes	High risk	Open‐label trial and although both treatments were active, 2/3 participants had preference for patch
Incomplete outcome data (attrition bias) All outcomes	Low risk	46% loss to follow‐up by 6 months, similar between groups. Missing data reported as smokers

**Table:** Schnoll 2015

***Study characteristics***		
Methods	Study design: parallel RCT Country: USA Recruitment: through 2 universities, by media advertisements. Eligible participants identified through initial telephone screening and in‐person evaluation	
Participants	525 smokers; aged ≥ 18 years, ≥ 10 cigarettes per day, interested in smoking cessation 49.3% men, average age 46.4, average cigarettes per day 17.1, mean FTND 5.1	
Interventions	1) Nicotine patch (21 mg) for 8 weeks from target quit date 2) Nicotine patch (21 mg) for 24 weeks from target quit date 3) Nicotine patch (21 mg) for 52 weeks from target quit date	
Outcomes	7‐day PPA at 12 months Other: 7‐day PPA at 24 weeks Validation: expired CO ≤ 10 ppm Adverse events: measured at 4, 12 and 30 weeks	
Notes	Funding by grants R01 DA025078 and R01 DA033681 from the National Institute on Drug Abuse and grants R01 CA165001 and P50 CA143187 from the National Cancer Institute. Conflicts of interest: Drs Schnoll and Hitsman report receiving varenicline (Chantix) and placebo free of charge from Pfizer for use in ongoing National Institutes of Health–supported clinical trials. Dr Schnoll also reports having provided consultation to Pfizer and GlaxoSmithKline. Results for each individual study arm were requested from and shared by the authors.	
***Risk of bias***		
**Bias**	**Authors' judgement**	**Support for judgement**
Random sequence generation (selection bias)	Low risk	Quote: "The statistician (E.P.W.), independently of participants, provided a computerized randomization scheme, which was stratified by site and used permuted blocks of random‐sized numbers"
Allocation concealment (selection bias)	Unclear risk	Insufficient information
Blinding (performance bias and detection bias) All outcomes	High risk	No blinding
Incomplete outcome data (attrition bias) All outcomes	Low risk	40% lost to follow‐up at 12 months (47% in 8‐week group; 35% in 24‐week group; 38% in 52‐week group). Therefore > 50% followed up overall and no large difference (≥ 20%) between groups

**Table:** Schuurmans 2004

***Study characteristics***		
Methods	Study design: parallel RCT Country: South Africa Recruitment: community volunteers	
Participants	200 smokers 56% men, average age 43, average cigarettes per day: 23 to 26	
Interventions	1) Pretreatment with nicotine patch for 2 weeks prior to quit date. Then active patch (15 mg) for 12 weeks including weaning. 4 sessions of counselling over 10 weeks 2) Pretreatment with placebo patch. Then active patch as for group 1	
Outcomes	Sustained abstinence at 6 months Validation: CO < 10 ppm at each visit Adverse events: measured at all follow‐up visits to 6 months (treatment duration 12 weeks)	
Notes	This study was supported by a grant from the Swiss Science Foundation (MMS). Conflicts of interest: Pfizer provided medication and support with data analysis.	
***Risk of bias***		
**Bias**	**Authors' judgement**	**Support for judgement**
Random sequence generation (selection bias)	Low risk	Quote: "a computer‐generated list"
Allocation concealment (selection bias)	Low risk	Quote: "Numbering of identical boxes containing patches was carried out prior to the study by a person not involved in the study"
Blinding (performance bias and detection bias) All outcomes	Low risk	Quote: "The treatment code was broken only after the last follow‐up visit had been completed and the data recorded"
Incomplete outcome data (attrition bias) All outcomes	Low risk	Dropouts fully recorded at all stages, ITT analyses used and participants lost to follow‐up counted as smokers

**Table:** Smith 2009

***Study characteristics***		
Methods	Study design: parallel RCT Country: USA Recruitment: primary care (12 clinics)	
Participants	1346 smokers of > 10 cigarettes per day for past 6 months 44% men, average age 44, average cigarettes per day 20.3, motivated to quit	
Interventions	1) Bupropion only (up‐titrated during week pre‐quitting, 150 mg twice a day for 8 weeks post‐quit) 2) Nicotine lozenge only (4 mg lozenge if first cigarette of day smoked > 30 minutes after waking, 2 mg otherwise. 1 lozenge every 1 to 2 hours post‐quit weeks 1 to 6; 1 lozenge every 2 to 4 hours weeks 7 to 9; 1 lozenge every 4 to 8 hrs weeks 10 to 12) 3) Nicotine patch only (21 mg post‐quit weeks 1 to 4; 14 mg weeks 5 to 6; 7 mg weeks 7 to 8) 4) Bupropion and lozenge (dosage as above) 5) Patch and lozenge (dosage as above)	
Outcomes	7‐day PPA at 6 months and number of days to relapse Validation: none Adverse events: not measured	
Notes	Analyses completed on ITT basis This study was supported by National Institutes of Health grant 5P50DA019706 (Dr Baker) from the National Institute on Drug Abuse and grant 1K05CA139871 (Dr Baker) from the National Cancer Institute. Dr Piper was supported by an Institutional Clinical and Translational Science Award (UW‐Madison; KL2 grant 1KL2RR025012‐01). Medication was provided to patients at no cost under a research agreement with GlaxoSmithKline. Conflicts of interest: "Dr Smith has received research support from Elan Corporation plc. Dr Jorenby has received research support from Pfizer Inc, SanofiSynthelabo, and Nabi Biopharmaceuticals and has received consulting fees from Nabi Biopharmaceuticals. Dr Fiore has received honoraria from Pfizer Inc and has served as an investigator on research studies at the University of Wisconsin that were funded by Pfizer Inc, SanofiSynthelabo, and Nabi Biopharmaceuticals. In 1998, the University of Wisconsin (UW) appointed Dr Fiore to a named Chair funded by an unrestricted gift to UW from Glaxo Wellcome. Dr Baker has served as an investigator on research projects sponsored by pharmaceutical companies including Sanofi‐Synthelabo, Pfizer Inc, and Nabi Biopharmaceuticals."	
***Risk of bias***		
**Bias**	**Authors' judgement**	**Support for judgement**
Random sequence generation (selection bias)	Unclear risk	Quote: "Smokers were randomized to the 5 treatment conditions within each clinic with blocking on sex and self‐identified race."
Allocation concealment (selection bias)	Unclear risk	Not specified
Blinding (performance bias and detection bias) All outcomes	High risk	Open‐label
Incomplete outcome data (attrition bias) All outcomes	Low risk	158 individuals who did not pick up study medication at first point not included in analyses; 122 withdrawals and 9 deaths considered to be smoking

**Table:** Smith 2013

***Study characteristics***		
Methods	Study design: factorial RCT Country: USA Recruitment: callers to Wisconsin Tobacco Quitline from 1 April 2010 to 15 June 2010	
Participants	987 smokers; aged ≥ 18 years, ≥ 10 cigarettes per day, willing to quit in next 30 days 42.4% men, average age 41.9, average cigarettes per day 20.7, 85% of participants' time to first cigarette was within 5 minutes, mode category for number of previous quit attempts was 2 to 5	
Interventions	2 x 2 x 2 factorial design. There were 3 intervention components tested (detailed below) that were tested in different combinations, resulting in 8 study groups. 1) Nicotine patch versus nicotine patch plus nicotine gum 2) Two weeks NRT versus 6 weeks NRT 3) Standard counselling versus medication adherence counselling For the purposes of this review, we are interested in comparisons 1 and 2.	
Outcomes	30‐day PPA at 6‐month follow‐up Other: 7‐day PPA at 6‐month follow‐up Validation: none Adverse events: not measured	
Notes	Participants randomised to 6 weeks of NRT were sent an initial shipment of 4 weeks NRT. If they indicated interest in receiving additional NRT during a subsequent call, they were sent an additional 2 weeks supply of NRT. Factorial trial. Tests were carried out for interaction effects and none of these were found to be significant. We have therefore combined study arms to provide 2 comparisons (patch versus patch plus gum and 2‐week versus 6‐week duration). Participants received up to USD 50 for completing follow‐up assessments. Study supported by National Cancer Institute grants 1RC1CA144382 and K05CA139871 Conflicts of interest: "S.S.S. has served in the past 5 years as a co‐investigator on research studies at the University of Wisconsin–Madison that were funded wholly or in part by GlaxoSmithKline and Pfizer. T.B.B. has served as an investigator in the past 5 years on research studies at the University of Wisconsin– Madison that were funded in part by GlaxoSmithKline. T.B., B.M., and S.M.Z. are employees at Alere Wellbeing and also own stock in Alere Wellbeing (formerly Free & Clear, Inc.), an organization providing quitline services in Wisconsin. T.A.M. was employed by and owned stock in Free & Clear prior to being appointed Director of the Office on Smoking and Health, CDC, in September 2010. He was also an unpaid member of the Board of Directors of the nonprofit North American Quitline Consortium. T.A.M. has no current financial disclosures. M.C.F. has served in the past 5 years as an investigator on research studies at the University of Wisconsin‐Madison that were funded wholly or in part by Pfizer, GlaxoSmithKline, and Nabi. From 1997 to 2010, M.C.F. held a University of Wisconsin named Chair for the Study of Tobacco Dependence, made possible by a gift to the university from GlaxoWellcome."	
***Risk of bias***		
**Bias**	**Authors' judgement**	**Support for judgement**
Random sequence generation (selection bias)	Low risk	Quote: "The 2 × 2 × 2 design yielded eight possible treatment combinations; participants were randomly assigned to the eight treatment combinations via a list of randomized numbers generated by SAS Proc Plan (SAS Institute Inc., Cary, NC)"
Allocation concealment (selection bias)	Unclear risk	Quote: "After initial phone screening by quitline registration staff, participants were transferred to a Quit Coach® (trained cessation counselor) at the quitline who completed consent, a baseline survey, enrollment, randomization to treatment, and provision of prequit counseling"
Blinding (performance bias and detection bias) All outcomes	High risk	No blinding of participants. Staff collecting outcome data were not affiliated with the quit‐line, but it is unclear whether they were blind to group allocation
Incomplete outcome data (attrition bias) All outcomes	Low risk	> 50% participants followed up at strictest quit time point. Similar follow‐up between arms

**Table:** Stapleton 1995

***Study characteristics***		
Methods	Study design: parallel RCT Country: UK Setting: primary care	
Participants	1200 smokers considered by general practitioner (GP) to be highly dependent and motivated to give up Average cigarettes per day: 23 to 24	
Interventions	1) Nicotine patch standard dose (15 mg/16‐hour for 18 weeks) 2) Nicotine patch with dose increase to 25 mg at 1 week if required 3) Placebo patch group The nicotine patch groups were further randomised to gradual tapering or abrupt withdrawal at week 12	
Outcomes	Sustained abstinence at 12 months Validation: CO Adverse events: measured at each visit.	
Notes	This study was supported by Kabi Pharmacia AB, Sweden, which also supervised and monitored procedures and data collection in the practices. Medical Research Council and Imperial Cancer Research Fund financially supported the health behaviour unit. Conflicts of interest: not reported	
***Risk of bias***		
**Bias**	**Authors' judgement**	**Support for judgement**
Random sequence generation (selection bias)	Low risk	Quote: "a computer generated list, complied in blocks of six (four active, two placebo)"
Allocation concealment (selection bias)	Low risk	Numbered packages
Blinding (performance bias and detection bias) All outcomes	Low risk	Quote: "Both subjects and their doctors or nurses were blind to whether the dose increase was real or placebo". Study conduct throughout was monitored by clinical research associates of the pharmaceutical company
Incomplete outcome data (attrition bias) All outcomes	Unclear risk	ITT analyses, with losses/failures included as smokers. Number of dropouts not specified

**Table:** TNSG 1991

***Study characteristics***		
Methods	Study design: parallel RCT Country: USA (9 sites) Recruitment: community volunteers (treated at smoking cessation clinics)	
Participants	808 unselected smokers 40% men, average age 43, average cigarettes per day: 31	
Interventions	1) Nicotine patch (21 mg/24‐hour, 6 weeks+) 2) Nicotine patch 14 mg 3) Placebo patch Abstainers at end of week 6 entered a randomised blinded trial of weaning	
Outcomes	Sustained abstinence at 6 months Validation: CO < 8 ppm Adverse events: not reported	
Notes	2 trials pooled and data relating to a 7 mg patch group used in only 1 trial omitted Long‐term (4‐ to 5‐year) follow‐up data reported for 7/9 sites (Daughton 1999). These data are not used in analysis. Study was supported by Alza Corp. Conflicts of interest: "Drs Christen, Hatsukami, Rennard, Lichtenstein, Heatley, Repsher, Fortmann, Killen, Hughes, and Glover and Mr Daughton have received fees from Marion Merrell Dow Inc for consultancies and honoraria for educational activities. Authors employed by Marion Merrell Dow Inc (Drs Rolf and Nowak and Messrs Ackerman and Malone) and those employed by Alza Corp (Drs Causey and Knowles and Mss Voss‐Roberts, Prather, Trunnell and Moos) own shares of company stock. Dr Biglan's spouse owns stock in Alza Corp"	
***Risk of bias***		
**Bias**	**Authors' judgement**	**Support for judgement**
Random sequence generation (selection bias)	Unclear risk	Not stated Quote: "patients were ... randomized", but members of same household received same assignment, with 1 randomly selected for inclusion in the analyses
Allocation concealment (selection bias)	Unclear risk	Not stated
Blinding (performance bias and detection bias) All outcomes	Unclear risk	Described as double‐blind
Incomplete outcome data (attrition bias) All outcomes	Low risk	Quote: "All pts [participants] were included in outcome evaluations except for the excluded members of couples (49 pts) and nine pts with major protocol infractions". Losses and withdrawals were included as treatment failures

**Table:** Tulloch 2016

***Study characteristics***		
Methods	Study design: parallel RCT Country: Canada Recruitment: by advertising (radio, local newspaper and posters), from people presenting to the Quit Smoking programme at the institution, and from referrals by local physicians	
Participants	737 smokers (490 in relevant trial arms); aged ≥ 18 years, ≥ 10 cigarettes per day, willing to make a quit attempt in the next 2 to 4 weeks 53.6% men, average age 48.6, average cigarettes per day 23.2, mean FTND 6.1, average years smoked 31, average number of previous quit attempts 4.6	
Interventions	1) Nicotine patch for 10 weeks beginning on quit day (maximum 21 mg/day or 14 mg/day depending on baseline cigarettes per day, decreasing from week 7) 2) Self‐titrated nicotine patch (maximum 35 mg/day) and ad libitum nicotine gum or inhaler for up to 22 weeks	
Outcomes	Validated continuous smoking abstinence from week 5 to 52 Other measures: validated 7‐day PPA at 52 weeks Validation: expired CO ≤ 9 ppm Adverse events: measured at each appointment (0, 1, 3, 5, 8, 10, 22, 52 weeks). Note treatment lasted either 10 or 22 weeks, depending on arm	
Notes	Funding from the Heart and Stroke Foundation of Ontario (Grant‐in‐Aid #6614). Conflicts of interest: AP and RR have received research grants from Pfizer. AP and BR have been paid for developing and delivering educational presentations for Pfizer. AP is on the advisory board for Pfizer and Johnson & Johnson. Not included in any meta‐analyses as any comparison would be confounded by other factors	
***Risk of bias***		
**Bias**	**Authors' judgement**	**Support for judgement**
Random sequence generation (selection bias)	Low risk	Quote: "After eligibility was confirmed by one of the principal investigators (HT, AP), participants were randomized to receive NRT, NRT+, or VR using a computer‐generated block randomization schedule by a statistical consultant not involved in the trial"
Allocation concealment (selection bias)	Low risk	Quote: "After eligibility was confirmed by one of the principal investigators (HT, AP), participants were randomized to receive NRT, NRT+, or VR using a computer‐generated block randomization schedule by a statistical consultant not involved in the trial"
Blinding (performance bias and detection bias) All outcomes	High risk	Participants not blinded to treatment condition Quote: “The research coordinator collecting follow‐up data at weeks 22 and 52 was blind to treatment condition.”
Incomplete outcome data (attrition bias) All outcomes	Low risk	> 50% followed up at strictest quit time point (152/245 and 171/245). Similar dropout between arms. 15 and 12 participants in the arms of interest were excluded due to death or moving away

**Table:** Tønnesen 1988

***Study characteristics***		
Methods	Study design: parallel RCT Country: Denmark Recruitment: primary care	
Participants	113 low‐ to medium‐dependence smokers, motivated to quit (19 or less on Horn‐Russell scale) 44% men, average age 45, average cigarettes per day 20 60 highly‐dependent smokers 42% men, average age 45, average cigarettes per day 26 to 28	
Interventions	Group A: low/medium dependence 1) Nicotine Gum (2 mg) for 16 weeks 2) Placebo Group B: high‐dependence 1) Nicotine gum 4 mg for 6 weeks then 2 mg 2) Nicotine gum 2 mg	
Outcomes	Sustained abstinence at 12 months (24 months also reported) Validation: CO Adverse events: measured during counselling sessions to end of treatment (either 16 or 20 weeks)	
Notes	"This study was supported in part by a grant from the Danish National Tuberculosis Foundation. A.B. Leo, Halsingborg, Sweden and H. Lundbeck A.S., Denmark supplied the nicotine and placebo chewing gum". Conflicts of interest: not reported	
***Risk of bias***		
**Bias**	**Authors' judgement**	**Support for judgement**
Random sequence generation (selection bias)	Unclear risk	Participants stratified by dependence, then [quote]: "subjects on each list were then randomly assigned to treatment in blocks of two"
Allocation concealment (selection bias)	Unclear risk	Not stated
Blinding (performance bias and detection bias) All outcomes	Unclear risk	Gum was packaged and produced to be indistinguishable between 2 mg, 4 mg and placebo
Incomplete outcome data (attrition bias) All outcomes	Low risk	All participants who attended 1st counselling session were included in analyses, regardless of attendance or level of gum use Only 2/173 were lost to follow‐up

**Table:** Tønnesen 1996

***Study characteristics***		
Methods	Study design: parallel RCT Country: Denmark Recruitment: participants who continued to smoke after participation in 2 previous NRT smoking cessation trials were invited to participate	
Participants	89 smokers: previous failed quit attempts; willing to quit completely 30.3% men; average age: 49.5; average cigarettes per day: 22; average FTND: 6.1; salivary cotinine at baseline: 463.5 ng/mL	
Interventions	1) Nicotine nasal spray: advice to use ad libitum (up to 10 puffs/hour and 80 puffs/day) 2) Nicotine nasal spray: advice to use 1 puff/hour whilst awake Treatment continued for 6 months following quit day, but tapering could be initiated after 3 months	
Outcomes	Continuous smoking abstinence at 12‐month follow‐up (defined as abstinence from week 2 post‐quit day to 12‐month follow‐up); CO‐validated (< 10 ppm) Other abstinence measures: CO‐validated continuous abstinence at 6 months; CO‐validated abstinence allowing for slips (occasionally smoking between 2 visits) at 6 and 12 months Adverse events: measured up to 6 weeks (participants using treatment at this time)	
Notes	Pharmacia AB Consumer Pharma, Helsingborg, Sweden, sponsored the study and analysis of saliva for cotinine levels Conflicts of interest: not reported Despite differing dosing instructions between groups, no difference was observed: quote: “Two dosage regimens were used, however, no difference was observed between the fixed and ad libitum dosing group. With a mean daily dose of 16 mg nicotine, most subjects have in fact used the NNS [nicotine nasal spray] once every hour as prescribed.”	
***Risk of bias***		
**Bias**	**Authors' judgement**	**Support for judgement**
Random sequence generation (selection bias)	Unclear risk	Quote: “This was an open randomized study with active NNS”. No detail on how randomisation achieved
Allocation concealment (selection bias)	Unclear risk	As above
Blinding (performance bias and detection bias) All outcomes	Low risk	Open‐label design – for this comparison, blinding participants was not possible. However, the behavioural support received by the groups was the same and abstinence was biochemically validated, reducing the risk of both performance and detection bias
Incomplete outcome data (attrition bias) All outcomes	Unclear risk	Numbers lost to follow‐up not stated (no response to our request to author for figures)

**Table:** Tønnesen 2000

***Study characteristics***		
Methods	Study design: parallel RCT Country: Denmark Recruitment: referrals to lung clinic	
Participants	446 smokers ≥ 10 cigarettes per day 48% men, average age 49, average cigarettes per day 18	
Interventions	1) 5 mg nicotine patch (placebo) 2) 15 mg (16‐hour) nicotine patch for 12 weeks (up to 9 months on request) 3) Nicotine inhaler (4 to 12/day ad lib) 4) Combination, 15 mg patch and inhaler	
Outcomes	Sustained abstinence at 12 months, (from week 2, paper also reports PPA and with slips rates) Validation: CO < 10 ppm at all visits Adverse events: measured at every follow‐up to 12 months (note: treatment could continue to 12 months)	
Notes	This study was supported by a grant from Pharmacia & Upjohn, Helsingborg, Sweden and the Danish Lung Foundation. Conflicts of interest: not reported	
***Risk of bias***		
**Bias**	**Authors' judgement**	**Support for judgement**
Random sequence generation (selection bias)	Low risk	Quote: "a computer‐generated list with random numbers"
Allocation concealment (selection bias)	Unclear risk	Not stated
Blinding (performance bias and detection bias) All outcomes	Unclear risk	Not used ‐ open‐label trial
Incomplete outcome data (attrition bias) All outcomes	Unclear risk	Non‐attenders or lost to follow‐up were included as smokers

**Table:** Walker 2011

***Study characteristics***		
Methods	Study design: parallel RCT Country: New Zealand Recruitment: eligible callers to New Zealand's national quit‐line July 2007 to January 2009	
Participants	1410 smokers; aged ≥ 18 years, smoked first cigarette within 30 minutes of waking, wanted to quit in next 2 weeks 40% men, average age 41, average cigarettes per day 20, mean FTND 6.3, partner a current smoker 4.2%, at least 1 quit attempt in last year 29%	
Interventions	1) Free NRT selection box (including 1 patch, gum, inhaler, sublingual tablets and oral pouches) providing 1‐week supply in total, followed by 8 weeks free, participant‐selected NRT posted to participants 2) Usual quit‐line care ‐ 2 vouchers (1 sent at baseline and 1 at 4 weeks) for 4 weeks of subsidised NRT patches or gum to be redeemed at pharmacy	
Outcomes	Validated 7‐day PPA (and not using NRT) at 6 months Other measures: self‐reported continuous abstinence (defined as smoking not more than 5 cigarettes since quit date) at 6 months Validation: salivary cotinine ≤ 10 ng/mL Adverse events: serious adverse events only measured to 6‐month follow‐up (treatment duration 8 weeks)	
Notes	Participants randomised to NRT selection box and 8 weeks of preferred NRT were mailed a 4‐week free supply of their chosen 1 or 2 NRT products after the selection box. They were then offered the option of changing their choice of NRT at a 3‐week follow‐up call, prior to the second supply of 4 weeks free NRT being sent out. A very low proportion of participants who claimed to have quit completed verification (34%). We extracted actual verified rates and used these in our main analysis but conducted a sensitivity analysis comparing these figures to data extrapolated from these proportions to the wider trial population, and to non‐verified rates. Results are reported narratively in the text. Funding from Health Research Council of New Zealand and the Heart Foundation of New Zealand. NRT was purchased for the intervention arm of the study from Novartis Consumer Health Australasia Pty Ltd (patch and gum), and provided free by Johnson and Johnson Pacific (inhaler and sublingual tablet) and Niconovum (oral pouch). Conflicts of interest: "All authors declare that no authors have received support from any companies for the submitted work. C.B. and H.M. have previously undertaken research on behalf of NicoNovum, but prior to the purchase of the company by R.J. Reynolds. H.M. has received honoraria for speaking at research symposia and received benefits in kind and travel support from, and has provided consultancy to the manufacturers of smoking cessation medications. N.W. has provided consultancy to the manufacturers of smoking cessation medications, received honoraria for speaking at a research meeting and received benefits in kind and travel support from a manufacturer of smoking cessation medications. M.G. has provided consultancy to the manufacturers of smoking cessation medications. All authors are currently involved in a trial looking at the effect of reduced nicotine cigarettes on smoking cessation. This trial involves the use of cigarettes which have been purchased from Vector Group Ltd."	
***Risk of bias***		
**Bias**	**Authors' judgement**	**Support for judgement**
Random sequence generation (selection bias)	Low risk	Quote: “Participants were allocated randomly by computer, with randomization stratified, using minimization, by ethnicity (Māori versus non‐Māori), sex and level of nicotine dependence (>5 points, ≤5 points on the Fagerström score)”
Allocation concealment (selection bias)	Low risk	Quote: “Participants were allocated randomly by computer, with randomization stratified, using minimization, by ethnicity (Māori versus non‐Māori), sex and level of nicotine dependence (>5 points, ≤5 points on the Fagerström score)”
Blinding (performance bias and detection bias) All outcomes	Low risk	Quote: “Participants were not blinded to treatment allocation”, however blinding of participants would have been impossible. “All research staff involved in outcome assessment were blinded and follow‐up assessments were identical for all participants.”
Incomplete outcome data (attrition bias) All outcomes	Low risk	Lost to follow‐up or withdrawn: 160/706 intervention group, 144/704 control group. Similar between groups, overall < 50%

**CBT:** cognitive behavioural therapy; **CO:** carbon monoxide in exhaled air; **EOT:** end of treatment; **FTND:** Fagerström Test for Nicotine Dependence; **HSI:** Heaviness of Smoking Index; **ITT:** intention‐to‐treat; **OR:** odds ratio; **PPA:** point prevalence abstinence; **ppm:** parts per million; **RCT:** randomised controlled trial; **TQD:** target quit date

### Characteristics of excluded studies [ordered by study ID]

**Table:**

Study	Reason for exclusion
ACTRN12612001210864	All arms received the same NRT and instructions, but some were told that there were benefits of long‐term NRT use. Therefore, between‐group differences were purely in the information provided.
Aubin 2006	Short‐term experimental cross‐over study of the effect of different types of nicotine patch on sleep and smoking urges. Abstinence not measured and length of follow‐up too short
Baker 2021	Despite the factorial study design, an intervention component (varenicline duration) was not balanced between studies arms with varying durations of NRT use.
Berlin 2012	Main comparator was the elective monoamine oxidase (MAO)‐B inhibitor, EVT 302. Groups receiving NRT received the same dosing and administration across groups
Carpenter 2011	Measured effect of providing NRT samples on participants not initially motivated to quit. Participants were encouraged but not required to make a practice quit attempt. Intervention participants were provided with up to 2 boxes of nicotine lozenges.
Chan 2010	Measured effect of counselling plus 2 weeks of free NRT. No data on whether control group also used NRT; unclear if outcome due to counselling or free NRT
Cook 2016	Participants were not motivated or were unwilling to quit at recruitment, or both.
Cook 2021	Participants were not motivated or were unwilling to quit at recruitment, or both.
Dey 1999	Compared free and paid prescription for nicotine patch. Only 14 weeks follow‐up
Etter 2009	Differences in the behavioural intervention (not just NRT) between arms, making it impossible to attribute any effect to use of NRT. For this reason, the study does not meet review inclusion criteria. It was included in Stead 2012, but has been removed for this update.
Fagerström 1993	Short‐term cross‐over trial. Endpoint was withdrawal symptoms not cessation
Fagerström 1997	Short‐term cross‐over trial of different types of NRT. For 2 weeks, participants could choose a method; for other 2 weeks, they were randomly assigned to 1 of gum, patch, spray, inhaler or tablet. Smoking reduction assessed
Fagerström 2000	Short‐term cross‐over trial comparing 2 nicotine delivery devices
Ferguson 2015	Standard nicotine patch treatment versus pre‐quit patch versus varenicline. Follow‐up of fewer than 6 months (10 weeks)
Hajek 1999	Follow‐up of fewer than 6 months. There were no significant differences in 12‐week abstinence rates between gum, patch, spray or inhaler groups.
Haustein 2003	Trial of nicotine gum for smoking reduction in people not making a quit attempt. See Cochrane Review of harm reduction interventions (Lindson‐Hawley 2016)
Hollands 2013	Intervention was informing participants that their oral NRT dose was matched to their phenotype versus genotype; NRT dose was actually the same across groups
Hughes 1989	No long‐term follow‐up; primarily a trial of the effect of instructions
Hughes 2010	Differences in the behavioural intervention (not just NRT) between arms, making it impossible to attribute any effect to use of NRT. For this reason, study does not meet review inclusion criteria. It was included in Stead 2012, but has been removed for this update.
Jibrail 2010	Only 12 weeks of follow‐up. Study of NRT for smoking abstinence and relationship between C‐reactive protein and depressed mood during nicotine abstinence
Kozak 1995	Open‐label study in which smokers with higher nicotine dependence scores were given higher patch doses
Kras 2010	Study of NRT and *Hypericum perforatum* (St. John's wort) extract. Only 10 weeks of follow‐up
Landfeldt 1998	Only 12 weeks of follow‐up reported in abstract. No evidence of benefit from combining patch and nasal spray compared to nasal spray alone
Leischow 1999	Behavioural support differed between arms, confounding effect of NRT
Leischow 2004	Behavioural support differed between arms, confounding effect of NRT
Lu 2017	Pre‐quit nicotine patch versus standard patch versus varenicline. Follow‐up of fewer than 6 months (4 weeks)
Marsh 2005	Only 3 months of follow‐up; safety study comparing 4 mg lozenge to 4 mg gum
McNeil 2007	Only 3 months of follow‐up. Comparison of patch and nasal spray (n = 51) versus nasal spray alone (n = 50). Sustained abstinence rates 18% in each group. Used in a sensitivity analysis of combination therapies
McRobbie 2010	Short‐term cross‐over study assessing withdrawal symptoms and user satisfaction
Minneker 1989	Only 9 weeks of follow‐up
NCT00985985	4‐arm study of 2 mg lozenge versus placebo and 4 mg lozenge versus placebo. However, participants were not randomised to 4 mg or 2 mg lozenge; rather, low‐dependency smokers were allocated to 2 mg lozenge and high‐dependency smokers were allocated to 4 mg lozenge.
NCT01592695	Participants received tailored pharmacotherapy in both study arms. The intervention being tested was the type of behavioural support.
NCT01892813	Participants received tailored pharmacotherapy in both study arms. The intervention being tested was the type of behavioural support.
NCT02147132	Has study arms allowing comparison of standard NRT use and long‐term NRT use; however, only short‐term follow‐up planned (8 weeks)
NCT02271919	Has study arms allowing comparison of combination versus single‐form NRT; however, only short‐term follow‐up planned (12 weeks)
NCT04946825	No eligible comparator as NRT components matched between study arms
Oncken 2009	Study of short‐term effects (4 days) of NRT (nicotine patch and nicotine nasal spray) in pregnant smokers
Pomerleau 2003	Compared extended treatment (18 weeks) to 10‐week treatment with nicotine patch. No follow‐up beyond 18 weeks
Sachs 1995	Only 6 weeks of follow‐up
Schneider 2004	Short‐term cross‐over study testing 5 nicotine treatments. Participants used each medication on rising for half a day and resumed smoking each afternoon.
Schneider 2008	Outcome was craving and withdrawal, not abstinence
Shahab 2011	Short‐term cross‐over trial of withdrawal symptom relief
Shiffman 2000a	Compared 10 and 6 weeks of patch treatment without longer follow‐up. Main outcome was craving and withdrawal
Shiffman 2000b	Comparison between 24‐hour and 16‐hour patches. Assessment of craving and abstinence over 2 weeks
Shiffman 2002	Not a randomised trial. Compared prescription and over‐the‐counter patch in different populations using different methods
Sutherland 1999	Only 3 months of follow‐up. Comparison of patch and nasal spray (n = 104) versus patch alone (n = 138) or nasal spray alone (n = 138). Used in a sensitivity analysis of combination therapies
Tundulawessa 2010	Only 4 weeks of follow‐up
Vikhireva 2003	Trial of free choice of NRT product versus assigned NRT product; no control group
Vinci 2021	Participants were not motivated to quit
Williams 2007	Only short‐term outcomes reported in conference abstract. Trial terminated early when no benefit of higher dose detected in interim analysis
Wright 2018	Ineligible intervention

**NRT:** nicotine replacement therapy

### Characteristics of ongoing studies [ordered by study ID]

**Table:** NCT03538938

Study name	Improving Quitline Support Study: optimizing remotely delivered smoking cessation services for low‐income smokers
Methods	Four factor (2 x 2 x 2 x 2) factorial randomised controlled trial Country: USA Recruitment: at the follow‐up call 4 to 18 months after participating in the standard Wisconsin Tobacco Quit Line (WTQL) programme
Participants	1600 smokers still smoking 4 to 18 months after standard WTQL treatment; 18+ years old; uninsured, covered by a Medicaid programme, or has no more than high school education
Interventions	2 x 2 x 2 x 2 factorial design (16 possible treatment combinations): 1. Quit‐line counselling intensity 1‐call quit‐line counselling 4‐call quit‐line counselling 2. NRT intensity Nicotine patch (< 10 cigarettes per day = 14 mg; ≥ 10 cigarettes per day = 21 mg) for 2 weeks Nicotine patch and lozenge (< 10 cigarettes per day = 2 mg; ≥ 10 cigarettes per day = 4 mg) for 2 weeks. 3. SmokefreeTXT interactive text messages (up to 5 messages per day for up to 2 weeks prior to the target quit day and 6 weeks following the target quit day) Enrolled in programme Proactive information about enrolling 4. Financial incentives for treatment engagement Financial incentives No financial incentives
Outcomes	7‐day PPA at 6 months Validation: saliva sample for cotinine testing with a value of < 4 ng/mL Self‐reported abstinence at 12 weeks, continuous abstinence between 1 and 6 months Adverse events: not listed as an outcome
Starting date	7 June 2018
Contact information	Danielle E McCarthy, University of Wisconsin Center for Tobacco Research and Intervention, School of Medicine and Public Health
Notes	Sponsor: Massachusetts General Hospital Estimated study completion date: 1 January 2023

**Table:** NCT03611881

Study name	Assessing the integration of tobacco cessation treatment into lung cancer screening
Methods	Three factor (2 x 2 x 2) factorial randomised controlled trial Country: USA Recruitment was of people scheduled to undergo routine computed tomography (CT) lung cancer screening at participating Mass General Brigham Health Care System lung cancer screening sites
Participants	640 smokers (smoked in last 30 days) scheduled to undergo routine CT lung cancer screening; aged 50 to 80 years; 20+ pack/years
Interventions	8 arms combining 3 factors: 1. Behavioural phone or videoconferencing counselling to promote smoking cessation 4 weeks of counselling 8 weeks of counselling 2. Nicotine patch in a tapering dose of 21 mg, 14 mg, 7 mg 2 weeks of patch use 8 weeks of patch use 3. Counsellor‐facilitated referral to a community‐based programme to address social needs Referral No referral
Outcomes	7‐day PPA at 6 months Validation: self‐reported Self‐reported 7‐day PPA at 3 months Adverse events: not listed as an outcome
Starting date	8 April 2019
Contact information	Elyse Park, PhD, Associate Professor of Psychiatry, Massachusetts General Hospital, (617) 724‐6836; epark@mgh.harvard.edu
Notes	Estimated study completion date: 1 February 2023

**Table:** NCT04188873

Study name	Optimized chronic care for smokers: developing and implementing integrated clinical and systems interventions in primary care ‐ cessation trial
Methods	Four factor (2 x 2 x 2 x 2) factorial randomised controlled trial Country: USA Recruitment: from primary care
Participants	608 participants; > 18 years old, smoking > 4 cigarettes per day for the previous 6 months
Interventions	2 x 2 x 2 x 2 factorial design (16 possible treatment combinations): 1) Medication type (varenicline versus combination NRT) 2) Preparation medication (4 weeks versus standard) 3) Medication duration (extended (24 weeks) versus standard (12 weeks)) 4) Counselling (intensive versus minimal)
Outcomes	Biochemically‐verified 7‐day PPA at 12 months after target quit date Validation: CO < 5 ppm Adverse events: not listed as an outcome
Starting date	10 December 2020
Contact information	Megan E Piper, University of Wisconsin, Madison 608‐265‐5472; mep@ctri.wisc.edu
Notes	Sponsor: University of Wisconsin, Madison Estimated study completion date: 1 August 2024

**Table:** Zawertailo 2020

Study name	Personalized dosing of nicotine replacement therapy versus standard dosing for the treatment of individuals with tobacco dependence
Methods	Parallel randomised controlled trial Country: Canada Recruitment: from community and smoking cessation treatment clinics at 2 study sites, using pamphlets, posters, radio and social media
Participants	500 smokers of 10+ cigarettes per day; aged 18 to 75 years, interested in using nicotine patch and quitting within next 30 days
Interventions	2‐week run‐in period using 21 mg nicotine patch daily. Those who have not stopped smoking at the end of 2 weeks are randomised to: 1) Daily 21 mg NRT patch plus placebo patch (same titration, maintenance and tapering regime as intervention arm) 2) Daily 21 mg NRT patch plus additional NRT patch at a dose based on tolerability and cigarettes per day for 5 weeks of titration and 5 weeks of maintenance, then tapering down by 7 mg/week. Both study arms receive brief behavioural support weekly.
Outcomes	Continuous abstinence from week 9 to weeks 26 and 52 Validation: urinary cotinine measurement Adverse events: not listed as an outcome
Starting date	January 2018
Contact information	Peter Selby, Centre for Addiction and Mental Health, Toronto peter.selby@camh.ca
Notes	Sponsor: Canadian Cancer Society Research Institute Clinicaltrials.gov ID: NCT03000387. Estimated primary completion date: December 2022

**CO:** carbon monoxide in exhaled air; **PPA:** point prevalence abstinence; **ppm:** parts per million

## Differences between protocol and review

During the peer review process, a content reviewer suggested that post hoc sensitivity analyses excluding studies that enroled vulnerable participants (e.g. those with alcoholism or psychiatric disorders) would be useful. We performed these sensitivity analyses and reported results where appropriate in Effects of interventions.
